# Curvature-Dependent Electrostatic Field as a Principle for Modelling Membrane-Based MEMS Devices. A Review

**DOI:** 10.3390/membranes10110361

**Published:** 2020-11-21

**Authors:** Mario Versaci, Paolo di Barba, Francesco Carlo Morabito

**Affiliations:** 1 DICEAM Department, “Mediterranea” University, I-89122 Reggio Calabria, Italy; morabito@unirc.it; 2 Dipartimento di Ingegneria Industriale e dell’Informazione, University of Pavia, I-27100 Pavia, Italy; paolo.dibarba@unirc.it

**Keywords:** electrostatic membrane MEMS devices, electrostatic actuator, boundary non-linear differential problems, singularities, curvature, ghost solutions, stability, optimal control

## Abstract

The evolution of engineering applications is increasingly shifting towards the embedded nature, resulting in low-cost solutions, micro/nano dimensional and actuators being exploited as fundamental components to connect the physical nature of information with the abstract one, which is represented in the logical form in a machine. In this context, the scientific community has gained interest in modeling membrane Micro-Electro-Mechanical-Systems (MEMS), leading to a wide diffusion on an industrial level owing to their ease of modeling and realization. Physically, once the external voltage is applied, an electrostatic field, orthogonal to the tangent line of the membrane, is established inside the device, producing an electrostatic pressure that acts on the membrane, deforming it. Evidently, the greater the amplitude of the electrostatic field is, the greater the curvature of the membrane. Thus, it seems natural to consider the amplitude of the electrostatic field proportional to the curvature of the membrane. Starting with this principle, the authors are actively involved in developing a second-order semi-linear elliptic model in 1D and 2D geometries, obtaining important results regarding the existence, uniqueness and stability of solutions as well as evaluating the particular operating conditions of use of membrane MEMS devices. In this context, the idea of providing a survey matures to discussing the similarities and differences between the analytical and numerical results in detail, thereby supporting the choice of certain membrane MEMS devices according to the industrial application. Finally, some original results about the stability of the membrane in 2D geometry are presented and discussed.

## 1. Introduction

Recent industrial guidelines direct researchers and designers towards the development of low-cost devices to combine physical properties with low-level machine languages. Thus arises the need to design sensors and actuators to meet the multiple requirements of the most widespread industrial, civil and biomedical applications [1,2,3]. In this context, static and dynamic Micro-Electro-Mechanical-Systems (MEMS) technology has matured, especially in domains where miniaturized and integrated electromechanical systems are required [4,5,6]. Moreover, MEMS represent one of the most important achievements of engineering on an industrial scale [5,6]. Currently, the industrial applications of MEMS devices are extremely varied, from applications in the biomedical domain [2,3,7] and thermally driven systems [8,9] to elastic structures [1,10] gaining wide acclaim, owing to both coupled thermal-elastic systems [5,8,9] and electrostatic-elastic systems [4,5,11] for industrial applications. The MEMS technology makes it possible to integrate both electronic circuits and opto-mechanical devices on the same silicon substrate [12,13], employing manufacturing technologies similar to those used for the realization of integrated circuits. The dimensions of an MEMS device generally vary between a few μms and 1 mm, while its individual components vary between 11 mm and μms [4,5,11,14]. The most widely used MEMS devices belong to the class of the electrostatic-elastic systems [5,11], which consist of two parallel plates, one fixed and the other deformable [5,14,15]. A voltage *V* is applied, and the deformable plate moves. Often, many applications require that a membrane replace the deformable plate, reducing the inertial effects [16,17,18,19,20,21,22,23,24]. Usually, the models use the deflection of the membrane, *u*, as an independent variable [16,17,18,19,20,21,22,23,24]. If, on the one hand, the demand for MEMS devices is strong, on the other hand, it is not always possible to formulate models that are easy to implement [5,11,14,15]. Further, these formulations hardly allow resolution in closed form [16,17,18,19,20,21,22,23,24], so one must be satisfied with obtaining conditions that ensure both existence and uniqueness of the solution [14,16,17,20]. The alternative is the numerical approach, which, although interesting, does not prevent us from being present for ghost solutions [17,19,21,22,23]. Thus, analytical approaches and numerical techniques work together to obtain solutions, respecting the analytical conditions that guarantee existence and uniqueness of the solution without ghost solutions [17,19,21,22,23]. In this context, the experience of the authors in the field of modeling electrostatic MEMS membrane devices with strong non-linearity has grown [16,17,18,19,20,21,22,23,24]. In particular, by combining the physics of the problem with important results of differential geometry [25] analytical results have been obtained in terms of the existence and uniqueness of the solution in some cases [16,17,18,19,20,21,22,23,24] and numerical solutions in the absence of ghost solutions in other cases [16,17,18,19,20,21,22,23,24]. However, the conditions that guarantee existence and uniqueness are often independent of the electromechanical properties of the membrane material, making the studied models unattractive from an industrial point of view [16,20,24,26]. All these works start from the physical observation that the electrostatic field, E, on the membrane is orthogonal to the tangent line to the membrane at the point considered. Moreover, the greater the |E|, the more the membrane will deform, thus, it appeared legitimate in these works to consider |E| proportional to the curvature *K* of the membrane. This made it possible to obtain, both in 1D and 2D geometries with circular symmetries, the second order semi-linear elliptic differential models that, although not explicitly resolvable, easily allow algebraic conditions to be obtained, ensuring the existence and uniqueness of the solution [16,20,26]. Further, regarding 1D geometry, this approach allowed for a differential model wherein any singularities, typical of models known in literature, do not appear explicitly [16]. Moreover, when *V* is applied, the membrane risks touching the upper plate; therefore, stability and optimal control problems were solved to achieve a range of possible values of *V* to achieve the mechanical inertia of the membrane and the maximum value permissible for *V*. These problems, concerning 1D geometry, have been solved and presented in [26], while for geometry 2D, we present the original results in the present study. Finally, a comparison of the achieved results is discussed. Once the analytical studies in 1D and 2D geometries have been compared, numerical approaches, such as the shooting procedure, the Relaxation procedure, and the Keller–Box Scheme for recovering *u* in both geometries, studied in [17,19,21,22,23], are presented and compared.

This survey is structured as follows. The first section discusses some theoretical backgrounds (Section 2) where the well-known Cassani-d’O-Ghoussoub model is presented (a sort of a milestone in membrane MEMS devices). Section 3 presents the physical-mathematical approach exploiting the proportionality between |E| and *K* in order to achieve the differential model for a 1D membrane MEMS Device. Section 4 presents the proposed model in general terms in a way that well-known general results can be applied to it. Following this, a 2D circular membrane MEMS Device model is presented in Section 5 using concepts based on mean curvature and differential geometry. Thereafter, a comparison between the algebraic conditions ensuring the existence of at least one solution for both 1D and 2D models is presented and discussed in Section 6, highlighting which of the two algebraic conditions is stronger than the other. Thus, the uniqueness of the solution for both 1D and 2D models is discussed in Section 7, providing food for thought and a comparison between the results obtained by both geometries in order, followed by a discussion of the conditions ensuring the simultaneous existence and uniqueness in both geometries (Section 8). Section 9 deals with the stability and the optimal control problems in 1D geometry, highlighting the range of possible values for *V* and the limitations concerning the *V*, which is necessary to win the mechanical inertia of the membrane and the maximum permissible value of *V*. Moreover, some remarks about the potential energy in 1D geometry are presented and discussed. Some original results of stability and optimal control in 2D geometry are also discussed in Section 10; the relative range of values of *V* is shown together with the limitations for the minimum *V* to overcome the membrane inertia and the maximum admissible *V*. Then, in the same section, relative to 2D geometry, some useful considerations on the values of *V* maximizing the energy variation are considered and compared with the results obtained in 1D geometry. Following this, numerical approaches for recovering the membrane profile in 1D geometry are discussed in Section 11, providing interesting results in terms of convergence areas and ghost solutions. In Section 12, the most important results obtained by applying numerical techniques on the 2D model are discussed, also highlighting the cases in which, in addition to the non-convergence of the procedure, the phenomena of instability are highlighted. Then, Section 13 offers a comparison between the limitations of the mechanical stress values obtained in both the 1D and 2D formulations. Finally, the conclusion and some future perspectives are presented in the last section of this survey. To facilitate the reading of the survey, the proofs relating to the results known in the literature are reported in the appendices, while proofs of the original results are reported in the text of the survey.

## 2. Some Theoretical Backgrounds

The starting point is a well-known dimensionless steady-state model, studied in [5,15]. It considers an MEMS composed of two parallel plates: one fixed and the other deformable, but clumped at the boundary of a region Ω=[−0.5,0.5] (dimensionless conditions). When a voltage drop is applied, the deformable plate deflects from the rest state (characterized by u(x)=0) towards the fixed plate (lower plate), located at height h=1. The profile u(x), in the stationary case, was studied using the well-known fourth-order elliptic model [5,15,16]

(1) $$\left\{\begin{array}{c} \left(h-d^{*}\right)\Delta^{2}u\left(x\right)=\left(\varrho \int_{\Omega }{\left|\nabla u\left(x\right)\right|}^{2}dx+\gamma \right)\Delta u\left(x\right)+\frac{\lambda_{1}f_{1}\left(x\right)}{{\left(1-u\left(x\right)\right)}^{\sigma }\left(1+\chi \int_{\Omega }\frac{dx}{{\left(1-u\left(x\right)\right)}^{\sigma -1}}\right)}\\ u\left(x\right)=\Delta u\left(x\right)-du_{\nu }=0,\;\;\;x\in \partial \Omega ,\;\;d\geq 0,\;\;\;0<u\left(x\right)<1,\;\;\;x\in \Omega \end{array}\right.$$

where $f_{1}$ is a bounded function carrying the dielectric properties of the material constituting the deformable plate; $\lambda_{1}$ is a quantity depending on *V* applied between the plates; ρ, γ and χ are related to the electric and mechanic properties of the material constituting the deformable plate. Moreover, if σ≤2, one can take into account more general Coulomb potential. Moreover, the existence of at least one solution has been studied using the Steklov boundary condition, achieving Dirichlet and Navier boundary ones: $u_{\nu }$ represents the outer normal derivative of *u* on ∂Ω and, if $\hat{d}=0$, one obtains the Navier boundary conditions, while, if $\hat{d}=+\infty$, one obtains the Dirichlet boundary conditions [5,15]. (1) is a generalization of the model studied in [15], with negligible thickness of the deformable plate. Particularly, neglecting inertial and non-local effects (σ=2, ρ=γ=χ=0), (1), becomes:

(2) $$\left\{\begin{array}{c} \Delta^{2}u\left(x\right)=\frac{\lambda_{1}f_{1}\left(x\right)}{{\left(1-u\left(x\right)\right)}^{2}}\\ 0<u\left(x\right)<1\;\;\mathrm{in}\;\Omega ,\;\;\;u=\Delta u\left(x\right)-du_{\nu },\;\;\mathrm{on}\;\partial \Omega ,\;\;d\geq 0. \end{array}\right.$$

### The Cassani-d’O-Ghoussoub Model and Some Theoretical Backgrounds

**Figure 1:**
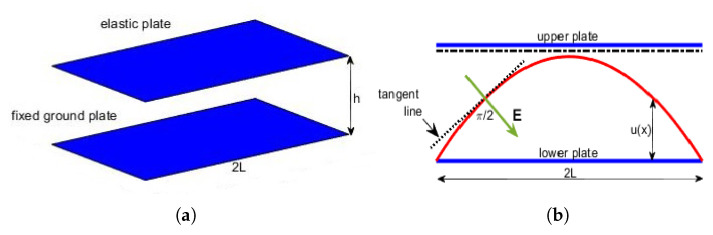
(**a**) The electrostatic-elastic system, (**b**) E orthogonal to the membrane profile.

In $R^{3}$ with a system of Cartesian axes $O^{\prime }x^{\prime }y^{\prime }z^{\prime }$ (see Figure 1a), let us consider an electrostatic-elastic system with length 2L, composed by a pair of parallel plates (one fixed and the other one elastic but clumped at the edges). The plates are located at a mutual distance *h* but lying orthogonal to $z^{\prime }$. When *V* is applied, the elastic plate moves towards the fixed plate (for it V=0). Thus, the electrostatic potential ϕ satisfies Δϕ=0 between the plates, such that ϕ=V on the elastic plate and ϕ=0 on the fixed plate. Thus, indicating by $\Delta_{⊥}$ the Laplacian operator, with respect to $x^{\prime }$ and $y^{\prime }$ only, the deflection $w^{\prime }$ of the elastic plate satisfies the equation [5]:

(3) $$-T\Delta_{⊥}w^{\prime }\left(x\right)+D\Delta_{⊥}^{2}w^{\prime }\left(x\right)=-\frac{\epsilon_{0}}{2}{\left|\nabla \phi \right|}^{2}$$

where *T* is the mechanical tension of the plate *D* is the flexural stiffness and $\epsilon_{0}$ is the permittivity of the free space. In (3), $-\frac{\epsilon_{0}}{2}{\left|\nabla \phi \right|}^{2}$ represents a source term due to E coupling the solution of the elastic problem to the solution of the electrostatic one [5,16]. Exploiting the following scaling factors

(4) $$w\left(x\right)=\frac{w^{\prime }\left(x\right)}{h},\;\;\Phi =\frac{\phi }{V},\;\;x=\frac{x^{\prime }}{2L},\;\;y=\frac{y^{\prime }}{2L},\;\;z=\frac{z^{\prime }}{h},\;\;$$

(3) becomes the following nonlinear coupled partial differential equation system [5,16]:

(5) $$\left\{\begin{array}{c} \epsilon^{2}\Delta_{⊥}\Phi +\frac{\partial^{2}\Phi }{\partial z^{2}}=0\\ -\Delta_{⊥}w\left(x\right)+\delta \Delta_{⊥}^{2}w\left(x\right)=-\lambda^{2}\left[\epsilon^{2}{\left|\nabla_{⊥}\Phi \right|}^{2}+{\left(\frac{\partial \Phi }{\partial z}\right)}^{2}\right]\\ \Phi =1\;\;\mathrm{on}\;\mathrm{elastic}\;\mathrm{plate},\;\;\;\Phi =0\;\;\mathrm{on}\;\mathrm{fixed}\;\mathrm{plate} \end{array}\right.$$

where the importance of tension and rigidity is $\delta =\frac{D}{{\left(2L\right)}^{2}T}$ and the aspect ratio of the system becomes $\epsilon =\frac{h}{2L}$. Finally, setting $\lambda_{1}=\lambda^{2}$, we write:

(6) $$\lambda_{1}=\lambda^{2}=\frac{\epsilon_{0}V^{2}{\left(2L\right)}^{2}}{2h^{3}T}=\varrho V^{2}$$

in which

(7) $$\varrho =\frac{\epsilon_{0}{\left(2L\right)}^{2}}{2h^{3}T}$$

considers the electro-mechanical properties of the material constituting the deformable plate. ϱ, in dimensionless conditions and by (4) becomes $\frac{\epsilon_{0}}{2T}>10^{12}$. If a membrane replaces the deformable plate, the thickness with *D* and δ is negligible. Then, as ϵ→0, the first equation of (5) becomes $\frac{\partial^{2}\Phi }{\partial z^{2}}=0$ so that $\Phi =\frac{z}{w}$ from which the second equation of (5) becomes [5,16]:

(8) $$\left\{\begin{array}{c} \frac{d^{2}w\left(x\right)}{dx^{2}}=\frac{\lambda^{2}}{{\left(w\left(x\right)\right)}^{2}}\;\;\mathrm{in}\;\Omega \\ w(-0.5)=w(0.5)=1. \end{array}\right.$$

If we placing w(x)=1+u(x), (8) becomes $\frac{d^{2}u\left(x\right)}{dx^{2}}=\frac{\lambda^{2}}{{\left(1+u\left(x\right)\right)}^{2}}$ in Ω, with u(−0.5)=u(0.5)=0 and reversing *z* so that the membrane at rest is located on z=0, we write the Cassani-d’O-Ghoussoub model [15]:

(9) $$\left\{\begin{array}{c} \frac{d^{2}u\left(x\right)}{dx^{2}}=-\frac{\lambda^{2}}{{\left(1-u\left(x\right)\right)}^{2}}\;\;\mathrm{in}\;\Omega \\ u(-0.5)=u(0.5)=0 \end{array}\right.$$

We note that with an appropriate setting of the parameters in (1), (9) is easily obtainable. Moreover, condition 0<u(x)<1 in (9) is imperative for the membrane to not touch the fixed plate. Thus, a critical security distance, $d^{*}$, must be taken into account, so that $0<u\left(x\right)\leq h-d^{*}$.

## 3. The Physical-Mathematical Approach: |E| Proportional to the Curvature of the Membrane

### Electrostatic and Mechanical Pressures

Once *V* is applied, an electrostatic pressure, [23,24,27]

(10) $$p_{el}=\frac{1}{2}\frac{\epsilon_{0}V^{2}}{{\left(1-u\left(x\right)\right)}^{2}},$$

takes place. Thus, if *k* is a dimensionless coefficient of proportionality, it makes sense to write $p=kp_{el}$ where *p* is the mechanical pressure which, if its amplitude is sufficient to win the mechanical inertia of the membrane, pushes the membrane towards the fixed plate.

**Remark** **1.** *In* (10) *$\frac{V^{2}}{{\left(1-u\left(x\right)\right)}^{2}}$ is proportional to ${\left|E\right|}^{2}$[5,11,16,27], so that E produces $p_{el}$. Moreover, from* (9)*, considering* (6)*, $\frac{\lambda^{2}}{{\left(1-u\left(x\right)\right)}^{2}}\propto {\left|E\right|}^{2}$.*

Thus, by Remark 1, (9) becomes:

(11) $$\left\{\begin{array}{c} \frac{d^{2}u\left(x\right)}{dx^{2}}=\theta {\left|E\right|}^{2}\;\;\;\mathrm{in}\;\;\Omega =\left[-0.5,0.5\right]\\ \theta \in R^{+},\;u\left(-0.5\right)=u\left(0.5\right)=0. \end{array}\right.$$

**Remark** **2.** *The higher E is, the more the membrane will be curved. Moreover, E, regardless of the deformation of the membrane, is always locally orthogonal to the straight line tangent to the membrane at the point considered (see Figure 1b) [27]. Thus, we consider |E| proportional to the curvature of the membrane [16,17,20,26]:*

(12) |E|=μ(x,u(x))K(x,u(x))

*where K(x,u(x)) is the curvature of the deformed membrane and μ(x,u(x)) is the proportionality function between |E| and K(x,u(x)).*

Therefore, (11), considering Remark 2, becomes:

(13) $$\left\{\begin{array}{c} \frac{d^{2}u\left(x\right)}{dx^{2}}{=-\theta (\mu \left(x,u\left(x\right)\right)}^{2}{\left(K\left(x,u\left(x\right)\right)\right)}^{2}\;\;\;\mathrm{in}\;\;\Omega =\left[-0.5,0.5\right]\\ \theta \in R^{+},\;\;\;u\left(-0.5\right)=u\left(0.5\right)=0,\;\;\;0<u\left(x\right)<h-d^{*}. \end{array}\right.$$

**Remark** **3.** *Physically, $\mu \left(x,u\left(x\right)\right)\in C^{0}\left(\left[-0.5,0.5\right]\right)\times \left[0,1\right)$ [16,17]. Moreover, to prevent the electric discharge between the plates, the membrane must be sufficiently far from the undeformable plate. In other words,*

(14) $$\mu \left(x,u\left(x\right)\right)=\lambda {\left(1-u\left(x\right)-d^{*}\right)}^{-1}$$

where $d^{*}=\frac{\lambda }{\epsilon_{t}}$ with $\epsilon_{t}$ is the dielectric strength of the material constituting the membrane, even if the deflection assumes its maximum deformation [5,11,14].

Finally, by (14), (13) becomes:

(15) $$\left\{\begin{array}{c} \frac{d^{2}u\left(x\right)}{dx^{2}}=-\frac{\theta \lambda^{2}}{{\left(1-u\left(x\right)-d^{*}\right)}^{2}}{\left(K\left(x,u\left(x\right)\right)\right)}^{2}\;\;\;\mathrm{in}\;\;\Omega =\left[-0.5,0.5\right]\\ \theta \in R^{+},\;\;\;u\left(-0.5\right)=u\left(0.5\right)=0,\;\;\;0<u\left(x\right)<h-d^{*}. \end{array}\right.$$

(15) can be studied by considering 1D geometry and 2D circular geometry [16,20]. If the geometry is 1D, K(x,u(x)) assumes the well-known formulation studied in the basic university courses of Calculus [28]. In 2D circular geometry, the formulation obtained is more complex, which requires some precautions [20]. In the remainder of the survey, we will present both models.

## 4. 1D Membrane MEMS Device: The Differential Model

**Figure 2:**
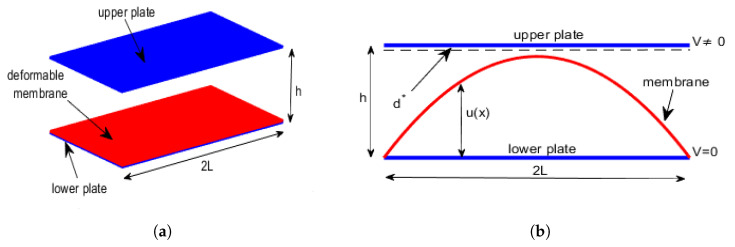
(**a**) Electrostatic Micro-Electro-Mechanical-Systems (MEMS) device, (**b**) typical profile of a MEMS membrane.

K(x,u(x)) in 1D geometry can be easily written as [28] (for geometrical details, see both Figure 2a,b

(16) $$K\left(x,u\left(x\right)\right)=|\frac{d^{2}u\left(x\right)}{dx^{2}}|{\left(1+{\left|\frac{du(x)}{dx}\right|}^{2}\right)}^{-\frac{3}{2}}.$$

Thus, substituting (16) into the equation of (15), we can obtain [16,18]:

(17) $$\left\{\begin{array}{c} \frac{d^{2}u\left(x\right)}{dx^{2}}=-\frac{1}{\theta \lambda^{2}}{\left(1+{\left(\frac{du(x)}{dx}\right)}^{2}\right)}^{3}{\left(h-d^{*}-u\left(x\right)\right)}^{2}\;\;\;\mathrm{in}\;\;\Omega =\left[-0.5,0.5\right]\\ \theta \in R^{+},\;\;\;u\left(-0.5\right)=u\left(0.5\right)=0,\;\;\;0<u\left(x\right)<h-d^{*}. \end{array}\right.$$

**Remark** **4.** *Substituting* (16) *into* (17)*, you would get:*

(18) $$\frac{d^{2}u\left(x\right)}{dx^{2}}+\theta \mu^{2}\left(x,u\left(x\right)\right)|\frac{d^{2}u\left(x\right)}{dx^{2}}{|}^{2}{\left(1+{\left(\frac{du(x)}{dx}\right)}^{2}\right)}^{-3}=0$$

*from which, being u(x)>0, the two following cases could occur:*

*1.*  $\frac{d^{2}u\left(x\right)}{dx^{2}}=0$, thus $\frac{du(x)}{dx}=constant$. Here being u(x) linear, from $\frac{d^{2}u\left(x\right)}{dx^{2}}=0$ it follows that |E|=0. Thus, there exists u(x)≠0 also when |E|=0, so that this condition must be discarted.
*2.*  *Therefore*
  (19) $$\frac{d^{2}u\left(x\right)}{dx^{2}}+\theta \mu^{2}\left(x,u\left(x\right)\right){\left(\frac{d^{2}u\left(x\right)}{dx^{2}}\right)}^{2}{\left(1+{\left(\frac{du(x)}{dx}\right)}^{2}\right)}^{-3}=0$$
  *so that* (17) *makes sense.*

### General Formulation of the 1*D* Model

(17) can be written in a more general manner. In fact, being Ω=[−0.5,0.5] and u:Ω→R, we suppose that $u\left(x\right)\in C^{2}\left(\bar{\Omega }\right)$ is the solution of the following general problem (in the Dirichlet form):

(20) $$\left\{\begin{array}{c} \frac{d^{2}u\left(x\right)}{dx^{2}}+f\left(x,u\left(x\right),\frac{du(x)}{dx}\right)=0\;\mathrm{in}\;\Omega \\ u\left(-0.5\right)=u\left(0.5\right)=0,\;\;\;0<u<h-d^{*} \end{array}\right.$$

where $f\in C^{0}\left(\bar{\Omega }\times R\times R\right)$. Then, considering (17), it follows that $f\left(x,u\left(x\right),\frac{du(x)}{dx}\right)=\frac{1}{\theta \lambda^{2}}{\left(1+{\left(\frac{du(x)}{dx}\right)}^{2}\right)}^{3}{\left(h-d^{*}-u\left(x\right)\right)}^{2}$. Apparently, (17) does not present the singularity that characterizes (9). However, if $u\left(x\right)=h-d^{*}$, from (17) we would achieve $\frac{d^{2}u\left(x\right)}{dx^{2}}=0$. Thus, considering Remark 4, |E|=0 produces a linear u(x), which represents a physically unacceptable condition.

**Remark** **5.** *It is worth noting that when the membrane is too thin, the formation of wrinkles around the membrane is highly probable. However, in this work, in the curvature formulation (see (16)) the basic hypothesis is that the membrane profile $u\left(x\right)\in C^{2}\left(\bar{\Omega }\right)$. This implies that abrupt local variations of the profile are excluded a priori. In other words, wrinkles around the membrane are not allowed. Obviously, removing the hypothesis that $u\left(x\right)\in C^{2}\left(\bar{\Omega }\right)$ will result in formulating the curvature so that any wrinkles around the membrane can be taken into account.*

## 5. 2D Circular Membrane MEMS Device: The Differential Model

Let us consider two parallel disks with radius *R*, mutual distance *h* (in dimensionless condition, h=1) and a circular membrane of the same radius but clumped on the edge of the lower disk, which acts as a support for the membrane. Axial symmetry in the geometry of the membrane inside the device is observed, and considering the *z* axis a rotation axis, *u* of the membrane can be thought of as a surface obtained by rotating the curve *C*, located on the vertical plane rz in the first quadrant and around the axis *z* when 0≤r≤R. Thus, *u* only depends on the radial coordinate *r* so that this problem can be considered as a 1D problem wherein the independent variable *x* is replaced by the radial coordinate *r*.

E between the disks generates a $p_{el}$ (see (10) in which the coordinate *x* is replaced by the radial coordinate *r*), deflecting the membrane. Finally, when the membrane deforms towards the upper disk, the electrostatic capacitance the distance between the membrane and the upper disk varies locally. Therefore, considering the Remark 1 (here is still valid) and just considering the radial part of the Laplace operator [28], model (17) becomes

(21) $$\left\{\begin{array}{c} \frac{d^{2}u\left(r\right)}{dr^{2}}+\frac{1}{r}\frac{du(r)}{dr}=-\theta {\left|E\right|}^{2}\;\;\;\mathrm{in}\;\;\Omega =\left[-0.5,0.5\right]\\ \theta \in R^{+},\;\;\;u\left(-0.5\right)=u\left(0.5\right)=0,\;\;\;0<u\left(x\right)<h-d^{*}. \end{array}\right.$$

However, (12) holds (*x* is replaced by *r*) so that (21) can be written as:

(22) $$\left\{\begin{array}{c} \frac{d^{2}u\left(r\right)}{dr^{2}}+\frac{1}{r}\frac{du(r)}{dr}=-\frac{\theta \lambda^{2}}{{\left(1-u\left(r\right)-d^{*}\right)}^{2}}{\left(K\left(r,u\left(r\right)\right)\right)}^{2}\;\;\;\mathrm{in}\;\;\Omega =\left[-0.5,0.5\right]\\ \theta \in R^{+},\;\;\;u\left(-0.5\right)=u\left(0.5\right)=0,\;\;\;0<u\left(x\right)<h-d^{*}. \end{array}\right.$$

Finally, exploiting the expression of mean curvature [25,29]

(23) $$H\left(r\right)=\frac{1}{2}\left(\frac{1}{r}\frac{du(r)}{dr}+\frac{d^{2}u\left(r\right)}{dr^{2}}\right),$$

(22) becomes:

(24) $$\left\{\begin{array}{c} \frac{d^{2}u\left(r\right)}{dr^{2}}=-\frac{1}{r}\frac{du(r)}{dr}-\frac{{\left(1-u\left(r\right)-d^{*}\right)}^{2}}{\theta \lambda^{2}}\;\;\;\mathrm{in}\;\;\Omega =\left[-0.5,0.5\right]\\ \theta \in R^{+},\;\;\;u\left(-0.5\right)=u\left(0.5\right)=0,\;\;\;0<u\left(x\right)<h-d^{*}. \end{array}\right.$$

For details on how to obtain (24), see Appendix A.

**Remark** **6.** *Also in this case, as highlighted in Remark 5, in the formulation of the mean curvature (23), the hypothesis that $u\left(x\right)\in C^{2}\left(\bar{\Omega }\right)$ remains, so that the possibility of the formation of wrinkles around the membrane is excluded a priori.*

### General Formulation of the 2*D* Model

As in the 1D framework, (24) can be written in a more general way, considering Ω=[−0.5,0.5] and a singularity located at −0.5. Thus, u(r):(−0.5,0.5)→R, with $u\left(r\right)\in C^{2}\left(\bar{\Omega }\right)$. Then, (24) is a particular case of the general following model:

(25) $$\left\{\begin{array}{c} \frac{d^{2}u\left(r\right)}{dr^{2}}+\bar{F}\left(r,u\left(r\right),\frac{du(r)}{dr}\right)=0\\ u\left(0.5\right)=B,\;\;\;\frac{du(0.5)}{dr}=m, \end{array}\right.$$

with $\bar{F}\in C^{0}\left(\left(-0.5,0.5\right]\times R\times R\right)$ and B,m∈R. Moreover, setting

(26) $$\bar{F}\left(r,u\left(r\right),\frac{du(r)}{dr}\right)=\frac{1}{r}\frac{du(r)}{dr}+\frac{{\left(1-u\left(r\right)-d^{*}\right)}^{2}}{\theta \lambda^{2}},$$

B=0, and m=0, we achieve (24). The need to rewrite both the problems 1D and 2D ((17) and (24), respectively) in general terms (as in (20) and (26)) lies in the fact that this generalization allows for using general results on the existence and uniqueness of the solution, as consolidated in the literature, relating to the class of boundary value problems [16,18,20]. Both (17) and (24) do not allow obtaining the solutions explicitly. Therefore, we must be satisfied with obtaining any conditions that ensure the existence and uniqueness of the solution. However, it must be considered that (17) has no obvious singularities, while (24) explicitly manifests a singularity when r=0 [16,18,20].

## 6. A Comparison of the Algebraic Conditions Ensuring the Existence of at Least One Solution for both 1D and 2D Models

### 6.1. On the Existence of at Least One Solution for the 1*D* Model

As highlighted in [16], it is not possible to obtain the solution in explicit form for (17), so one looks for any conditions ensuring existence and uniqueness. Usually, an important tool is the Banach–Caccioppoli fixed point theorem [28], which guarantees the existence and uniqueness of a fixed point for certain maps of metric spaces on themselves, providing a constructive method to find them. This result has the advantage of proving simultaneously the existence and uniqueness of the solution. However, the conditions to be verified are stringent, and, therefore, for certain Boundary Value Problems (BVPs), such verifications are often impractical. In [16], this theorem was not applicable as an alternative way was chosen, obtaining an important result of the existence of the solution based on the Schauder–Tychonoff fixed point theorem and, sequentially, establishing conditions of uniqueness [16,18,28]. In particular, to achieve a result of existence for (17) by this procedure, it is necessary to start by the definition of two suitable functional spaces [16].

**Definition** **1.** Let P be the functional space defined as $\left\{C_{0}^{2}\left[-0.5,0.5\right]:\;0<u\left(x\right)<h-d^{*},\;|\frac{du(x)}{dx}|<H\right\}$ in which $H=\sup|\frac{du(x)}{dx}|$. Moreover, let $P_{1}$ be the functional space defined as $\left\{C_{0}^{1}\left[-0.5,0.5\right]:\;\;0<u\left(x\right)<h-d^{*},\;\;|\frac{du(x)}{dx}|<H\right\}$.

It is well-known that (17) (or its general form (20)), by differentiation, can be transformed into

(27) $$T\left(u\left(x\right)\right)=\int_{-0.5}^{0.5}G\left(x,s\right)f\left(s,u\left(s\right),\frac{du(s)}{ds}\right)ds$$

where $0<u<h-d^{*}$ and G(x,s) is a suitable Green function, the main properties of which are discussed in Appendix B. Then, in [16] the existence of the solution for T(u)=w, with $u\in P_{1}$, was proved exploiting the Schauder–Tychonoff fixed point theorem applied to w=T(u) from *P* to *P*. The following two results were achieved, the proofs of which are detailed in Appendix C and Appendix D, respectively.

**Theorem** **1.** *If*

(28) $$1+H^{6}<\frac{H\theta {\bar{\lambda }}^{2}}{2(h-d^{*})}$$

*then T(u(x)) defined by (27) is an operator from P to P.*

**Theorem** **1.** *(17) admits at least one solution in P.*

### 6.2. On the Existence of at Least One Solution for the 2*D* Model

In [20], the following result of existence of the solution for (24) was achieved; the proof is presented in Appendix E.

**Theorem** **3.** *Let us consider (24) and two twice continuously differentiable functions, $u_{1}\left(r\right)$ and $u_{2}\left(r\right)$, defined on [0,1], with $u_{1}\left(r\right)<u_{2}\left(r\right)$ such that*

(29) $$\frac{d^{2}u_{1}\left(r\right)}{dt^{2}}+\frac{1}{r}\frac{du_{1}\left(r\right)}{dr}+\frac{{\left(1-u_{1}\left(r\right)-d^{*}\right)}^{2}}{\theta \lambda^{2}}>0$$

(30) $$\frac{d^{2}u_{2}\left(r\right)}{dr^{2}}+\frac{1}{r}\frac{du_{2}\left(r\right)}{dr}+\frac{{\left(1-u_{2}\left(r\right)-d^{*}\right)}^{2}}{\theta \lambda^{2}}<0$$

for r∈(0,1). Moreover, $\frac{1}{r}\frac{du(r)}{dr}+\frac{{\left(1-u\left(r\right)-d^{*}\right)}^{2}}{\theta \lambda^{2}}$ is a continuous function (except for r=0), satisfying the Lipschitz condition in U×(−∞,+∞), with $U=\{\left(r,u\right)\;:\;0<r<R\;\;and\;\;u_{1}\left(r\right)\leq u\left(r\right)\leq u_{2}\left(r\right)\}$. If $\frac{du_{1}\left(0\right)}{dr}\geq \frac{du_{2}\left(0\right)}{dr}$, $u_{1}\left(1\right)=u_{2}\left(1\right)=0$, and

(31) $$\theta \lambda^{2}>\frac{d^{*2}}{2V^{2}\epsilon_{0}k},$$

*with k the constant of proportionality between the displacement of the membrane at the center of the plate $u_{0}$ and the mechanical pressure p, there exists at least one solution for (24).*

**Figure 3:**
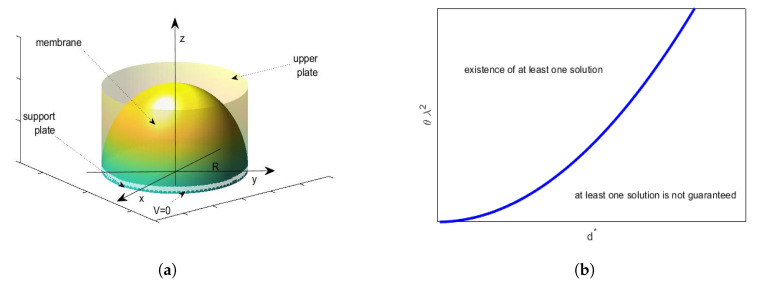
(**a**) Representation of a circular membrane MEMS actuator when its membrane is deformed. (**b**) Area of existence of at least one solution and area where at least one solution is not ensured.

As observed in [20], the greater *k* is, the lower the value of $\theta \lambda^{2}$ will be and, thus, $\frac{d^{2}u\left(r\right)}{dr^{2}}$ will be small, so that the concavity of the membrane rises (the greater $k_{2}$ is, the greater the influence of $p_{el}$ will be). Then, *p* will rise increasing the deformation of the membrane. In addition, Figure 3b shows, in the plane $d^{*}-\theta \lambda^{2}$, the area of existence of at least one solution: the line of equation $\theta \lambda^{2}=\frac{R^{2}d^{*2}}{2V^{2}\epsilon_{0}k}$ in Figure 3b (blue line), separated the area of existence of at least one solution from the area where at least a solution was not ensured.

### 6.3. Conditions Ensuring the Existence of at Least one Solutions for 1D and 2D Geometries: A Comparison

As specified in [16], (28) was the algebraic condition, ensuring the existence of at least one solution for (17). It depended on $\bar{\lambda }$ (linked to *V*) that won the mechanical inertia of the membrane. Moreover, θ took into account the electromechanical properties of the material constituting the membrane. On the other hand, in [20], the specified algebraic condition, (31), ensures the existence of at least one solution for (24). We note that (31) depends on both the mechanical characteristics of the membrane (presence of θ) and *V* (not only the voltage to win the mechanical inertia of the membrane). In this context, it seems interesting to understand which algebraic condition is weaker with respect to the other one. From (28), we write:

(32) $$\theta {\bar{\lambda }}^{2}>\frac{2\left(h-d^{*}\right)\left(1+H^{6}\right)}{H}$$

and being $\lambda^{2}>{\bar{\lambda }}^{2}$, from (32), it makes sense to write:

(33) $$\theta \lambda^{2}>\frac{2\left(h-d^{*}\right)\left(1+H^{6}\right)}{H}$$

so that, from both (31) and (33), we achieve

(34) $$\theta \lambda^{2}>\frac{2\left(h-d^{*}\right)\left(1+H^{6}\right)}{H}\;\;\;\;\mathrm{and}\;\;\;\theta \lambda^{2}>\frac{d^{*2}}{2V^{2}\epsilon_{0}k}.$$

The following results holds.

**Proposition** **1.** *From (34), then*

(35) $$\frac{d^{*2}}{2V^{2}\epsilon_{0}k}<\frac{2\left(h-d^{*}\right)\left(1+H^{6}\right)}{H}$$

*is verified.*

**Proof.**  Inequality (35) is immediately verified by substituting the numerical values for each parameter. In other words, posing $d^{*}\approx 10^{-9}$, $H\approx 10^{2}$ so that $1+H^{6}\approx 10^{12}$ and setting $\epsilon_{0}\approx 8.85\times 10^{-12}$, (35) holds. □

**Remark** **7.** *By Proposition 1, the algebraic condition ensuring the existence of at least one solution in 2D geometry is stronger than the algebraic condition in 1D geometry because, in 2D geometry, not only is V considered necessary to overcome the mechanical inertia of the membrane but also the other possible values of V. This is confirmed by the fact that in 1D geometry, V is not explicitly present. Moreover, in 1D geometry, H in (35) exceeds the $\inf\{\theta \lambda^{2}\}$ with a consequent increase of $\theta \lambda^{2}$ making the numerical results unrealistic.*

## 7. On the Uniqueness of the Solution for both 1D and 2D Models

### 7.1. On the Uniqueness of the Solution for the Model (17)

In [16], the uniqueness of the solution for (17) was proved to be always guaranteed as stated by the following result, the proof of which is detailed in Appendix F.

**Theorem** **4.** *∀H>0 the solution for (17) is unique. Moreover, the following properties hold:*

*(1)*  *∀x∈[−0.5,0.5], $\left|u^{\prime }\left(x\right)|\leq |\frac{du(0.5}{dx}|=|\frac{du(-0.5)}{dx}\right|$;*
*(2)*  *u(x) is symmetric with respect to the origin;*
*(3)*  *$u\left(x\right)\in C^{\infty }\left(\left[-0.5,0.5\right]\right)$;*
*(4)*  *u(x) is analytical.*

In [16], the uniqueness of the solution for (17), being always guaranteed (see Theorem 4), did not depend on the electromechanical properties of the membrane, unlike the existence of the solution that was conditioned by these properties. If the electromechanical properties of the membrane governed the existence of the solution (i.e., the stiffer the material, the more difficult it is for the membrane to move towards the non-deformed plate and the more the material accumulates electrical charges, the greater the possibility that |E| is more intense), the uniqueness of the membrane deflection was guaranteed regardless of the capacity to accumulate electric charges and the membrane’s stiffness. Although this has been proved mathematically in [16], physically it appears to be rather lacking. Versaci et al. in [19] have significantly remedied this gap through an algebraic condition governing the uniqueness of the solution for (17) stronger than the algebraic condition governing the existence. In other words, the existence and uniqueness of the solution for (17), proved in [19], has a more realistic physical-mathematical meaning than the proof given in [16]. In particular, the result obtained in [19] is as follows (the sketch of the proof is shown in Appendix G).

**Theorem** **5.** *If*

(36) $$1+H^{6}<\frac{\theta \lambda^{2}}{18}$$

*Then, (17) admits the uniqueness of the solution.*

In addition, the uniqueness of the solution for (17) depended on the electromechanical parameters of the membrane, but its inertia did not appear. This confirms that when *V* is applied, the membrane moves, if *V* overcomes the inertia of the membrane. Thus, the condition of existence of at least one solution depends on ${\bar{\lambda }}^{2}$. Nevertheless, the condition that guarantees the uniqueness of the solution (28) is independent of ${\bar{\lambda }}^{2}$.

### 7.2. On the Uniqueness of the Solution for the Model (24)

Unlike (17), the uniqueness of the solution for (24) was not guaranteed. This important result, studied in detail in [20], is condensed in the following Theorem (the proof is detailed in Appendix H).

**Theorem** **6.** *Let us consider (24) and suppose that the conditions of the Theorem 3 are satisfied. Moreover, $u_{1}\left(r\right)$ and $u_{2}\left(r\right)$ satisfy the given boundary conditions. Then, the uniqueness of the solution u(r), such that $u_{1}\left(r\right)\leq u\left(r\right)\leq u_{2}\left(r\right)$, is not ensured.*

## 8. Conditions Ensuring both Existence and Uniqueness

### 8.1. 1D Geometry

In [16,19], the following results have been proved and Appendix I details the proof.

**Theorem** **7.** *Algebraic condition* (36) *ensures both existence and uniqueness of the solution for* (17)*.*

**Remark** **8.** (36), proved in [19], depends on the electromechanical properties of the membrane. In [16] the uniqueness, always guaranteed, did not depend on those properties, since the uniqueness was proved independently of those properties reducing the risk of obtaining ghost solutions. Finally, we note that $h-d^{*}$ does not appear in (36)*; thus, existence and uniqueness are not dependent on the critical distance. Table 1 summarizes these results.*

### 8.2. 2D Geometry

Unlike 1D geometry, reference [20] provided an algebraic condition that guarantees the existence of the solution depending both on $d^{*}$ and *k*. However, uniqueness was not guaranteed. In other words, even if a number of different deflections are allowed, they never reach the upper plate avoiding producing the electrostatic discharge between the two plates. Table 1 summarizes these results.

### 8.3. 1D and 2D Geometries: A Comparison

As for the comparison of the algebraic conditions assuring the existence of the solution for both geometries, in this section we deepen which of the two conditions assuring simultaneously the existence and uniqueness of the solution for both geometries is the weakest. From (36) we can write

(37) $$\theta \lambda^{2}>18\left(1+H^{6}\right),$$

so that, taking into account (31), the following result holds.

**Proposition** **2.** *From* (31) *and* (37)*, it follows that*

(38) $$\frac{d^{*2}}{2V^{2}\epsilon_{0}k}<18\left(1+H^{6}\right).$$

**Proof.**  As for the Proposition 1, it is sufficient to carry out a dimensional analysis to prove (38). □

From Proposition 2 we deduce that the algebraic condition assuring both the existence and the uniqueness of the solution in 2D geometry is more stringent than the one in 1D geometry. Thus, the same observations discussed in Remark 7 continue to apply.

## 9. Stability and Optimal Control Problems in 1D Geometry

In Reference [26] authors studied whether the movement of the membrane in 1D geometry, when *V* is applied, admits stable equilibrium configurations. Furthermore, since the membrane has an inertia while moving and considering that it should not touch the upper plate, in [26], the range of possible values of *V* in 1D geometry was achieved. Finally, using concepts based on the variation of potential energy stored in the device, optimal control conditions were obtained. In this section, we will present the main results obtained in [26] and some original results regarding 2D geometry.

### 9.1. Stability and Optimal Control in 1D Geometry

In [26], (17) was transformed into its corresponding system of two first order differential equations

(39) $$\frac{du_{1}\left(x\right)}{dx}=\bar{f}\left(u_{1}\left(x\right),u_{2}\left(x\right)\right)\;\;\;\wedge \;\;\;\frac{du_{2}\left(x\right)}{dx}=\bar{g}\left(u_{1}\left(x\right),u_{2}\left(x\right)\right).$$

Particularly, it was set $u_{1}\left(x\right)=u\left(x\right)$ and $u_{2}\left(x\right)=\frac{du(x)}{dx}$, it was posed [5,26]:

(40) $$\left\{\begin{array}{c} \frac{du_{1}\left(x\right)}{dx}=u_{2}\left(x\right)=0;\\ \frac{du_{2}\left(x\right)}{dx}=-\frac{1}{\theta \lambda^{2}}{\left(1+{\left(u_{2}\left(x\right)\right)}^{2}\right)}^{3}{\left(1-u_{1}\left(x\right)-d^{*}\right)}^{2}=0 \end{array}\right.$$

in [−0.5,0.5] with $\theta \lambda^{2}\neq 0$, obtaining the equilibrium point

(41) $$\left(u_{1}^{0},u_{2}^{0}\right)=\left(h-d^{*},0\right).$$

The following result about the stability in 1D geometry was achieved in [26]. The proof exploited the first Lyapunov criterion based on the linearization of (40) in the neighborhood of the equilibrium state. For details, see Appendix J.

**Proposition** **3.** *The point* (41) *for* (40) *is an unstable equilibrium configuration.*

In [16], it was highlighted that the unique unstable equilibrium point obtained is, in fact, the point considered to be the most critical because it concerns the value of u(x) (located at x= 0) closest to the upper plate of the device. Since *V* is the main cause of membrane movement towards the upper plate, it is important to know the sup{V}, which ensures that the membrane does not go beyond the unstable equilibrium position. Moreover, having the membrane inertia while moving, in [26], the minimum value of *V* allowing the deflection of the membrane was obtained knowing the range of possible value of *V* to understand, by (10), if the $p_{el}$ obtained leads to instability phenomena. Finally, in [26], it was highlighted that the knowledge of the range of possible values of *V* means to know the range of possible values of $\lambda^{2}$ (see (6)), serving, on the one hand, as a tuning parameter for the device [5] and, on the other, to guarantee the convergence of any numerical approach to recover the profile of the membrane [17,19]. In Section 9.2, Section 9.3 and Section 9.4, some important results presented in [26] have been reviewed. Particularly, Section 9.2 discusses the range of possible values for ${\left(V_{min}\right)}_{inertia}$ to overcome the inertia of the membrane (refer to Appendix K), while Section 9.3 presents some results regarding the ${\left(V_{max}\right)}_{permissible}$ so that the membrane does not reach the upper plate. Finally, Section 9.4 illustrates the relationship between ${\left(V_{min}\right)}_{inertia}$ and ${\left(V_{max}\right)}_{permissible}$ as studied in [26].

### 9.2. $V_{min}$ to Overcome the Inertia of the Membrane in 1D Geometry

**Proposition** **4.** *Regarding* (40)*, if* (36) *holds, then the $V_{min}$ needed to overcome the membrane inertia becomes*

(42) $${\left(V_{min}\right)}_{inertia}>\sqrt{\frac{4Th^{3}\left(h-d^{*}\right)}{\epsilon_{0}\theta }}\sqrt{\frac{1+H^{6}}{H}}.$$

(42) has an interesting physical significance. In fact, as *T* increases (i.e., stretching the membrane more at the edges), a higher value of *V* is required to overcome the inertia. Furthermore, the greater the distance between the plates, the greater *V* will be to overcome inertia.

### 9.3. ${\left(V_{max}\right)}_{permissible}$ in Order That the Membrane Does Not Reach the Upper Plate in 1D Geometry

**Proposition** **5.** *Concerning* (40)*, ${\left(V_{max}\right)}_{permissible}$ is bounded as follows:*

(43) $${\left(V_{max}\right)}_{permissible}<\sqrt{\frac{2\left(h-d^{*}\right)d^{*}}{k\epsilon_{0}}}.$$

From (43), it is easy to deduce that the distance between the plates is decisive in the limitation of ${\left(V_{max}\right)}_{permissible}$. In fact, increasing *h* (distance between the plates) requires a greater *V* to push the membrane towards the upper plate. Furthermore, an increase in *k* reduces the ${\left(V_{max}\right)}_{permissible}$ because *p* generated is higher.

**Remark** **9.** *In [26], to prove* (43)*, indicated by $u_{0}$, the deflection in the center of the membrane, the following algebraic conditions were used.*

(44) $$u_{0}\leq \frac{k\epsilon_{0}V^{2}}{2d^{*}};\;\;\;\;\;\sqrt{k}=\frac{\sqrt{p}}{2\sqrt{p_{el}}};\;\;\;\;\;\frac{k\epsilon_{0}V^{2}}{2d^{*}}<h-d^{*};\;\;\;\;\sqrt{0.5\theta }=\frac{0.5}{h}\sqrt{\frac{0.5\epsilon_{0}}{2hT}}.$$

*Please refer to [26] for details of the proofs.*

We note that, in [26], an interesting range of possible values for *V* was achieved. In particular, the two following propositions detail the contents (See Appendix L).

**Proposition** **6.** *The following inequality holds:*

(45) $$\sqrt{\frac{4Th^{3}\left(h-d^{*}\right)}{\epsilon_{0}\theta }}\sqrt{\frac{1+H^{6}}{H}}<\sqrt{\frac{2\left(h-d^{*}\right)d^{*}}{k\epsilon_{0}}}.$$

**Proposition** **7.** *Thus, the range of admissible values for V to win the mechanical inertia of the membrane and to remain far from the upper plate, is as follows:*

(46) $$\sqrt{\frac{4Th^{3}\left(h-d^{*}\right)}{\epsilon_{0}\theta }}\sqrt{\frac{1+H^{6}}{H}}<V<\sqrt{\frac{2\left(h-d^{*}\right)d^{*}}{k\epsilon_{0}}}.$$

### 9.4. Relationship between ${\left(V_{min}\right)}_{inertia}$ and ${\left(V_{max}\right)}_{permissible}$ in 1D Geometry

Finally, in [26], the relationship between ${\left(V_{min}\right)}_{inertia}$ and ${\left(V_{max}\right)}_{permissible}$ in 1D geometry has been obtained as testified by the following Proposition, the proof of which is detailed in Appendix M.

**Proposition** **8.** *The following inequality holds:*

(47) $${\left(V_{min}\right)}_{inertia}>{\left(V_{max}\right)}_{permissible}\frac{k_{2}h\sqrt{h}}{\sqrt{0.5\epsilon_{0}}}\sqrt{\frac{1+H^{6}}{H}}T.$$

**Remark** **10.** (47) *suggests us a more manageable algebraic condition. In fact, being $h=10^{-9}$, $\epsilon_{0}=8.85\times 10^{-12}$, thus $\frac{h^{2}\sqrt{10}\sqrt{h}}{0.5\sqrt{2}\sqrt{0.5\epsilon_{0}}}\sqrt{\frac{1+H^{6}}{H}}\approx 8.15\times 10^{-16}\ll 1$, so that* (8) *becomes*

(48) $${\left(V_{min}\right)}_{inertia}>0.023k_{2}T{\left(V_{max}\right)}_{permissible}.$$

*Again, as highlighted in [26], $k_{2}\approx 1$ since $p_{el}≃p$ and, moreover, T≈1000 Pa, then* (48) *can be written as:*

(49) $${\left(V_{min}\right)}_{inertia}>{\left(V_{max}\right)}_{permissible}\times 0.023k_{2}T≃{\left(V_{max}\right)}_{permissible}\times 0.023.$$

*Thus,* (A84) *in the proof of Proposition 8 makes physical sense. Moreover, in* (A84)*, $\frac{h^{2}\sqrt{10}\sqrt{d}}{0\sqrt{0.5\epsilon_{0}}}\sqrt{\frac{1+H^{6}}{H}}$ is a constant depending on the geometrical parameters of the device. Therefore, one can write $G=G\left(L,h\right)=\frac{h^{2}\sqrt{10}\sqrt{h}}{\sqrt{2}L\sqrt{\epsilon_{0}L}}\sqrt{\frac{1+H^{6}}{H}}$ so that* (A84) *becomes:*

(50) $$\begin{array}{c} {\left(V_{min}\right)}_{inertia}>{\left(V_{max}\right)}_{permissible}Gk_{2}T \end{array}$$

*from which, considering that $k_{2}\approx 1$, it follows that $\frac{{\left(V_{min}\right)}_{inertia}}{{\left(V_{max}\right)}_{permissible}}>GT$. Thus, fixing T (i.e., having chosen the material constituting the membrane), $\frac{{\left(V_{min}\right)}_{inertia}}{{\left(V_{max}\right)}_{permissible}}$ depends on G that changes with the geometry of the device. Conversely, if one chooses the geometry of the device (i.e., G is fixed), the material constituting the membrane determines $\frac{{\left(V_{min}\right)}_{inertia}}{{\left(V_{max}\right)}_{permissible}}$.*

### 9.5. Some Remarks about the Potential Energy in the Device in 1D Geometry

As shown in [26], if the membrane is at rest, its distance from the upper plate is equal to *h*, $C_{el}=\epsilon_{0}\frac{1}{h}$ and its potential energy is $W_{initial}=\frac{1}{2}CV^{2}=\frac{0.5\epsilon_{0}}{h}V^{2}$. If the membrane deforms, $C_{el}=\epsilon_{0}\int_{-0.5}^{+0.5}\frac{dx}{h-u(x)}$ and the final potential energy becomes $W_{final}=\frac{1}{2}CV^{2}=\frac{1}{2}\epsilon_{0}V^{2}\int_{-0.5}^{+0.5}\frac{dx}{h-u(x)}$. Finally, the total variation of the potential energy, ΔW, is:

(51) $$\Delta W=W_{final}-W_{initial}=\epsilon_{0}V^{2}\left\{\frac{1}{2}\int_{-0.5}^{+0.5}\frac{dx}{h-u(x)}-\frac{0.5}{h}\right\}.$$

Being $h-u\left(x\right)\geq h-d^{*}$, then $\frac{1}{h-u(x)}\leq \frac{1}{h-d^{*}}$. Therefore, from (51), we can write:

(52) $$\begin{array}{c} \Delta W=\epsilon_{0}V^{2}\left\{\frac{1}{2}\int_{-0.5}^{+0.5}\frac{dx}{h-u(x)}-\frac{0.5}{h}\right\}\leq \epsilon_{0}V^{2}\left\{\frac{0.5}{h-d^{*}}-\frac{0.5}{h}\right\}=\epsilon_{0}V^{2}\left\{\frac{0.5d^{*}}{h(h-d^{*})}\right\}. \end{array}$$

In [26] was proved that

(53) $$\Delta W\leq \epsilon_{0}\left\{\frac{0.5d^{*}}{h(h-d^{*})}\right\}V^{2}<2\epsilon_{0}\left\{\frac{0.5d^{*}}{h(h-d^{*})}\right\}\frac{\left(h-d^{*}\right)d^{*}}{k}=\frac{2{\left(d^{*}\right)}^{2}0.5\left(h-d^{*}\right)}{kh(h-d^{*})}.$$

and

(54) $$\Delta W>\epsilon_{0}\left\{\frac{0.5d^{*}}{h(h-d^{*})}\right\}V^{2}>\frac{8d^{*}Th^{3}\left(h-d^{*}\right)}{h(h-d^{*})\theta }\frac{1+H^{6}}{H}$$

which combined with each other it follows (see [26]):

(55) $$\frac{8d^{*}Th^{3}\left(h-d^{*}\right)}{h(h-d^{*})\theta }\frac{1+H^{6}}{H}<\Delta W<\frac{{\left(d^{*}\right)}^{2}\left(h-d^{*}\right)}{kh(h-d^{*})},$$

which in dimensionless conditions became:

(56) $$\frac{7524T}{\theta }<\Delta W<\frac{0.005}{k}.$$

In [26], the value of the *V* maximizing ΔW was achieved, as detailed in the following proposition, and the proof is detailed in Appendix N.

**Figure 4:**
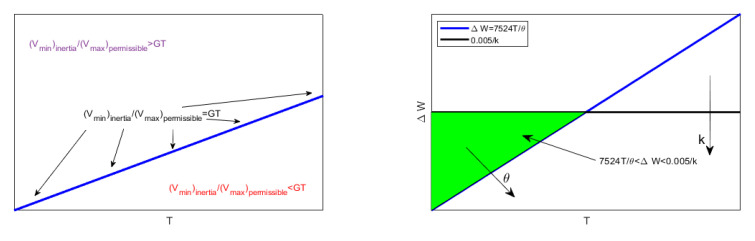
$\frac{{\left(V_{min}\right)}_{inertia}}{{\left(V_{max}\right)}_{permissible}}$ versus *T* and the area of possible values for ΔW according to the (56).

**Proposition** **9.** $V=\sqrt{\frac{2hd^{*}\left(\epsilon_{0}+\sqrt{\epsilon_{0}}\right)}{k\epsilon_{0}^{2}}}$ maximizes ΔW. Moreover (Figure 4):

(57) $$V=\sqrt{\frac{2hd^{*}\left(\epsilon_{0}+\sqrt{\epsilon_{0}}\right)}{k\epsilon_{0}^{2}}}<\sup\left\{{\left(V_{max}\right)}_{permissible}\right\}=\sqrt{\frac{2\left(h-d^{*}\right)d^{*}}{k\epsilon_{0}}}$$

**Remark** **11.** *E, due to the application of V, determines the deflection of the membrane, also determining the amount of ΔW in the device. In such a context, in [26], a limitation for ΔW was obtained starting from |E| as detailed in the following Section.*

### 9.6. A Limitation for ΔW Obtained Starting from |E| in 1D Geometry

From both (9) and (17), and considering that $\frac{\lambda^{2}}{{\left(1-u\left(x\right)\right)}^{2}}=\theta {\left|E\right|}^{2}$, in [26], proved that

(58) $$\Delta W=W_{final}-W_{initial}\leq \frac{\epsilon_{0}}{2\theta^{2}\lambda^{2}}{\left(1+H^{2}\right)}^{3}{\left(h-d^{*}\right)}^{2}-0.5\epsilon_{0}\frac{V^{2}}{h}.$$

In [26], $\frac{\epsilon_{0}}{2\theta^{2}\lambda^{2}}{\left(1+H^{2}\right)}^{3}{\left(h-d^{*}\right)}^{2}-0.5\epsilon_{0}\frac{V^{2}}{h}$ in (58) was compared with $\epsilon_{0}V^{2}\left\{\frac{0.5d^{*}}{h(h-d^{*})}\right\}$ in (52) proving the following proposition (proof detailed in Appendix O).

**Proposition** **10.** *In* (52), $\epsilon_{0}V^{2}\left\{\frac{0.5d^{*}}{h(h-d^{*})}\right\}<\frac{\epsilon_{0}}{2\theta^{2}\lambda^{2}}{\left(1+H^{2}\right)}^{3}{\left(h-d^{*}\right)}^{2}-0.5\epsilon_{0}\frac{V^{2}}{h}$ in (58)*.*

Therefore, in [26], it was proved that condition (52) is stronger than condition (58).

**Figure 5:**
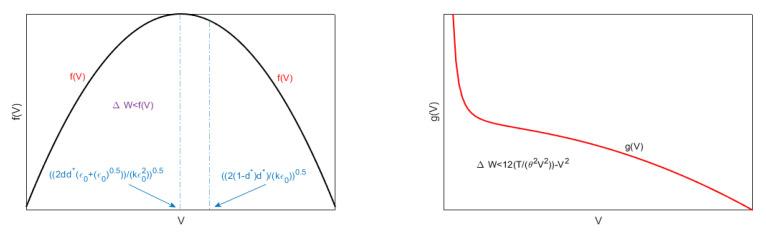
Area of possible values for ΔW according to ΔW<f(V) and ΔW<g(V).

In the following section (Figure 5), some original results on the stability and the optimal control in 2D geometry are discussed in detail.

## 10. Stability and Optimal Control in 2D Geometry

### 10.1. Critical Points and Stability

As (17), (24) has been transformed setting $u_{1}\left(r\right)=u\left(r\right)$ and $u_{2}\left(r\right)=\frac{du(r)}{dr}$:

(59) $$\left\{\begin{array}{c} \frac{du_{1}\left(r\right)}{dr}=u_{2}\left(r\right);\;\;\;\;\frac{du_{2}\left(r\right)}{dr}=-\frac{1}{r}u_{2}\left(r\right)-\frac{{\left(1-u_{1}\left(r\right)-d^{*}\right)}^{2}}{\theta \lambda^{2}}\\ u_{1}\left(R\right)=0;\;\;\;\;\;u_{2}\left(0\right)=0. \end{array}\right.$$

**Proposition** **11.** *Imposing $\frac{du_{1}\left(r\right)}{dr}=\frac{du_{2}\left(r\right)}{dr}=0$ and considering that $\theta \lambda^{2}\neq 0$, from* (59)*, we achieve the unique critical point*

(60) $$\left(u_{1}^{0},u_{2}^{0}\right)=\left(h-d^{*},0\right).$$

*which coincides with the unique equilibrium point in 1D geometry (see* (41)*).*

This is not surprising because 2D geometry is generated by the rotation of a curve lying on a plane. Therefore, for symmetry reasons, the equilibrium positions in 1D and 2D geometries coincide. However, unlike the 1D geometry, here the unique equilibrium point is stable, as detailed in the following proposition.

### 10.2. On the Stability of the Critical Point

**Proposition** **12.** (60) *represents a stable equilibrium configuration.*

**Proof.**  (59), is writable as:

(61) $$\frac{du_{1}\left(r\right)}{dr}=u_{2}\left(r\right)=\bar{f}\left(u\left(r\right)\right)\;\;\;\wedge \;\;\;\frac{du_{2}\left(r\right)}{dr}=-\frac{1}{r}u_{2}\left(r\right)-\frac{{\left(1-u_{1}\left(r\right)-d^{*}\right)}^{2}}{\theta \lambda^{2}}=\bar{g}\left(u\left(r\right)\right)\;$$

in which $u\left(r\right)={\left[u_{1}\left(r\right)\;\;u_{2}\left(r\right)\right]}^{T}$ and $f\left(\dot{u}\left(r\right)\right)=\left(\begin{array}{c} \bar{f}\left(u_{1}\left(r\right),u_{2}\left(r\right)\right)\\ \bar{g}\left(u_{1}\left(r\right),u_{2}\left(r\right)\right) \end{array}\right)=\left(\begin{array}{c} u_{2}\left(r\right)\\ -\frac{1}{r}u_{2}\left(r\right)-\frac{{\left(1-u_{1}\left(r\right)-d^{*}\right)}^{2}}{\theta \lambda^{2}} \end{array}\right)$.

Thus, (59) can be matricially written as:

(62) $$\dot{u}\left(r\right)=f\left(u\left(r\right)\right)$$

where $\dot{u}\left(r\right)={\left[{\dot{u}}_{1}\left(r\right)\;\;{\dot{u}}_{2}\left(r\right)\right]}^{T}.$ To linearize the system, we use the change of variable (A68). Therefore, from (61), considering (A68) and also that $u_{1}^{0}$ and $u_{2}^{0}$ do not depend on *r*, the following can be stated:

(63) $$\frac{du_{1}\left(r\right)}{dr}=\epsilon \frac{d\xi (r)}{dr}=\bar{f}\left(u_{1}\left(r\right),u_{2}\left(r\right)\right)\;\;\;\wedge \frac{du_{2}\left(r\right)}{dr}=\epsilon \frac{d\eta (r)}{dr}=\bar{g}\left(u_{1}\left(r\right),u_{2}\left(r\right)\right).$$

From which, developing in Taylor series both $\bar{f}\left(u_{1}\left(r\right),u_{2}\left(r\right)\right)$ and $\bar{g}\left(u_{1}\left(r\right),u_{2}\left(r\right)\right)$ (neglecting the terms of orders higher than the linear one) and setting $\tau =\sqrt{\xi^{2}+\eta^{2}}$, it follows that

(64) $$\left\{\begin{array}{c} \epsilon \frac{d\xi (r)}{dr}=\bar{f}\left(u_{1}^{0}+\epsilon \left(r\right),u_{2}^{0}+\epsilon \eta \left(r\right)\right)\approx \bar{f}\left(u_{1}^{o},u_{2}^{0}\right)+\epsilon \frac{\partial \bar{f}\left(u_{1}^{0},u_{2}^{0}\right)}{\partial u_{1}}\xi \left(r\right)+\epsilon \frac{\partial \bar{f}\left(u_{1}^{o},u_{2}^{0}\right)}{\partial u_{2}}\eta \left(r\right)+o\left(\tau \right)\\ \epsilon \frac{d\eta (r)}{dr}=\bar{g}\left(u_{1}^{0}+\epsilon \left(r\right),u_{2}^{0}+\epsilon \eta \left(r\right)\right)\approx \bar{g}\left(u_{1}^{o},u_{2}^{0}\right)+\epsilon \frac{\partial \bar{g}\left(u_{1}^{0},u_{2}^{0}\right)}{\partial u_{1}}\xi \left(r\right)+\epsilon \frac{\partial \bar{g}\left(u_{1}^{o},u_{2}^{0}\right)}{\partial u_{2}}\eta \left(r\right)+o\left(\tau \right). \end{array}\right.$$

**Figure 6:**
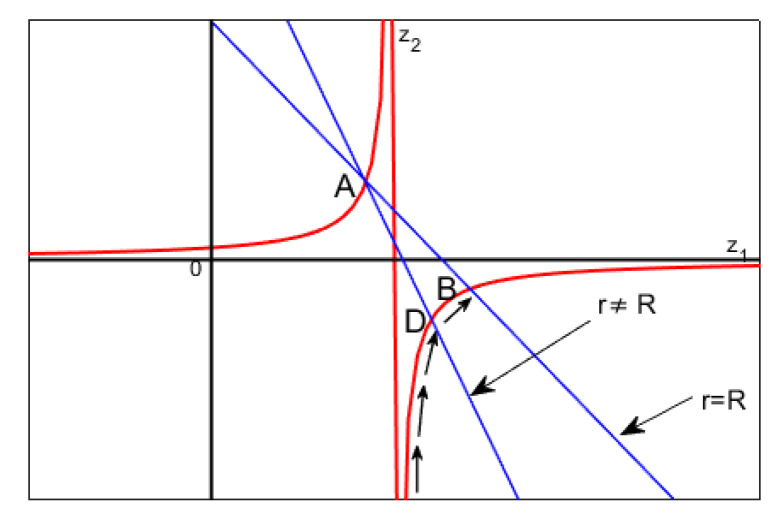
Localization of stability points on the plane $z_{1}z_{2}$.

Being $\bar{f}\left(u_{1}^{0},u_{2}^{0}\right)=\bar{g}\left(u_{1}^{0},u_{2}^{0}\right)=0$, one obtains:

(65) $$\left\{\begin{array}{c} \frac{d\xi (r)}{dr}=\frac{\partial \bar{f}\left(u_{1}^{0},u_{2}^{0}\right)}{\partial u_{1}}\xi \left(r\right)+\frac{\partial \bar{f}\left(u_{1}^{0},u_{2}^{0}\right)}{\partial u_{2}}\eta \left(r\right)=\eta \left(r\right)\\ \frac{d\eta (r)}{dr}=\frac{\partial \bar{g}\left(u_{1}^{0},u_{2}^{0}\right)}{\partial u_{1}}\xi \left(r\right)+\frac{\partial \bar{g}\left(u_{1}^{0},u_{2}^{0}\right)}{\partial u_{2}}\eta \left(r\right)=-\frac{\eta (r)}{r} \end{array}\right.$$

solving which gives $\xi \left(r\right)=C_{1}r^{C_{2}}$ and $\eta \left(r\right)=\frac{C_{3}}{r}$, so that $\xi \left(r\right){\left(\eta \left(r\right)\right)}^{C_{1}}=C_{4}$, which represents a hyperbola, where $C_{1}$, $C_{2}$, $C_{3}$, and $C_{4}$ are all constant. Matricially, (65) is writable as:

(66) $$\dot{z}=Az$$

where $z=\left(\begin{array}{c} \xi (r)\\ \eta (r) \end{array}\right)$; $\dot{z}=\left(\begin{array}{c} \frac{d\xi (r)}{dr}\\ \frac{d\eta (r)}{dr} \end{array}\right)$; $A=\left(\begin{array}{cc} \frac{\partial \bar{f}\left(u_{1}^{0},u_{2}^{0}\right)}{\partial u_{1}} & \frac{\partial \bar{f}\left(u_{1}^{0},u_{2}^{0}\right)}{\partial u_{2}}\\ \frac{\partial \bar{g}\left(u_{1}^{0},u_{2}^{0}\right)}{\partial u_{1}} & \frac{\partial \bar{g}\left(u_{1}^{0},u_{2}^{0}\right)}{\partial u_{2}} \end{array}\right)=\left(\begin{array}{cc} 0 & 1\\ 0 & -\frac{1}{r} \end{array}\right)$. (66) admits stable equilibrium position only if A does not have eigenvalues with positive real part and if any eigenvalues with real part zero have a unit index. Furthermore, if $z_{0}={\left[z_{0,1},z_{0,2}\right]}^{T}$, then $z\left(r\right)=e^{Ar}z\left(0\right)=e^{Ar}z_{0}$. Moreover, |A|=0, then at least one eigenvalue is zero. Thus, the origin is not an isolated equilibrium point, so that there is at least one line (plane) of equilibrium points: the only critical point obtained is represented by (60). Therefore, as the variation of $d^{*}$ changes the equilibrium point, theoretically, infinite equilibrium points, $\forall d^{*}$, could take place. In our case, the eigenvalues of matrix *A* are $\lambda_{1}=0$; $\lambda_{2}=-\frac{1}{r}<0$, (66) is stable. Moreover, since the number of eigenvalues of *A* counted with their algebraic multiplicity is equal to the order of *A* and the geometric multiplicity of each eigenvalue is equal to with the algebraic multiplicity, *A* is diagonalizable. Thus, $e^{Ar}$ can be written as $e^{Ar}=\sum_{k=1}^{n}t_{k}\times s_{k}^{T}e^{\lambda_{k}r}=t_{1}\times s_{1}^{T}+t_{2}\times s_{2}^{T}e^{-1}$, in which $t_{k}$ and $s_{k}$ are the left and right eigenvectors, corresponding to $\lambda_{k}$, respectively. Then, in our case $t_{1}={\left[1\;0\right]}^{T}$, $t_{2}={\left[1\;-r^{-1}\right]}^{T}$, $s_{1}={\left[1\;r\right]}^{T}$, $s_{2}={\left[0\;1\right]}^{T}$, so that $e^{Ar}=\left[\frac{1}{r}\;\;\;r+e^{-1};0\;\;\;-\frac{e^{-1}}{r}\right]$ is limited in norm (when r≠0) and it follows that $z_{1}\left(r\right)=z_{0,1}+\left(r+e^{-1}\right)z_{0,2}\;\;\;\wedge \;\;\;z_{2}\left(r\right)=\frac{z_{0,2}e^{-1}}{r}$ from which, eliminating *r*, we obtain $z_{2}=\frac{z_{0,2}^{2}e^{-1}}{z_{0,1}+z_{0,2}e^{-1}-z_{1}}$ which represents, on the plane $z_{1}z_{2}$, an equilateral hyperbola (see the red lines in Figure 6). Furthermore, we achieve $z_{2}\left(r\right)=\frac{z_{0,1}+rz_{0,2}}{r}-\frac{z_{1}\left(r\right)}{r}$, which represent straight lines passing through a fixed point (point A in Figure 6), as r→R. Then, as *r* increases (for r>0), the hyperbola is traversed such that the point *D* (see Figure 6 when r≠R) tends to the point *B* (when r=R).

If $\dot{z}=Az$ is stable, then the critical point of $\dot{u}\left(r\right)=f\left(u\left(r\right)\right)$ is also stable [5,30], and the critical point (60) is an equilibrium stable point for (62). □

Although $\left(u_{1}^{0},u_{2}^{0}\right)=\left(h-d^{*},0\right)$ identifies a point very close to the upper disk (with a high risk of the membrane touching it), it is still a stable point. Electrostatically, it can be justified as follows. Considering $\frac{1}{{\left(1-u\left(r\right)\right)}^{2}}\approx \frac{1}{d^{*}}$, one can get $p_{el}=\frac{1}{2}\frac{\epsilon_{0}V^{2}}{{\left(1-u\left(r\right)\right)}^{2}}\approx \frac{\epsilon_{0}V^{2}}{2d^{*}}$. Thereby fixing *V*, $p_{el}\approx \frac{\epsilon_{0}V^{2}}{2d^{*}}$, so that it does not swing.

### 10.3. Admissible Values for *V* in 2D Geometry

#### $V_{min}$ and the Problem to Win the Mechanical Inertia of the Membrane

Here, a condition to which ${\left(V_{min}\right)}_{inertia}$ must satisfy is presented in the following Proposition.

**Proposition** **13.** *If* (31) *holds, thus ${\left(V_{min}\right)}_{inertia}$ to win the membrane mechanical inertia satisfies:*

(67) $${\left(V_{min}\right)}_{inertia}>\sqrt[{4}]{\frac{h^{3}{d^{*}}^{2}T}{4\epsilon_{0}^{2}\theta k}}.$$

**Proof.**  From (31), it follows $\lambda^{2}>\frac{R^{2}{d^{*}}^{2}}{2\theta V^{2}\epsilon_{0}k}$, from which, considering (6), it follows that $V>\sqrt[{4}]{\frac{R^{2}{d^{*}}^{2}}{2k\epsilon_{0}\theta \lambda^{2}}}=\sqrt[{4}]{\frac{{d^{*}}^{2}d^{3}T}{4k\theta \epsilon_{0}^{2}}}$ obtaining (67). □

Both in 1D and 2D geometry ${\left(V_{min}\right)}_{inertia}$ are strongly dependent on *h*, *T* and θ. The dependence on *h* is imperative because the greater the distance between the plates, the greater the *V* to overcome the inertia of the membrane. Furthermore, the greater the *T*, the greater the mechanical tension of the membrane (i.e., the membrane is tighter), so that greater *V* is needed to overcome the inertia of the membrane. Finally, if θ is higher, it means that the influence of |E| on the device is greater so that a smaller value of *V* is sufficient to overcome the membrane inertia (see Remark 1).

### 10.4. ${\left(V_{max}\right)}_{permissible}$ in 2D Configuration

We present here some useful propositions [20].

**Proposition** **14.** *As in 1D geometry,* (44) *also holds. It is sufficient to replace x by r.*

**Remark** **12.** *Since the proof of Proposition 14 is the same as that given for 1D geometry, ${\left(V_{max}\right)}_{permissible}$ in 2D geometry is as for 1D geometry (see* (43)*).*

**Proposition** **15.** *For the 2D membrane MEMS device, $\Delta w\left(x\right)=-\frac{\lambda^{2}}{{\left(w\left(x\right)\right)}^{2}}$ holds. It is sufficient to replace L=0.5 with R.*

**Proposition** **16.** *For 2D geometry, the following inequality holds:*

(68) $$\sqrt[{4}]{\frac{h^{3}{d^{*}}^{2}T}{4\epsilon_{0}^{2}\theta k}}<\sqrt{\frac{2\left(h-d^{*}\right)d^{*}}{k\epsilon_{0}}}.$$

**Proof.**  If, absurdly,

(69) $$\sqrt[{4}]{\frac{d^{3}{d^{*}}^{2}T}{4\epsilon_{0}^{2}\theta k}}\geq \sqrt{\frac{2\left(h-d^{*}\right)d^{*}}{k\epsilon_{0}}},$$

considering $\Delta w\left(x\right)=-\frac{\lambda^{2}}{{\left(w\left(x\right)\right)}^{2}}$ and being $u_{0}\leq d-d^{*}$, it follows that $\frac{1}{u_{0}}\geq \frac{1}{d-d^{*}}$, easily achieving

(70) $$\frac{\sqrt{p_{el}}}{T}\leq \frac{d^{3}}{2R(h-d^{*})}\sqrt{\frac{d-d^{*}}{2\epsilon_{0}}}.$$

Being $\epsilon_{0}\approx 8.85\times 10^{-12}$, $R\approx 10^{-6}$, $d=10^{-9}$ and $d^{*}=0.1\times 10^{-9}$, from (70), we achieve $\frac{\sqrt{p_{el}}}{T}\leq 3.56\times 10^{-21}$. Thus, *T* should be too high a value, as if the membrane has considerable stiffness. This condition is not physically compatible with the usual membranes used in electrostatic MEMS devices. Thus, (69) is false, so that (68) is true. □

**Proposition** **17.** *In 2D geometry, the range of admissible values for V is:*

(71) $$\sqrt[{4}]{\frac{h^{3}{d^{*}}^{2}T}{4\epsilon_{0}^{2}\theta k}}<V<\sqrt{\frac{2\left(h-d^{*}\right)d^{*}}{k\epsilon_{0}}}.$$

**Proof.**  It follows from propositions (13) and (5). □

In 1D and 2D geometry, the range of admissible values for *V*, although different in the formulations (see (46) and (71), respectively), admit the same functional dependence with respect to *T*, θ and $d^{*}$. This ensures that the electromechanical properties of the membrane, the critical safety distance and mechanical stress to which the membrane is initially subjected affect the range of possible values for *V* in the same way for both geometries.

### 10.5. Relationship between ${\left(V_{min}\right)}_{inertia}$ and ${\left(V_{max}\right)}_{permissible}$ in 2D Geometry

**Proposition** **18.** *In this case, the following inequality holds:*

(72) $${\left(V_{min}\right)}_{inertia}>\sqrt[{4}]{\frac{d^{4}}{8(10-h)\theta }}\sqrt{{\left(V_{max}\right)}_{permissible}}\sqrt[{4}]{T}.$$

**Proof.**  From (43), and considering that $d^{*}\approx 0.1d$, it follows

(73) $$\frac{1}{k}>{\left(V_{max}\right)}_{permissible}{}^{2}\frac{50\epsilon_{0}}{d(10-d)}.$$

Furthermore, from (67), taking into account (73) and remembering that $d^{*}\approx 0.1d$, (72) is obtained. □

**Figure 7:**
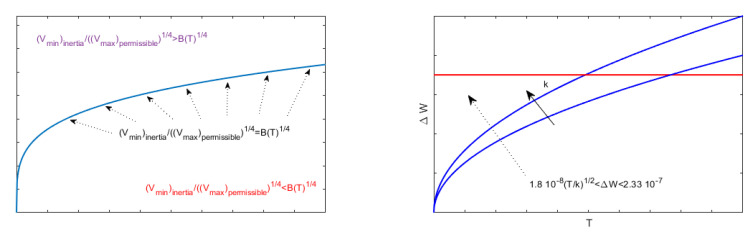
$\frac{{\left(V_{min}\right)}_{inertia}}{\sqrt{{\left(V_{max}\right)}_{permissible}}}$ versus $\sqrt[{4}]{T}$ (the blue separation line identifies two distinct areas of system behavior) and the zone of possible values for ΔW.

For the usual values $d=10^{-9}$, θ≈1, T=1000Pa, (72) becomes ${\left(V_{min}\right)}_{inertia}>3.3\times 10^{-6}\sqrt{{\left(V_{max}\right)}_{permissible}}$. Then, ${\left(V_{min}\right)}_{inertia}\ll {\left(V_{max}\right)}_{permissible}$, so that (72) makes physical sense. Moreover, (72) can be physically interpreted. Particularly in it, $V=\sqrt[{4}]{\frac{h^{4}}{8(10-h)\theta }}$ is constant. Thus, we can write $\frac{{\left(V_{min}\right)}_{inertia}}{\sqrt{{\left(V_{max}\right)}_{permissible}}}>B\sqrt[{4}]{T}$. Therefore, once *T* is fixed (i.e., the material constituting the membrane has been chosen), $\frac{{\left(V_{min}\right)}_{inertia}}{\sqrt{{\left(V_{max}\right)}_{permissible}}}$ depends on *B* changing with *d*. On the other hand, once *d* is fixed (i.e., once *B* has been chosen), the material constituting the membrane determines $\frac{{\left(V_{min}\right)}_{inertia}}{\sqrt{{\left(V_{max}\right)}_{permissible}}}$ (see Figure 7 left). We note that, in both geometries, the link between ${\left(V_{max}\right)}_{permissible}$ and ${\left(V_{min}\right)}_{inertia}$, in both cases, depends on *T* (even if with a different functional link). This confirms the fact that whatever the geometry, the larger the *T* is, the greater the ${\left(V_{min}\right)}_{inertia}$.

### 10.6. Some Optimal Control Conditions in 2D Geometry

If the membrane of the device is at rest, the distance between the membrane and the upper disk is *d*. Therefore, the electrostatic capacitance of the device is $C=\epsilon_{0}\frac{\pi R^{2}}{h}$, so that the potential energy of the device can be evaluated as $W_{initial}=\frac{1}{2}CV^{2}=\frac{\epsilon_{0}\pi R^{2}V^{2}}{2h}$. If the membrane deforms, $C=\epsilon_{0}\int_{0}^{\pi }Z\left(\phi \right)\int_{-R}^{R}\frac{dr}{h-u(r)}d\phi$, the final potential energy becomes $W_{final}=\frac{1}{2}\epsilon_{0}V^{2}\int_{0}^{\pi }Z\left(\phi \right)\int_{-R}^{R}\frac{dr}{h-u(r)}d\phi$, where Z(ϕ) is a bounded and continuous function depending on ϕ. Therefore, the total variation of the potential energy, $\Delta W=W_{final}-W_{initial}$ becomes

(74) $$\begin{array}{c} \Delta W=\epsilon_{0}V^{2}\left\{\frac{1}{2}\int_{0}^{2\pi }Z\left(\phi \right)\int_{-R}^{R}\frac{dr}{h-u(r)}d\phi -\frac{\pi R^{2}}{h}\right\}. \end{array}$$

Moreover, since $h-u\left(r\right)\geq h-d^{*}$, then $\frac{1}{h-u(r)}\leq \frac{1}{h-d^{*}}$, so that (74) becomes

(75) $$\Delta W\leq \epsilon_{0}V^{2}\left\{\frac{R}{h-d^{*}}\int_{0}^{2\pi }Z\left(\phi \right)d\phi -\frac{\pi R^{2}}{h}\right\}.$$

Z(ϕ), being a bounded and continuous function, allows to write $\int_{0}^{2\pi }Z\left(\phi \right)d\phi \leq D$, in which *D* is a positive constant. Then, (75) becomes

(76) $$\Delta W\leq \epsilon_{0}V^{2}\left\{\frac{RD}{h-d^{*}}-\frac{\pi R^{2}}{h}\right\}.$$

From (71), it follows that $\sqrt{\frac{h^{3}{d^{*}}^{2}T}{4\epsilon_{0}^{2}\theta k}}<V^{2}<\frac{2\left(h-d^{*}\right)d^{*}}{k\epsilon_{0}}$. Thus, considering (76), it follows

(77) $$\Delta W<\frac{2\left(h-d^{*}\right)d^{*}}{k}\left\{\frac{RD}{h-d^{*}}-\frac{\pi R^{2}}{h}\right\}.$$

Conversely, from (67), $V^{2}>{\left(V_{min}\right)}_{inertia}^{2}=\sqrt{\frac{h^{3}d^{*}T}{4\epsilon_{0}^{2}\theta k}}$ so that

(78) $$\Delta W>\epsilon_{0}\left\{\frac{RD}{h-d^{*}}-\frac{\pi R^{2}}{h}\right\}V^{2}>\sqrt{\frac{h^{3}d^{*}T}{4\epsilon_{0}^{2}\theta k}}.$$

Finally, combining (77) and (78), we achieve the range of the admissible values for ΔW, that is:

(79) $$\sqrt{\frac{h^{3}d^{*}}{4\epsilon_{0}^{2}}}\frac{\sqrt{T}}{\sqrt{k}\sqrt{\theta }}<\Delta W<\frac{2\left(h-d^{*}\right)d^{*}}{k}\left\{\frac{RD}{h-d^{*}}-\frac{\pi R^{2}}{h}\right\}$$

which can be written as

(80) $$\frac{F_{1}\sqrt{T}}{\sqrt{\theta }}<\Delta W<\frac{F_{2}}{k}$$

(where $F_{1}$ and $F_{2}$ constant) analogous to the (56) relating to 1D geometry. Figure 7right depicts the zone of possible values for ΔW, corresponding to the zone below the red straight line and above the blue curve. Obviously, this zone shrinks as *k* increases.

### 10.7. On the Values of *V* that Maximize ΔW

Since $u\left(r\right)\leq \frac{k\epsilon_{0}V^{2}}{2d^{*}}\left\{1-{\left(\frac{r}{R}\right)}^{2}\right\}$, $h-u\left(r\right)\geq h-\frac{k\epsilon_{0}V^{2}}{2d^{*}}\left\{1-{\left(\frac{r}{R}\right)}^{3}\right\}>\frac{2hd^{*}-k\epsilon_{0}V^{2}}{2d^{*}}$ so that

(81) $$\frac{1}{h-u(r)}<\frac{2d^{*}}{2hd^{*}-k\epsilon_{0}V^{2}}.$$

Being $C=\epsilon_{0}\int_{0}^{\pi }Z\left(\phi \right)\int_{-R}^{R}\frac{dr}{h-u(r)}d\phi$ and considering (81), one obtains $C<\frac{4\epsilon_{0}Dd^{*}R}{2hd^{*}-k\epsilon_{0}V^{2}}$. Thus,

(82) $$\Delta W=W_{final}-W_{initial}<\underset{h(V)}{\underset{︸}{\frac{2\epsilon_{0}Dd^{*}RV^{2}}{2hd^{*}-k\epsilon_{0}V^{2}}-\frac{\epsilon_{0}\pi R^{2}V^{2}}{2h}}}$$

where h(V) is positive, so that the unique stationary point for h(V) is $V^{*}=\sqrt{\frac{2hd^{*}\left(\pi R-4Dhd^{*}\right)}{\epsilon_{0}\pi kR}}$. Furthermore, $V^{*}$ is a point of maximum for h(V) (discarding the negative root which represents a non-physical condition to achieve). Furthermore, it is also easy to verify that

(83) $$V^{*}>{\left(V_{min}\right)}_{inertia}.$$

### 10.8. From |E|A to a Useful Limitation for ΔW

Starting from (9) and (24), we can write $\frac{\lambda^{2}}{{\left(1-u\left(r\right)\right)}^{2}}=\frac{1}{r}\frac{du(r)}{dr}+\frac{{\left(1-u\left(r\right)-d^{*}\right)}^{2}}{\theta \lambda^{2}}$. In addition, it follows that ${\left|E\right|}^{2}=\frac{1}{r\theta }\frac{du(r)}{dr}+\frac{{\left(1-u\left(r\right)-d^{*}\right)}^{2}}{\theta^{2}\lambda^{2}}$. However, $W_{final}=\frac{1}{2}\epsilon_{0}{\left|E\right|}^{2}$, it makes sense to write

(84) $$\Delta W=\frac{1}{2}\epsilon_{0}\left\{\frac{1}{r\theta }\frac{du(r)}{dr}+\frac{{\left(1-u\left(r\right)-d^{*}\right)}^{2}}{\theta^{2}\lambda^{2}}\right\}-\frac{\epsilon_{0}\pi R^{2}V^{2}}{2h}$$

and considering that $1-u-d^{*}<h-d^{*}$ and $\frac{du(r)}{dr}<\bar{H}$, with $\bar{H}=99$ positive constant (see [16]), (84) becomes

(85) $$\Delta W<\frac{1}{2}\epsilon_{0}\left\{\frac{1}{r\theta }\bar{H}+\frac{{\left(h-d^{*}\right)}^{2}}{\theta^{2}\lambda^{2}}\right\}-\frac{\epsilon_{0}\pi R^{2}V^{2}}{2h}.$$

**Proposition** **19.** *Considering both* (76) *and* (85)*, we can write*

(86) $$\epsilon_{0}V^{2}\left\{\frac{RD}{h-d^{*}}-\frac{\pi R^{2}}{h}\right\}<\frac{1}{2}\epsilon_{0}\left\{\frac{1}{r\theta }\bar{H}+\frac{{\left(h-d^{*}\right)}^{2}}{\theta^{2}\lambda^{2}}\right\}-\frac{\epsilon_{0}\pi R^{2}V^{2}}{2h}.$$

**Proof.**  Setting r=R, (86) becomes

(87) $$V^{2}\left\{\frac{RD}{h-d^{*}}-\frac{\pi R^{2}}{h}\right\}<\frac{1}{2}\left\{\frac{\bar{H}}{R\theta }+\frac{{\left(h-d^{*}\right)}^{2}}{\theta^{2}\lambda^{2}}\right\}-\frac{\pi R^{2}V^{2}}{2h}$$

so that, if (87) is true, (86) is also true. Moreover, (87) becomes

(88) $$RV^{2}\left\{\frac{D}{h-d^{*}}-\frac{\pi R}{2h}\right\}<\frac{1}{2}\left\{\frac{\bar{H}}{R}+\frac{{\left(h-d^{*}\right)}^{2}h^{3}T}{2\theta \epsilon_{0}R^{2}V^{2}}\right\}.$$

Finally, using the usual values for the parameters, (88) is verified so that (86) is also true. □

Then, by Proposition 19, it follows

(89) $$\Delta W<\frac{1}{2}\epsilon_{0}\left\{\frac{1}{r\theta }\bar{H}+\frac{{\left(h-d^{*}\right)}^{2}}{\theta^{2}\lambda^{2}}\right\}-\frac{\epsilon_{0}\pi R^{2}V^{2}}{2h},$$

which represents the limitation for ΔW (depending on θ), obtained from |E|.

The study of analytical models in 1D and 2D geometry [16,26] has determined algebraic conditions, ensuring the existence and uniqueness for the solution for both models. Then, by means of suitable numerical procedures, solutions are obtained that, if they satisfy the aforementioned algebraic conditions of existence and uniqueness, do not represent ghost solutions.

## 11. Numerical Approaches for Recovering of the Membrane Profile in 1D Geometry

### 11.1. Shooting Procedure and Ordinary Differential Equation Solvers

To apply the shooting procedure, in [17,19,22] (25) into the form (59) was considered to be turned into an initial value problem by replacing $u_{1}\left(0.5\right)$ at x=0.5 with $u_{2}\left(-0.5\right)=\eta$, η∈R. Therefore, by integration, one obtains $u_{1}\left(0.5\right)$ at x=0.5. If $u_{1}\left(0.5\right)=0$, one has solved the starting BVP defining a non-linear equation of the form $F\left(\eta \right)=u_{1}\left(0.5,\eta \right)=0$ iteratively solvable to achieve the correct value of η.

#### 11.1.1. Zeros of F(η): The Dekker–Brent Approach

This approach, used in [22], utilizes the bisection procedure to solve a non-linear equation. For each iteration, $b_{k}$ approximates temporary zero; $a_{k}$ represents the “contra-point” such that $F(a_{k})$ and $F(b_{k})$ have opposite signs so that $\left[a_{0},b_{0}\right]$ contains the solution; $b_{k-1}$ represents *b* at the previous iteration. Therefore, two temporary values are evaluated: the first is obtained by the secant method and the second by the bisection approach; $s=b_{k}-\frac{b_{k}-b_{k-1}}{F\left(b_{k}\right)-F\left(b_{k-1}\right)}F\left(b_{k}\right)$, if $F\left(b_{k}\right)\neq F\left(b_{k-1}\right)$, $s=m=\frac{a_{k}+b_{k}}{2}$ otherwise. If *s*, as result of the secant procedure, falls in $\left(b_{k},m\right)$, then $s=b_{k+1}$, otherwise $m=b_{k+1}$. Thus, the new value of the contra-point is chosen so that $F(a_{k+1})$ and $F(b_{k+1})$ have a different sign, so that $a_{k+1}=a_{k}$, otherwise, $a_{k+1}=b_{k}$. Finally, if $|F\left(a_{k+1}\right)|<|F\left(b_{k+1}\right)|$, $a_{k+1}$ is a best approximation of the solution, so that $a_{k+1}$ and $b_{k+1}$ are exchanged. Sometimes, $b_{k}$ converges slowly, so Brent proposes a modification of this approach using a test that must be satisfied before the result of the secant method is accepted for the next iteration [23]. If δ is a tolerance and if the previous step has been used in the bisection procedure, $\left|\delta |<\right|b_{k}-b_{k-1}|$ and $|\delta |<|s-b_{k}|<\frac{1}{2}\left|b_{k}-b_{k-1}\right|$ have to be applied, otherwise the bisection procedure is used again. If the previous step uses interpolation, $|\delta |<|b_{k-1}-b_{k-2}|$ and $|\delta |<|s-b_{k}|<\frac{1}{2}\left|b_{k-1}-b_{k-2}\right|$ are applied to decide if to perform the interpolation or the bisection. The Brent approach, ensuring that at the kth iteration the bisection method is used at most for $2\log_{2}\left(\frac{|b_{k-1}-b_{k-2}|}{\delta }\right)$ times, utilizes inverse quadratic interpolation instead of a linear one (as in the secant procedure).

#### 11.1.2. Obtaining the Solution

$\eta_{k}$, at each iteration, was evaluated in [17,19,22] by solving the related Initial Value Problem (IVP). Thus, a suitable stop criterion is used to verify if $\eta_{k}\to \eta$ as k→∞. The solutions are achieved exploiting both *ode23* and *ode45* MatLab^®^R2017a routines (accuracy and adaptivity parameters defined by default). However, the main difficulty in achieving the solutions is related to the integration of unstable initial value problems: thus, the solutions of the BVP could be insensitive from the variations of the boundary values. However, the solutions of the IVP achieved by the shooting procedure are computed by the variations of the initial values [22].

### 11.2. Relaxation Procedure and Keller–Box Scheme

To apply the relaxation procedure in [17,19,22,23], a mesh of points $x_{0}=-0.5$, $x_{j}=x_{0}+j\Delta x$, for j=1,2,⋯,J spaced with $x_{J}=L_{1}$ was exploited. The numerical solution $u(x_{j})$ was denoted by the $u_{j}$, j=0,1,⋯,J. The Keller–Box scheme [23] is writable as $u_{j}-u_{j-1}-\Delta F\left(x_{j-1/2}\frac{u_{j}+u_{j-1}}{2}\right)=0,\;\;\;j=1,\times ,J,$ with $G(u_{0},u_{J})=0$ and $x_{j-1/2}=\left(x_{j}+x_{j-1}\right)/2$. If u(x) and F(x,u) are sufficiently smooth, the solution is computable by the Newton procedure where $|\Delta y_{j\ell }|\leq \mathrm{TOL}$, $\Delta y_{j\ell }$, j=0,1,⋯,J and ℓ=1,2, is the difference between two successive iterate components (TOL is a fixed tolerance). $u_{1}\left(x\right)=1$, $u_{2}\left(x\right)=1$ are the initial guesses to start the iteration.

### 11.3. Collocation Procedure and III/IV-Stage Lobatto IIIa Formulas

#### 11.3.1. The Collocation Procedure

Starting from the following system of ordinary differential equations (ODEs)

(90) $$\frac{dy(r)}{dr}=F\left(r,u\left(r\right)\right)\;\;\;\;G\left[u\left(a\right),u\left(b\right)\right]=0$$

where G[u(a),u(b)]=0 represents the boundary conditions, one can write

(91) $$u\left(x\right)=u\left(x_{n}\right)+\int_{x_{n}}^{x}F\left(r,u\left(r\right)\right)dr$$

from which

(92) $$u\left(x\right)\approx u_{n}+\int_{x_{n}}^{x}p\left(r\right)dr,$$

where p(r) is an interpolation polynomial of degree lower than *s* interpoling $[x_{n,i},F\left(x_{n,i}\right),y\left(x_{n,i}\right)],\;\;\;i=1,2,\ldots ,s$, $x_{n,i}=x_{n}+\tau_{i}h,$i=1,…,s, $0\leq \tau_{1}<\ldots <\tau_{s}\leq 1$. If the Lagrange method is used, thus:

(93) $$p\left(r\right)=\sum_{j=1}^{s}F\left(x_{n,j},u\left(x_{n,j}\right)\right)L_{j}\left(r\right),$$

where $L_{j}\left(r\right)$ are the fundamental Lagrange polynomials. Therefore, plugging (93) into (92), one achieves

(94) $$u\left(x\right)\approx u_{n}+\sum_{j=1}^{s}F\left(x_{n,j},u\left(x_{n,j}\right)\right)\int_{x_{n}}^{x}L_{j}\left(r\right)dr$$

so that, (94) is forced for all the $x_{n,j}$, so that $u_{n,j}$ at collocation node points are obtained, for i=1,…,s, by

(95) $$u_{n,j}=u_{n}+\sum_{j=1}^{s}F\left(x_{x_{n,i}},u_{n_{n,j}}\right)\int_{x_{n}}^{x_{n,i}}L_{j}\left(r\right)dr.$$

If $\tau_{s}=1$, then $y_{n+1}=y_{n,s}$, otherwise $y_{n+1}=y_{n}+\sum_{j=1}^{s}F\left(x_{n,j},y_{n,j}\right)\int_{x_{n}}^{x_{n+1}}L_{j}\left(r\right)dr$. Collocation methods are reliable tools, although may not be suitable if high accuracy is required [23].

#### 11.3.2. Implicit Runge–Kutta Procedures

Runge–Kutta (RK) approaches require many evaluations of F(x,y(x)), $\forall [x_{n},x_{n+1}]$. Generally, an RK approach can be structured as [23] $u_{n+1}=u_{n}+h\sum_{i=1}^{s}b_{i}k_{i}$ where $k_{i}=F\left(x_{n}+c_{i}h,u_{n}+h\sum_{j=1}^{s}a_{ij}k_{j}\right)$, i=1,2,…,s and *s* denotes the number of stage of the procedure. Moreover, $\left\{a_{ij}\right\}$, {ci} and $\left\{b_{i}\right\}$ characterize an RK method and can be collected in the so-called Butcher Tableau [30,31]

(96) $$\begin{array}{cc} c & A\\ & b^{T} \end{array}$$

in which $A=\left(a_{ij}\right)\in R^{s\times s}$, $b={\left(b_{1},\ldots ,b_{s}\right)}^{T}\in R^{s}$ and $c={\left(c_{1},\ldots ,c_{s}\right)}^{T}\in R^{s}$. Moreover, if $a_{ij}=0$ for j≥i, with i=1,2,…,s, then each $k_{i}$ is evaluated using the i−1 coefficients $k_{1}\ldots k_{i-1}$ already computated. In this case, one has an RK implicit method. Otherwise (implict procedure) to compute $k_{i}$, one has to solve an *s*-dimensional non-linear system. To make an implicit RK procedure, the following three conditions must be considered [30]:

(97) $$B\left(p\right):\sum_{i=1}^{s}b_{i}c_{i}^{k-1}=k^{-1},\;\;\;k=1,2,\ldots ,p$$

(98) $$\begin{array}{c} C\left(q\right):\sum_{i=1}^{s}a_{ij}c_{i}^{k-1}=k^{-1}c_{i}^{k},\;\;\;k=1,2,\ldots ,p,\;\;i=1,2,\ldots ,s \end{array}$$

(99) $$\begin{array}{c} D\left(r\right):\sum_{i=1}^{s}b_{i}c_{i}^{k-1}a_{ij}=k^{-1}b_{j}\left(1-c_{j}^{k}\right),\;\;\;k=1,2,\ldots ,r,\;\;j=1,2,\ldots ,s. \end{array}$$

Particularly, (97) means that $\int_{x}^{x+h}F\left(s\right)ds\approx h\sum_{i=1}^{s}b_{i}F\left(x+c_{i}h\right)$ is exact for all polynomials in which the degree is lower than *p* (if (97) is satisfied, then the procedure has quadrature of order *q*). Analogously for condition (98): if it is satisfied, then $\int_{x}^{t+c_{i}h}F\left(s\right)ds\approx h\sum_{j=1}^{s}a_{ij}F\left(x+c_{j}h\right)$ are exact for all polynomials in which the degrees are lower than *q*. It is worth noting that all methods satisfying condition (98) having $c_{i}$, i=1,2,…,s distinct are collocation procedures [30].

#### 11.3.3. The Three-Stage Lobatto IIIa Formula

This procedure requires that $c_{i}$ be chosen as roots of [30] $P_{s}^{*}-P_{s-2}^{*}=\frac{d^{s-2}}{dx^{s-2}}\left(x^{s-1}{\left(x-1\right)}^{s-1}\right)$ (*s* is the number of the stage), achieving that $c_{1}=0$ and $c_{s}=1$∀s. Therefore, the quadrature formula is exact for any polynomial in which the degree is less than 2s−2 [32]. Two definitions are necessary for the following.

**Definition** **2.** *Let us define the mesh-grid:*

(100) $$0=a=r_{0}<r_{1}<\ldots <r_{n}=b=R$$

*defining, on it, the step-size $h_{m}=r_{m+1}-r_{m}$.*

**Definition** **3.** *$r_{m+1/2}$ is the midpoint of $\left(r_{m},r_{m-1}\right)$: Moreover, $u_{m+1/2}$ is the approximation of u(r) at $r_{m+1/2}$.*

**Remark** **13.** *p(r) satisfy the boundary conditions in* (90) *and, moreover, $\forall (r_{m},r_{m+1})$,* (100) *is considered. Furthermore, p(r) is located at the edges of each sub-interval and midpoint, in which p(r) is also continuous.*

This approach can be considered as a collocation procedure and it is equivalent to the three-stage Lobatto IIIa implicit RK procedure [32], the Butcher tableau of which is [30]
(101)
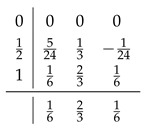

Thus, the three-stage Lobatto IIIa formula becomes [30]:

(102) $$\begin{array}{c} u_{m+1/2}=u_{m}+h_{m}\left[\frac{5}{24}F\left(r_{m},u_{m}\right)+\frac{1}{3}F\left(r_{m+1/2},u_{m+1/2}\right)-\frac{1}{24}F\left(r_{m+1},u_{m+1}\right)\right] \end{array}$$

and

(103) $$\begin{array}{c} u_{m+1}=u_{m}+h_{m}\left[\frac{1}{6}F\left(r_{m},u_{m}\right)+\frac{2}{3}F\left(r_{m+1/2},u_{m+1/2}\right)+\frac{1}{6}F\left(r_{m+1},u_{m+1}\right)\right]. \end{array}$$

**Remark** **14.** *Again, this procedure is achievable from* (91) *by the Simpson quadrature formula to approximate the integral between $x_{n}$ and x. Obviously, when the procedure is applied to a quadrature problem, it reduces* (103) *to the well-known Simpson formula [31]:*

(104) $$\begin{array}{c} u_{m+1}=u_{m}+\frac{h_{m}}{6}[F\left(r_{m},u_{m}\right)+F\left(r_{m+1},u_{m+1}\right)+4F\{r_{m+1/2},\frac{u_{m+1}+u_{m}}{2}+\\ +\frac{h_{m}}{8}[F\left(r_{m},F\left(r_{m},u_{m}\right)-F\left(r_{m+1},u_{m+1}\right)\right]\}]. \end{array}$$

**Remark** **15.** *p(r) with your derivatives satisfy, ∀r∈(a,b) [32] $p^{\left(l\right)}\left(r\right)=y^{\left(l\right)}\left(r\right)+O\left(h^{4-l}\right)$, l=0,1,2,3. Moreover,* (90) *is satisfied by p(r) at each intermediate point and at the midpoint of each interval (i.e., collocation polynomial). Furthermore, p(r) is chosen by MatLab^®^ by means of the determination of unknown parameters, if any. Finally*

(105) $$p^{\prime }\left(r_{m}\right)=F\left[r_{m},p\left(r_{m}\right)\right],\;\;\;p^{\prime }\left(r_{m+1/2}\right)=F\left[r_{m+1/2},p\left(r_{m+1/2}\right)\right],\;\;\;p^{\prime }\left(r_{m+1}\right)=F\left[r_{m+1},p\left(r_{m+1}\right)\right]$$

*which are non-linear equations solvable by a MatLab^®^ routine. In addition, MatLab^®^, ∀r∈(a,b), evaluates the cubic polynomial by means of its special routine bvpval [30].*

**Remark** **16.** *As known, a BVP could have more than one solution [31]. Thus, it is important to give an initial guess for both the initial mesh and the solution. Obviously, the MatLab^®^ solver makes the mesh adaptively achieve a solution using a reduced number of mesh points [30].*

However, a good initial hypothesis could be very difficult. Thus, the MatLab^®^ solver checks a residue defined as [30] $\mathrm{res}\left(r\right)=p^{\prime }\left(r\right)-F\left[r,p\left(r\right)\right]$. Obviously, if res(r) is small, then p(r) is a good solution. Moreover, the case of the well-conditioned problem, p(r) is next to y(r). In [23], MatLab^®^ R2017a *bvp4c* solver has been exploited since it implements the collocation technique by a piecewise cubic p(r), with coefficients determined requiring that p(r) be continuous on (a,b). Furthermore, both mesh and estimation error are based on the computation of the residual of p(r), the control of which is exploited to manage inadequate guesses for both mesh and solution [30]. Moreover, this routine provides a very reduced computational complexity to compute the Jacobian $J=\frac{\partial F_{i}}{\partial y}$. Finally, *bvp4c* is a vectorized solver, so that it is able to strongly reduce the run-time vectorizing F(r,y(r)) [31].

#### 11.3.4. Four-Stage Lobatto IIIa Formula

It is derived as an implicit RK method. Its Butcher tableau is [30]: (106)
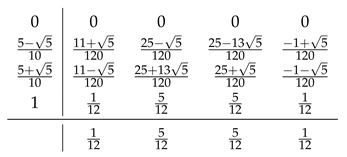

Like the three-stage formula, this approach is also a polynomial collocation procedure, but it provides solutions in which the accuracy is of the fifth-order, belonging to $C^{1}\left(\left[a,b\right]\right)$. However, unlike *bvp4c* that exploits the analytical condensation procedure, MatLab^®^ solves the four-stage Lobatto IIIA formula by a finite difference procedure (by means of *bvp5c* solver) and solves the algebraic equations directly. Furthermore, unlike *bvp4c* that handles the unknown parameters directly, *bvp5c* augments the system with trivial differential equations for unknown parameters [30,31].

### 11.4. Numerical Results

**Figure 8:**
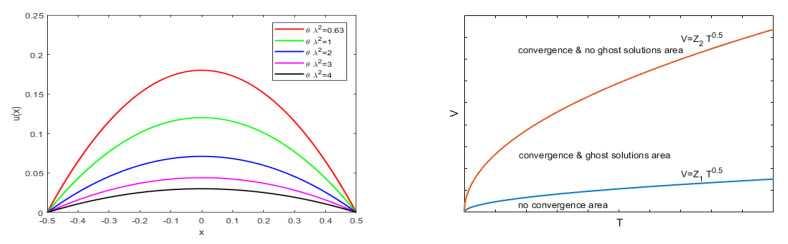
u(x) for different values of $\theta \lambda^{2}$ using the shooting procedure (MatLab ode23 routine), and T−V plane partitioned into three distinct areas: non-convergence area; convergence with ghost solutions area; convergence without ghost solutions area.

Figure 8 left depicts the numerical results for u(x) evaluated in [22] exploiting different values of $\theta \lambda^{2}$ using the shooting procedure implemented by the MatLab *ode23* routine. It can be noted that the minimum value of $\theta \lambda^{2}$ ensuring convergence is 0.63. Similar results have been achieved exploiting the other numerical procedures. For the shooting procedure and ODE solvers (indicated by Shoot&23 and Shoot&45), it was set $u_{1}\left(0\right)=1$ and $u_{2}\left(0\right)=1.2$ as initial guess when $\theta \lambda^{2}=0,63,1,4$ and $u_{1}\left(0\right)=0.1$ and $u_{2}\left(0\right)=0.2$ as initial guess when $\theta \lambda^{2}=2,3$. Regarding the relaxation procedure & Keller–Box scheme (Rel&Box), both initial guesses were set as $u_{1}\left(0\right)=u_{2}\left(0\right)=1$[22]. Finally, $u_{1}\left(0\right)=u_{2}\left(0\right)=0$ were set for the collocation procedure and Lobatto formulae (indicated by Col&III and Col&IV). Table 2 presents a comparison of the results achieved when $\theta \lambda^{2}=0.63,1,2,3,4$. Finally, when $\theta \lambda^{2}=4$, the same value max(u(x))=0.029918, with J=13, J=52, J=4000, J=4 number grid points, respectively, was achieved. The results proved that each procedure showed a good performance, even if different orders of accuracy and different grid points were used. Noting that both the relaxation method and Keller–Box scheme reveal robustness and accuracy, the latter provides results as accurate as those of the shooting and collocation method, since it involves more grid points for each iteration. However, although the shooting method is not so robust as the relaxation and collocation procedures, it is faster and well-implemented in MatLab. Moreover, the collocation procedure gives a solution by a few numbers of grid points because the profile is smooth. Finally, the Relaxation procedure and the Keller–Box scheme are more robust with respect to the other approaches.

### 11.5. Convergence of the Numerical Approaches

In [22], indicated by ${\left[{\left(\theta \lambda^{2}\right)}_{conv}\right]}_{S_{ode23}}$, the range of $\theta \lambda^{2}$ that guarantees convergence by the shooting method (using ode23 MatLab^®^) routine, it was obtained that ${\left[{\left(\theta \lambda^{2}\right)}_{conv}\right]}_{S_{ode23}}=\left[0.63,+\infty \right)$, so that, if ${\left[{\left(\theta \lambda^{2}\right)}_{no\;conv}\right]}_{S_{ode23}}=\left[0,0.63\right)$, the convergence is not guaranteed. Moreover, using the Keller–Box scheme, it was found that the range of $\theta \lambda^{2}$ ensuring convergence was ${\left[{\left(\theta \lambda^{2}\right)}_{conv}\right]}_{S_{Keller-Box}}=\left[0.592,+\infty \right)$. As mentioned above, of ${\left[{\left(\theta \lambda^{2}\right)}_{no\;conv}\right]}_{Keller-Box}=\left[0,0.592\right)$ the convergence of the Keller–Box scheme is not guaranteed. Moreover, the range of $\theta \lambda^{2}$ ensuring convergence when the shooting procedure was implemented by the ode45 MatLab^®^ routine was ${\left[{\left(\theta \lambda^{2}\right)}_{conv}\right]}_{S_{ode45}}=\left[0.63,+\infty \right)$, so that the range that did not guarantee convergence was ${\left[{\left(\theta \lambda^{2}\right)}_{no\;conv}\right]}_{S_{ode45}}=\left[0,0.63\right)$. Finally, exploiting both III and IV Lobatto IIIA formulas, in [22], it was found that ${\left[{\left(\theta \lambda^{2}\right)}_{conv}\right]}_{Three\;Stage_{Lobatto}}=\left[1.181,+\infty \right)$ and ${\left[{\left(\theta \lambda^{2}\right)}_{conv}\right]}_{Four\;Stage_{Lobatto}}=\left[1.181,+\infty \right)$. Then, for IV Stage Lobatto IIIa formulas, it was obtained that the range of $\theta \lambda^{2}$ that did not ensure convergence was ${\left[{\left(\theta \lambda^{2}\right)}_{no\;conv}\right]}_{Four\;Stage_{Lobatto}}=\left[0,1.181\right)$. These conditions are summarized in Table 3.

If all the procedures work parallelly, the minimum value of $\theta \lambda^{2}$ ensuring the convergence of at least one numerical procedure was obtained as follows [22]:

(107) $$\begin{array}{c} \min{\left(\theta \lambda^{2}\right)}_{conv}=\min\{\min{\left[{\left(\theta \lambda^{2}\right)}_{conv}\right]}_{S_{ode23}},\min{\left[{\left(\theta \lambda^{2}\right)}_{conv}\right]}_{S_{ode45}},\\ \min{\left[{\left(\theta \lambda^{2}\right)}_{conv}\right]}_{Keller-Box},\min{\left[{\left(\theta \lambda^{2}\right)}_{conv}\right]}_{ThreeStage_{Lobatto}},\min{\left[{\left(\theta \lambda^{2}\right)}_{conv}\right]}_{FourStage_{Lobatto}}\}=0.592. \end{array}$$

In other words, for values greater than 0.592, the convergence of at least one numerical solution is ensured. Then, for $\theta \lambda^{2}\geq 0.63$ convergence is ensured for all the numerical procedures considered. However, even if a numerical solution is obtainable, one must be sure that this solution does not represent a ghost solution.

### 11.6. Convergence and Ghost Solutions

As studied in [22], from (36), one obtains $\theta \lambda^{2}\geq 18$ so that if $\theta \lambda^{2}\in \left[18,+\infty \right)$ we have that both existence and uniqueness are ensured. Moreover, from (6) [16], we can write $\lambda^{2}=\frac{0.25\epsilon_{0}V^{2}}{h^{3}T}<\frac{0.25\epsilon_{0}V^{2}}{{\left(h-d^{*}\right)}^{3}T}$ from which

(108) $$\theta \lambda^{2}<\frac{0.25\theta^{2}}{{\left(h-d^{*}\right)}^{3}T}.$$

Moreover, combining (36) and (108), one obtains $1+{\left(\sup\left\{|\frac{du(x)}{dx}|\right\}\right)}^{6}<\frac{\theta \lambda^{2}}{18}\leq \frac{\theta \epsilon_{0}L_{1}^{2}V^{2}}{18{\left(h-d^{*}\right)}^{3}T}$, from which

(109) $$\begin{array}{c} 18({\left(\sup\left\{|\frac{du(x)}{dx}|\right\}\right)}^{6})\leq \theta \lambda^{2}\leq \frac{0.25\theta \epsilon_{0}}{{\left(h-d^{*}\right)}^{3}T}. \end{array}$$

Being $0.63\ll 18\left(1+{\left(\sup\left\{|\frac{dy(x)}{dx}|\right\}\right)}^{6}\right)$ we obtain $0.63\ll 18\left(1+{\left(\sup\left\{|\frac{dy(x)}{dx}|\right\}\right)}^{6}\right)<\theta \lambda^{2}\leq \frac{\theta \epsilon_{0}L_{1}^{2}V^{2}}{{\left(h-d^{*}\right)}^{3}T}$, so that

(110) $$V>\underset{Z1}{\underset{︸}{\sqrt{\frac{0.63{\left(h-d^{*}\right)}^{3}}{\theta \epsilon_{0}L_{1}^{2}}}}}\sqrt{T}=Z_{1}\sqrt{T}.$$

(110) highlights that the thicker the membrane, the higher *V* to be applied to the device for overcoming the inertia of the membrane. Moreover, since $18\ll 18\left(1+{\left(\sup\left\{|\frac{dy(x)}{dx}|\right\}\right)}^{6}\right)$, one can write:

(111) $$V>\underset{Z2}{\underset{︸}{\sqrt{\frac{18{\left(h-d^{*}\right)}^{3}}{\theta \epsilon_{0}L_{1}^{2}}}}}\sqrt{T}=Z_{2}\sqrt{T}.$$

Thus, both (110) and (111) identify, on the plane formed by T and V, zones of convergence where ghost solutions could also take place. As depicted in Figure 8 right [22], (110), below the blue curve, the non-convergence area is identified while, between the blue and red curves, at least one numerical procedure converges (ghost solutions could take place). Finally, above the red curve, the area in which convergence is guaranteed without ghost solutions is identified. To achieve solutions that are not ghost solutions is very important because it gives us the possibility to achieve ranges of possible values for *V*, |E| and *T* defining operating conditions to which the device has been subjected [17,19,22].

### 11.7. Range of Parameters for the Correct Use of the Device

In this section, we discuss the correct use of the device as studied in [22]. In other words, once the material constituting the membrane has been chosen (that is, *T* is fixed), what is the range of possible values for *V* and |E|? Vice versa, fixing both *V* and |E| (that is, fixing the intended use of the device), which material is more suitable for the membrane? Thus, starting from both (36) and (108), one obtains $1+{\left(\sup\left\{|\frac{du(x)}{dx}|\right\}\right)}^{6}<\frac{\theta \lambda^{2}}{18}=\frac{\theta }{18}\frac{0.25\epsilon_{0}V^{2}}{h^{3}T}<\frac{\theta }{18}\frac{0.25\epsilon_{0}V^{2}}{{\left(h-d^{*}\right)}^{3}T}$, so that $\left(\sup\left\{|\frac{du(x)}{dx}|\right\}\right)<\sqrt[{6}]{\frac{\theta }{18}\frac{0.25\epsilon_{0}V^{2}}{{\left(h-d^{*}\right)}^{3}T}-1}$ giving the interval of admissible values for $\sup\left\{|\frac{du(x)}{dx}|\right\}$, once θ, *T*and *V*are known. Moreover

(112) $$\begin{array}{c} {\theta |E|}^{2}=\frac{\lambda^{2}}{{\left(1-u\left(x\right)\right)}^{2}}=\frac{1}{{\left(1-u\left(x\right)\right)}^{2}}\frac{\epsilon_{0}V^{2}}{h^{3}T}<\frac{1}{{\left(1-u\left(x\right)\right)}^{2}}\frac{\epsilon_{0}V^{2}}{{\left(h-d^{*}\right)}^{3}T}. \end{array}$$

Multiplying (112) by $\lambda^{2}$ and considering that ${\left(1-u\left(x\right)\right)}^{2}<1$, ${\left|E\right|}^{2}<\sup\{{\left|E\right|}^{2}$, $\beta_{1}=\epsilon_{0}/2T$ and $\lambda^{2}=\beta_{1}V^{2}$, it is easy to write $\theta \lambda^{2}{\left|E\right|}^{2}=\frac{\beta_{1}V^{2}\lambda^{2}}{{1-u\left(x\right))}^{2}}=\frac{\beta_{1}^{2}V^{4}}{{\left(1-u\left(x\right)\right)}^{2}}$ from which

(113) $$\theta \lambda^{2}=\frac{\epsilon_{0}^{2}V^{4}}{T^{2}{\left(1-u\left(x\right)\right)}^{2}{\left|E\right|}^{2}}>\frac{\epsilon_{0}^{2}V^{4}}{T^{2}{\sup\{|E|}^{2}\}}$$

and

(114) $$T>\frac{\epsilon_{0}V^{2}}{\sqrt{\theta \lambda^{2}}\sup\{|E^{2}\left|\right\}}$$

or

(115) $$\frac{V^{2}}{\sup\{|E{|}^{2}\}}<\frac{T\sqrt{\theta \lambda^{2}}}{\epsilon_{0}}.$$

By (115), fixing θ and *T*, one obtains $\frac{V^{2}}{\sup\{|E^{2}|\}}$ (operative electrostatic parameters of the device). Vice versa, one obtains

(116) $$T\sqrt{\theta }>\frac{\epsilon_{0}V^{2}}{\sqrt{\lambda^{2}}{\sup\{|E|}^{2}\}}$$

so that, starting from |E| and *V*, *T* and θ are achieved.

## 12. Numerical Approaches for Recovering of the Membrane Profile in 2D Geometry

### 12.1. On the Applicability of the Numerical Procedure

As is known, BVPs are much more difficult to solve than IVPs [33]. Unlike the IVPs (having a unique solution), a BVP could not have a solution, could have a finite number or could have infinitely many. To solve BVPs in 2D geometry, shooting procedures and a collocation one can be exploited. The first one combines a numerical procedure based on the solution of a corresponding IVP for ordinary differential equations and one for the solution of non-linear algebraic equations. However, the shooting procedure could require the integration of an unstable IVP, so that the solution of a BVP could be insensible to the changes in the boundary value and the solution of the IVP could be sensible to the changes in the initial values. On the other hand, the collocation procedure, even if they are efficient and reliable tools, often could not be suitable for high accuracy. In [22,23], the authors used the collocation procedure based on piecewise polynomial functions for solving (24) because just one coefficient was singular and, moreover, the solutions were smooth [33]. Thus, they proposed the collocation procedure implemented in MatLab^®^, by its routine bvp4c, because its code implements the three-stage Lobatto IIIa formula, which is a collocation formula exploiting a collocation polynomial, which provides a $C^{1}$ continuous solution that is fourth-order accurate.

### 12.2. Numerical Procedure and Convergence: Interesting Ranges of $\theta \lambda^{2}$ and $V^{2}k$

#### 12.2.1. $\theta \lambda^{2}$ and Its Characteristics Ranges

As specified in [23], instability phenomena of the membrane can arise when *V* grows too much. Thus, it is important to know the range of *V* generating instability. With *V* being linked to $\theta \lambda^{2}$ (see (31)), it follows that the knowledge of the behavior of the membrane when $\theta \lambda^{2}$ increases gives us, when both $d^{*}$ and *k* are fixed, the range of *V* producing instability of the membrane in absence/presence of ghost solutions. In [23] it has been observed that $\theta \lambda^{2}$, depending on both the electromechanical properties of the material constituting the membrane and *V*, once the ranges of stability/instability of the membrane are known, it is possible to know the operation parameters in the convergence area respecting (31) and the engineering areas of applicability of the device. In such a context, it was reasonable to consider that $p_{el}$ and *p* are equivalent (i.e., k=1 and negligible losses). This is correct because when *V* is applied |E| and $p_{el}$ are generated inside the device. In [23] all the simulations have been carried out by the bvp4c MatLab^®^ solver exploiting both the default relative and absolute error tolerances obtaining 100 as the optimal number of grid points on [0,R]=[0,1] (by a greater number of grid points the performance did not improve). Moreover, different solutions starting with different initial guesses were obtained. Three cases occurred.

Case 1

**Figure 9:**
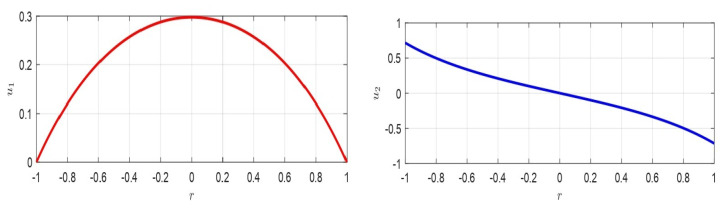
Recovering of the membrane: $\theta \lambda^{2}=0.5$, $u_{1}\leq 2.446$, $u_{2}=0$.

**Figure 10:**
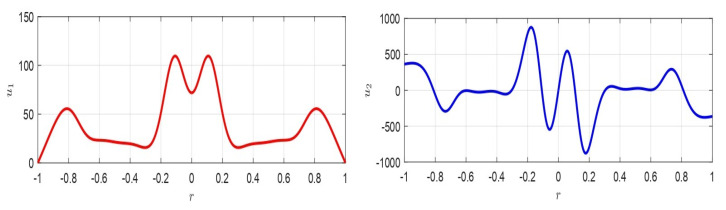
Recovering of the membrane: $\theta \lambda^{2}=0.5$, $2.447\leq u_{1}\leq 2.453$, $u_{2}=0$.

**Figure 11:**
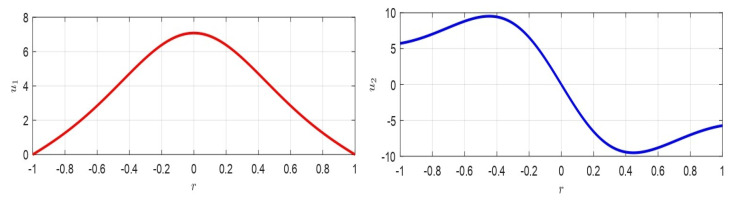
Recovering of the membrane: $\theta \lambda^{2}=0.5$, $2.454\leq u_{1}\leq 9.474$, $9.63\leq u_{1}\leq 12.7$ and $15.1\leq u_{1}\leq 19.978$, $u_{2}=0$.

**Figure 12:**
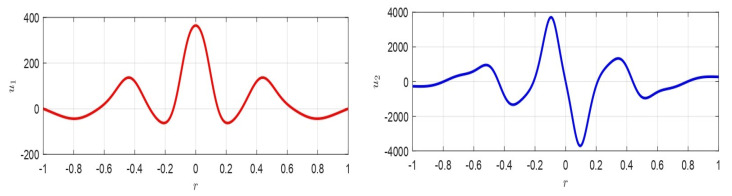
Recovering of the membrane: $\theta \lambda^{2}=0.5$, $9.475\leq u_{1}\leq 9.62$, $12.71\leq u_{1}\leq 15$ and $u_{1}\geq 19.979$, $u_{2}=0$.

$\forall \theta \lambda^{2}\in \left(10^{-6},+\infty \right)$ the numerical procedure converged without instabilities. In other words, when $\theta \lambda^{2}$ increased from $10^{-6}$, $\frac{d^{2}\left(u\right)}{dr^{2}}$ increased from negative values towards zero. Thus, the concavity of the deformed membrane decreased avoiding instabilities next to the edge. This was confirmed by (31): the higher $\theta \lambda^{2}$ is, the lower $V^{2}$ will be (increasing $\theta \lambda^{2}$, the membrane does not deform too much when a low *V* is applied). Figure 9, as achieved in [23], displays an example of recovering with $\theta \lambda^{2}=0.5$, and initial guesses $u_{1}\leq 2.446$ and $u_{2}=0$. With *V* being reduced, the membrane moves just a little so that instabilities do not appear. The procedure behaves differently when the initial guess of $u_{1}$ increases (with $u_{2}=0$). In fact, Figure 10, Figure 11 and Figure 12 depict examples of recovering when $\theta \lambda^{2}=0.5$ and with the initial guess for $u_{1}$ belonging to [2.447,2.453], [2.454,9.474], [9.63,12.7] and [15.1,19.978], [9.475,9.62], [12.71,15] and [19.979,+∞), respectively (initial guess for $u_{2}$ is zero).

One can note that when the initial guess for $u_{1}$ and $u_{2}=0$ increases, the recovering of the membrane is symmetrical but erratic ( Figure 10) until the profile assumes a bell-shape (Figure 11). The erratic behavior is also present when the initial guess is increasing (Figure 12). However, although Figure 10, Figure 11 and Figure 12 show simulations that numerically are valid, since $u_{1}$ is greater than *d*, they are not realistic.

Case 2

If $\theta \lambda^{2}\in \left(0,10^{-7}\right)$, the numerical procedure does not work: $\frac{{\left(1-u\left(r\right)-d^{*}\right)}^{2}}{\theta \lambda^{2}}$ increases too much, (as $\theta \lambda^{2}\to 0$, $\frac{{\left(1-u\left(r\right)-d^{*}\right)}^{2}}{\theta \lambda^{2}}\to \infty$). Thus, the procedure stops since the Jacobian matrix is singular.

Case 3

**Figure 13:**
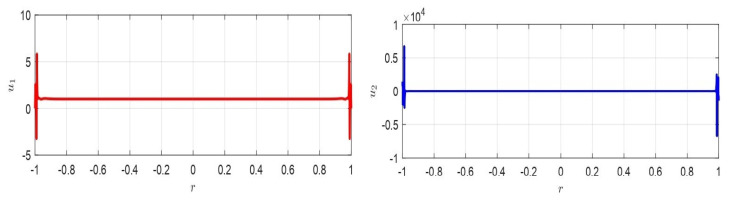
Recovering of the membrane: $u_{1}=0.1$ and $\theta \lambda^{2}=5\times 10^{-7}$.

**Figure 14:**
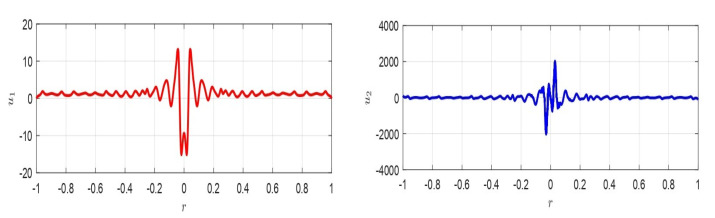
Recovering of the membrane: $u_{1}=1.2$ and $\theta \lambda^{2}=5\times 10^{-5}$.

If $\theta \lambda^{2}\in \left[10^{-7},10^{-6}\right]$ strong instabilities occur. Thus, although the numerical method does not stop, instabilities close to the edge of the membrane occurred. Figure 13 and Figure 14 depicts two examples of recovering when $\theta \lambda^{2}\in \left[10^{-7},10^{-6}\right]$ highlighting instabilities. Particularly, Figure 13 has been achieved in [23] setting $u_{1}=0.1$ and $\theta \lambda^{2}=5\times 10^{-7}$, while Figure 14 has been achieved when $u_{1}=1.2$ and $\theta \lambda^{2}=10^{-6}$.

#### 12.2.2. $\theta \lambda^{2}$ and Analytical Condition of Uniqueness of the Solution

From (31), being $d^{*}=10^{-9}$, k=1 and $\epsilon_{0}\approx 10^{-12}$, then $\theta \lambda^{2}>\frac{R^{2}{d^{*}}^{2}}{2V^{2}\epsilon_{0}k}\approx \frac{{\left(10^{-6}\right)}^{2}{\left(10^{-9}\right)}^{2}}{V^{2}10^{-12}k}=\frac{10^{-18}}{V^{2}k}$ from which [23]:

(117) $$\theta \lambda^{2}>\frac{10^{-18}}{V^{2}k}.$$

Considering (117), with $\theta \lambda^{2}\leq 10^{-6}$ meaning the numerical procedure stably converges, one can write $\theta \lambda^{2}>\frac{10^{-18}}{V^{2}k}$∧$\theta \lambda^{2}\geq 10^{-6}$ from which $V^{2}k\leq 10^{-12}$, so that the range of $V^{2}k$ ensuring both convergence and stability is $V^{2}k\in \left(0,10^{-12}\right]$. As seen above, $\forall \theta \lambda^{2}\in \left(0,10^{-7}\right)$ the numerical procedure does not converge obtaining $\theta \lambda^{2}>\frac{10^{-18}}{V^{2}k}$∧$\theta \lambda^{2}\leq 10^{-7}$ from which $V^{2}k\geq 10^{-11}$; so that, $\forall V^{2}k\in \left[10^{-11},+\infty \right)$ the numerical procedure does not converge. If the numerical procedure unstably converges, then $\theta \lambda^{2}\in \left[10^{-7},10^{-6}\right]$. Thus, $\theta \lambda^{2}>\frac{10^{-18}}{V^{2}k}$∧$10^{-7}<\theta \lambda^{2}<10^{-6}$ from which $\forall V^{2}k\in \left(10^{-12},10^{-11}\right)$. Here, the numerical procedure converges even if instability phenomena can occur close to the edges.

### 12.3. An Overview on the Ghost Solutions Areas

As shown in [23], one starts from $\theta \lambda^{2}>\frac{10^{-18}}{V^{2}k}$. Moreover, being $u_{0}=\frac{k\epsilon_{0}V^{2}}{{\left(h-u\left(r\right)\right)}^{2}}$ [20]. Then

(118) $$V^{2}k=\frac{2u_{0}{\left(h-u\left(r\right)\right)}^{2}}{\epsilon_{0}}$$

which, combining with $\theta \lambda^{2}>\frac{10^{-18}}{V^{2}k}$, becomes

(119) $$\theta \lambda^{2}>\frac{10^{-18}\epsilon_{0}}{2\;u_{0}{\left(h-u\left(r\right)\right)}^{2}}.$$

Considering that $\epsilon_{0}\approx 10^{-12}$, $u_{0}\approx 10^{-9}$ and ${\left(h-u\left(r\right)\right)}^{2}\approx 10^{-9}$, from (119), it is easy to obtain:

(120) $$\theta \lambda^{2}>\frac{10^{-18}\epsilon_{0}}{2u_{0}{\left(h-u\left(r\right)\right)}^{2}}\approx \frac{10^{-18}10^{-12}}{2\;10^{-9}10^{-9}}\approx 10^{-12}.$$

Then, it follows that (119) becomes $\theta \lambda^{2}>10^{-12}$. Therefore, $\forall \theta \lambda^{2}\in \left(0,10^{-12}\right]$ any solutions could be ghost solutions. However, since $\forall \theta \lambda^{2}\in \left(0,10^{-7}\right]$ the numerical procedure does not converge, then each numerical solution obtained is not a ghost solution. The results are summarized in Table 4.

### 12.4. Electromechanical Properties of the Membrane, *V* and Ghost Solutions: Exploitation of the Device

As above discussed, θ represents the proportionality between |E| and $\frac{\lambda^{2}}{{\left(1-u\left(r\right)\right)}^{2}}$ [17]:

(121) $${\theta |E|}^{2}=\frac{\lambda^{2}}{{\left(h-u\left(r\right)\right)}^{2}}.$$

Moreover, $\lambda^{2}$ depends on both *V* and the electromechanical properties of the material constituting the membrane (see (6) and (7)). Then, combining (121) with (6) one achieves [23]:

(122) $${\theta |E|}^{2}=\frac{\lambda^{2}}{{\left(h-u\left(r\right)\right)}^{2}}=\frac{\epsilon_{0}V^{2}{\left(2R\right)}^{2}}{2h^{3}T{\left(h-u\left(r\right)\right)}^{2}}=\frac{\rho V^{2}}{{\left(h-u\left(r\right)\right)}^{2}}.$$

Again ${\theta |E|}^{2}\lambda^{2}=\frac{\epsilon_{0}{\left(2R\right)}^{2}}{2h^{3}T}\frac{V^{2}\lambda^{2}}{{\left(h-u\left(r\right)\right)}^{2}}=\rho \frac{V^{2}\lambda^{2}}{{\left(h-u\left(r\right)\right)}^{2}}=\rho^{2}\frac{V^{4}}{{\left(h-u\left(r\right)\right)}^{2}}$ obtaining

(123) $$\theta \lambda^{2}=\frac{4\epsilon_{0}^{2}R^{4}}{h^{6}T^{2}}\frac{V^{4}}{{\left(h-u\left(r\right)\right)}^{2}{\left|E\right|}^{2}}.$$

However, in dimensionless conditions, ${\left(h-u\left(r\right)\right)}^{2}<1$, d=R=1 and ${\left|E\right|}^{2}{<\sup\{|E|}^{2}\}$; thus, from (123), one can write $\theta \lambda^{2}=\frac{4\epsilon_{0}^{2}}{T^{2}}\frac{V^{4}}{{\left(h-u\left(r\right)\right)}^{2}{\left|E\right|}^{2}}>\frac{\epsilon_{0}^{2}V^{4}}{4T^{2}{\sup\{|E|}^{2}\}}$. Moreover, as known, $\theta \lambda^{2}$ is writable as [17,20] $\theta \lambda^{2}=\frac{\epsilon_{0}V^{4}}{4T^{2}{\left(h-u\left(r\right)\right)}^{2}{\left|E\right|}^{2}}$ from which $\theta \lambda^{2}=\frac{\epsilon_{0}^{2}V^{4}}{T^{2}{\left(h-u\left(r\right)\right)}^{2}{\left|E\right|}^{2}}>\frac{\epsilon_{0}^{2}V^{4}}{T^{2}{\sup\{|E|}^{2}\}}$. If the numerical procedure does not converge, it follows that $10^{-7}>\theta \lambda^{2}>\frac{\epsilon_{0}^{2}V^{4}}{T^{2}{\sup\{|E|}^{2}\}}$ so that:

(124) $$\frac{\epsilon_{0}^{2}V^{4}}{T^{2}{\sup\{|E|}^{2}\}}<10^{-7}.$$

Then, from (124), one obtains:

(125) $$T>\frac{\epsilon_{0}V^{2}}{\sqrt{10^{-7}{\sup\{|E|}^{2}\}}}$$

or

(126) $$\frac{V^{4}}{{\left[\sup\{|E\right|}^{2}{\}]}^{2}}<\frac{T^{2}10^{-7}}{\epsilon_{0}^{2}}.$$

Once the intended use of the device has been chosen (that is, once the pair ${\left\{V,\sup\{|E\right|}^{2}\}$ has been fixed), from the inequality (125), inf{T} is computable. In other words, once ${\left\{V,\sup\{|E\right|}^{2}\}$ has been chosen, the material of the membrane is selected. Conversely, if the material of the membrane has been chosen (that is, *T* has been fixed), it is possible to achieve the pair ${\left\{V,\sup\{|E\right|}^{2}\}$ satisfying the inequality (126) (that is the intended use of the device is achieved).

## 13. Electromechanical Properties of the Membrane and Exploitation of the Device: A Useful Comparison between the 1D and 2D Formulations in Convergence Conditions

The following result holds.

**Proposition** **20.** *In convergence conditions, considering both* (114) *and* (125)*, we can write:*

(127) $$\frac{\epsilon_{0}V^{2}}{\sqrt{10^{-7}{\sup\{|E|}^{2}\}}}>\frac{\epsilon_{0}V^{2}}{\sqrt{\theta \lambda^{2}}{\sup\{|E|}^{2}\}}.$$

**Proof.**  In convergence conditiond, $\frac{\epsilon_{0}V^{2}}{\sqrt{\theta \lambda^{2}}\sup\{|E^{2}\left|\right\}}$ in (114) becomes $\frac{\epsilon_{0}V^{2}}{\sqrt{0.63}\sup\{|E^{2}\left|\right\}}$, so that $\frac{1}{\sqrt{10^{-7}}}>\frac{1}{\sqrt{0.63}}$. Therefore, (127) follows. □

From the Proposition 20 it follows that, with the same *V* and $\sup\{|E{|}^{2}\}$ (i.e., fixed the intended use of the product), the machine voltage *T* is higher in 2D geometry. This is due to the fact that, in 2D geometry, it is necessary to take into account all the contributions due to the mechanical stresses relative to each vertical plane passing through the vertical axis of symmetry. Recall that each of these planes is affected by 1D geometry.

## 14. Conclusion and Perspectives

In recent literature, the surveys published on MEMS membrane electrostatic devices are abundant and many of them are of high quality. The discussions contained therein range from the design procedures of the individual devices to the techniques for analyzing the behavior of the devices under the most varied operating conditions. However, recently, a new line of research has emerged on MEMS membrane devices based on the observation that on each point of the membrane E is always orthogonal to the tangent to the membrane at the point in question, so that |E| is to be considered proportional to the curvature *K* of the membrane. This approach allows modeling the problem by means of elliptic semi-linear second-order differential models relative to 1D and 2D geometries (the latter with circular symmetry). Particularly for the 1D geometry, the explicit singularities present in models known in the literature are not present, while for the 2D geometry, the singularity is present on the axis of symmetry passing through the center of the circular plates of the device. In this survey, in addition to having presented and discussed both the differential models mentioned above, the main results of existence and uniqueness of the solution for both models were presented, discussed and compared in the first part, verifying which conditions are stronger than the remaining. The second part of the survey was entirely devoted to stability and optimal control problems for both geometries, underlining the similarities and differences that emerge from the study of both geometries. In particular, important conditions that manage the range of possible values for *V* have been studied and compared providing original results as far as the 2D geometry is concerned. The last part of this survey, since the analytical models do not provide explicit solutions, was entirely dedicated to the comparison of the results obtained from the numerical procedures applied to reconstruct the membrane profile. Particularly, respecting the analytical conditions of existence and uniqueness of the solution and for both geometries studied, the ranges of admissible values for *T* to which the membrane must be subjected before deformation under the effect of *V* were compared. The comparisons highlighted how both geometries provide limitations and a range of possible values for the fundamental quantities (such as, for example, *V* and *T*) dependent on the same parameters but with different functional bonds due to the different membrane shapes in the two geometries. This, at least qualitatively, confirms that both models studied have been correctly formulated, highlighting a certain uniformity of behavior of the control parameters. Finally, we observe that, even if the approach of considering |E| is proportional to *K*, as validated by the results obtained in the absence of ghost solutions, it appears clear that the formulations used for *K* require refinement; therefore, in the future, it would be desirable to exploit more sophisticated formulations for membrane curvature in order to take into account the symmetries present in the geometries studied. Moreover, by removing the hypothesis that $u\left(x\right)\in C^{2}\left(\bar{\Omega }\right)$, new scenarios open up regarding the reformulation of the curvature, thus taking into account any formation of wrinkles around the membrane. It is worth underlining the fact that the numerical models used enjoy a limited computational load. Then, the writing of the hardware of what has been implemented in the software appears desirable in order to make the elaboration usable for any real-time applications.

## Figures and Tables

**Table 1:** Algebraic conditions ensuring the existence and uniqueness of the solution for both geometries.

	1D Geometry	2D Geometry
Existence	$1+H^{6}<\frac{H\theta {\bar{\lambda }}^{2}}{2(h-d^{*})}$	$\theta \lambda^{2}>\frac{d^{*2}}{2V^{2}\epsilon_{0}k}$
Uniqueness	$1+H^{6}<\frac{\theta \lambda^{2}}{18}$	not ensured
Existence and Uniqueness	$1+H^{6}<\frac{\theta \lambda^{2}}{18}$	$\theta \lambda^{2}>\frac{d^{*2}}{2V^{2}\epsilon_{0}k}$

**Table 2:** Comparison of the results for different values of the parameter $\theta \lambda^{2}$.

	$\theta \lambda^{2}=0.63$		$\theta \lambda^{2}=1$	
**Methods**	max(y(x))	J	max(y(x))	J
Shoot&23	0.190943	64	0.113476	20
Shoot&45	0.189749	364	0.113639	56
Rel&Box	0.191257	4000	0.113662	4000
Col&III	0.189623	44	0.113653	12
Col&IV	0.190331	40	0.113662	10
	$\theta \lambda^{2}=2$		$\theta \lambda^{2}=3$	
**Methods**	max(y(x))	J	max(y(x))	J
Shoot&23	0.057973	14	0.039371	14
Shoot&45	0.058133	52	0.039432	52
Rel&Box	0.058133	4000	0.039452	4000
Col&III	0.058124	4	0.039451	4
Col&IV	0.058133	6	0.039452	4

**Table 3:** Range of $\theta \lambda^{2}$ ensuring convergence for each numerical procedure.

**Shooting**	$\theta \lambda^{2}\in \left[0.63,+\infty \right)$	$\theta \lambda^{2}\in \left[0,0.63\right)$
***ode*23**	convergence	no convergence
**Shooting**	$\theta \lambda^{2}\in \left[0.63,+\infty \right)$	$\theta \lambda^{2}\in \left[0,0.63\right)$
***ode*45**	convergence	no convergence
**Keller-Box**	$\theta \lambda^{2}\in \left[0.592,+\infty \right)$	$\theta \lambda^{2}\in \left[0,0.592\right)$
	convergence	no convergence
**Three Stage**	$\theta \lambda^{2}\in \left[1.181,+\infty \right)$	$\theta \lambda^{2}\in \left[0,1.181\right)$
**Lobatto IIIa** **(*bvp4c*)**	convergence	no convergence
**Four Stage**	$\theta \lambda^{2}\in \left[1.181,+\infty \right)$	$\theta \lambda^{2}\in \left[0,1.181\right)$
**Lobatto IIIa** **(*bvp5c*)**	convergence	no convergence

**Table 4:** Convergence and stability areas.

No Convergence	Convergence and Instability	Convergence and Stability
$\theta \lambda^{2}\leq 10^{-7}$	$10^{-7}<\theta \lambda^{2}<10^{-6}$	$\theta \lambda^{2}\geq 10^{-6}$
$V^{2}k\geq 10^{-11}$	$10^{-12}<V^{2}k<10^{-11}$	$V^{2}k\leq 10^{-12}$
No Ghost Solutions if $\theta \lambda^{2}>10^{-12}$	No Ghost Solutions	No Ghost Solutions

## Abbreviations

The following abbreviations are used in this manuscript:

MEMS	Micro-Electro-Mechanical-System
*V*	applied voltage
*u*	deflection of the membrane
E	electrostatic field
|E|	amplitude of the electrostatic field
*K*	curvature of the membrane
Ω	boundary region
ρ, γ, χ	parameters related to the electric and mechanic properties of the membrane
σ	Coulombian exponent
ϕ	electrostatic potential
*D*	flexural stiffness
*T*	mechanical tension of the membrane
$\epsilon_{0}$	permittivity of the free space
h£	distance between the two parallel plates
δ	rigidity of the plate
ϵ	aspect ratio of the system
$d^{*}$	critical security distance
$p_{el}$	electrostatic pressure
*p*	mechanical pressure
*k*	coefficient of proportionality between $p_{el}$ and *p*
θ	parameter of proportionality for |E|
μ	function of proportionality between *K* and |E|
$\epsilon_{t}$	dielectric strength of the material constituting the membrane
*R*	radius of the disks in 2D geometry
*r*	radial coordinate
$C_{el}$	electrostatic capacintance
H(r)	mean curvature
*G*	Green function
*H*	$\sup|\frac{du(x)}{dx}|$
BVP	boundary value problem
IVP	initial value problem
RK	Runge–Kutta approach

## Appendix A. Mean Curvature and 2D Modellization

Here, a surface *S* generated by rotating around *z* a curve *C* lying on a vertical plane normal to the xy plane, which forms an angle *t* with the zx plane, is considered (Figure 3a) [29]. To simplify the procedure, let us suppose that *C* is parametrized with *r* (generic parameter). Thus, $P\left(r\right)=(\bar{f}\left(r\right),0,\bar{g}\left(r\right))$, r∈I⊂R, in which $\bar{f}\left(r\right)$ and $\bar{g}\left(r\right)$ are regular functions, which satisfy the following inequality

(A1) $${\left(\frac{d\bar{f}\left(r\right)}{dr}\right)}^{2}+{\left(\frac{d\bar{g}\left(r\right)}{dr}\right)}^{2}\geq 0$$

∀r∈I=[0,R]. *S* is parametrizable as $P\left(t,r\right)=(\bar{f}\left(r\right)\cos t,\bar{f}\left(r\right)\sin t,\bar{g}\left(r\right))$ where (t,r)∈[0,2π)×I.

**Remark** **A1.**
*P(r), being a so-called natural parametrization, ensures that the curve C is regular everywhere. Thus, by rotation, S is also regular.*

Thus, one can easily achieve

(A2) $$\frac{\partial P(t,r)}{\partial t}=\left(-\bar{f}\left(r\right)\sin t,\bar{f}\left(r\right)\cos t,0\right)\;\;\;\;\wedge \;\;\;\;\frac{\partial P(t,r)}{\partial r}=\left(\frac{d\bar{f}\left(r\right)}{dr}\cos t,\frac{d\bar{f}\left(r\right)}{dr}\sin t,\frac{d\bar{g}\left(r\right)}{dr}\right)$$

from which, the coefficients of the first fundamental form become:

(A3) $$E=∥\frac{\partial P(t,r)}{\partial r}{∥}^{2}={\bar{f}}^{2}\left(r\right);\;\;\;\;F=\frac{\partial P(t,r)}{\partial r}\times \frac{\partial P(t,r)}{\partial t}=0;\;\;\;G=∥\frac{\partial P(t,r)}{\partial t}{∥}^{2}=1.$$

Observing that F=0 everywhere, thus the coordinate lines are orthogonal to each other (everywhere), so that, one can write:

(A4) $$\begin{array}{c} \frac{\partial^{2}P\left(t,r\right)}{\partial t^{2}}=\left(-\bar{f}\left(r\right)\cos t,-\bar{f}\left(r\right)\sin t,0\right),\;\;\;\frac{\partial^{2}P\left(t,r\right)}{\partial t\partial r}=\left(-\frac{d\bar{f}\left(r\right)}{dr}\sin t,\frac{d\bar{f}\left(r\right)}{dr}\cos t,0\right),\\ \frac{\partial^{2}P\left(t,r\right)}{\partial r^{2}}=\left(\frac{\partial \bar{f}\left(r\right)}{\partial r^{2}}\cos t,\frac{\partial^{2}\bar{f}\left(r\right)}{\partial r^{2}}\sin t,\frac{\partial^{2}\bar{g}\left(r\right)}{\partial r^{2}}\right). \end{array}$$

Therefore, from (A2), $\frac{\partial P(t,r)}{\partial t}\wedge \frac{\partial P(t,r)}{\partial r}=\bar{f}\left(r\right)\left(\frac{dg(r)}{dr}\cos t,\frac{dg(r)}{dr}\sin t,-\frac{d\bar{f}\left(r\right)}{dr}\right)$, so that the unit normal vector to *S* in P(t,r) is $\hat{\underline{n}}=\left(\frac{\partial P(t,r)}{\partial t}\wedge \frac{\partial P(t,r)}{\partial r}\right)∥\frac{\partial P(t,r)}{\partial t}\wedge \frac{\partial P(t,r)}{\partial r}{∥}^{-1}=\left(\frac{dg(r)}{dr}\cos t,\frac{dg(r)}{dr}\sin t,-\frac{d\bar{f}\left(r\right)}{dr}\right)$. Moreover, the coefficients of the second fundamental form become:

(A5) $$e=\frac{\partial^{2}P\left(t,r\right)}{\partial r^{2}}\times \hat{\underline{n}}=-\bar{f}\left(r\right)\frac{dg(r)}{dr};\;\;\;f=\frac{\partial^{2}P\left(t,r\right)}{\partial r\partial t}\times \hat{\underline{n}}=0;\;\;\;g=\frac{d^{2}\bar{f}\left(r\right)}{dr^{2}}\frac{dg(r)}{dr}-\frac{d\bar{f}\left(r\right)}{dr}\frac{d^{2}\bar{g}\left(r\right)}{dr^{2}}.$$

To obtain both the principal curvatures, ${\bar{k}}_{1}\left(t,r\right)$ and ${\bar{k}}_{2}\left(t,r\right)$, one needs to solve $\left(e-kE\right)\left(g-kG\right)-{\left(f-kF\right)}^{2}=0$, achieving ${\bar{k}}_{1}\left(t,r\right)=-\frac{\frac{dg(r)}{dr}}{\bar{f}\left(r\right)}$ and ${\bar{k}}_{2}\left(t,r\right)=\frac{d^{2}\bar{f(r)}}{dr^{2}}\frac{dg(r)}{dr}-\frac{d\bar{f}\left(r\right)}{dr}\frac{d^{2}\bar{g}\left(r\right)}{r^{2}}$. Then, the mean curvature, H(t,r), becomes $H\left(t,r\right)=\frac{1}{2}\left({\bar{k}}_{1}\left(t,r\right)+{\bar{k}}_{2}\left(t,r\right)\right)=\frac{1}{2}\left(-\frac{\frac{dg(r)}{dr}}{\bar{f}\left(r\right)}+\frac{d^{2}\bar{f}\left(r\right)}{dr^{2}}\frac{dg(r)}{dr}-\frac{d\bar{f}\left(r\right)}{dr}\frac{d^{2}\bar{g}\left(r\right)}{r^{2}}\right)$ and since *C* belongs to the plane y=0, one can set:

(A6) $$\bar{f}\left(r\right)=r;\;\;\;\frac{d\bar{f}\left(r\right)}{dr}=1;\;\;\;\frac{d\bar{f}\left(r\right)}{dr^{2}}=0;\;\;\;\bar{g}\left(r\right)=u\left(r\right)\;\;\;\;\frac{d\bar{g}\left(r\right)}{dr}=\frac{du(r)}{dr};\;\;\;\frac{d\bar{g}\left(r\right)}{dr^{2}}=\frac{d^{2}u\left(r\right)}{dr^{2}}$$

in which both $\frac{d\bar{f}\left(r\right)}{dr}$ and $\frac{d\bar{g}\left(r\right)}{dr}$ satisfy (A1). Thus, considering (A6), H(t,r) becomes $H\left(r\right)=-\frac{1}{2}\left(\frac{1}{r}\frac{du(r)}{dr}+\frac{d^{2}u\left(r\right)}{dr^{2}}\right)$. Therefore, by H(r), (22) becomes:

(A7) $$\left\{\begin{array}{c} \frac{d^{2}u\left(r\right)}{dr^{2}}+\frac{1}{r}\frac{du(r)}{dr}=-\frac{\theta \lambda^{2}}{4{\left(1-u\left(r\right)-d^{*}\right)}^{2}}{\left(\frac{1}{r}\frac{du(r)}{dr}+\frac{d^{2}u\left(r\right)}{dr^{2}}\right)}^{2}\\ u\left(R\right)=0;\;\;\;\frac{du(0)}{dr}=0;\;\;\;0<u\left(r\right)<d. \end{array}\right.$$

Finally, one can divide both sides of the equation of (A7) by $\frac{d^{2}u\left(r\right)}{dr^{2}}+\frac{1}{r}\frac{du(r)}{dr}$, achieving $\frac{d^{2}u\left(r\right)}{dr^{2}}=-\frac{1}{r}\frac{du(r)}{dr}-\frac{{\left(1-u\left(r\right)-d^{*}\right)}^{2}}{\theta \lambda^{2}}$, so that eqrefmedia can be written as (24).

## Appendix B

As known, differentiating (20), one obtains:

(A8) $$u\left(x\right)=\int_{-0.5}^{0.5}G\left(x,s\right)f\left(s,u\left(s\right),\frac{du(s)}{ds}\right)ds$$

where $0<u<h-d^{*}$ and G(x,s) is a suitable Green’s function [34]. Moreover,

(A9) $$\frac{du(x)}{dx}=\int_{-0.5}^{0.5}G_{x}\left(x,s\right)f\left(s,u\left(s\right),\frac{du(s)}{ds}\right)ds$$

so that (A8) becomes:

(A10) $$u\left(x\right)=\int_{-0.5}^{0.5}G\left(x,s\right)\frac{{\left(1+{\left(\frac{du(s)}{ds}\right)}^{2}\right)}^{3}}{\theta \mu^{2}\left(s,u\left(s\right),\lambda \right)}ds.$$

The existence of the solution of the equation T(u)=w, where $u\in P_{1}$, can be proved by the Schauder–Tychonoff fixed point theorem applied to w=T(u) from *P* to *P*[34]. In fact, from (A10), one defines the positive operator *T* as follows:

(A11) $$T\left(u\left(x\right)\right)=\int_{-0.5}^{0.5}G\left(x,s\right)\frac{{\left(1+{\left(\frac{du(s)}{ds}\right)}^{2}\right)}^{3}}{\theta \mu^{2}\left(s,u\left(s\right),\lambda \right)}ds$$

and

(A12) $$\frac{dT(u(x))}{dx}=\int_{-0.5}^{0.5}\frac{G(x,s)}{dx}\frac{{\left(1+{\left(\frac{du(s)}{ds}\right)}^{2}\right)}^{3}}{\theta \mu^{2}\left(s,u\left(s\right),\lambda \right)}ds.$$

The suitable Green’s function is [34] $G\left(x,s\right)=\frac{\left(s+0.5)(0.5-x\right)}{1}$, if −0.5≤s≤x; or $G\left(x,s\right)=\frac{\left(0.5-s)\times (x+0.5\right)}{1}$, when x≤s≤0.5 achieving $\frac{dG(x,s)}{dx}=\frac{-(s+0.5)}{1}$ if −0.5≤s≤x, and $\frac{dG(x,s)}{dx}=\frac{\left(0.5-s\right)}{1}$ if x≤s≤0.5. Moreover, G(x,s) is a continuous and non-negative function having its maximum equal to 0.5/2 on the straight line x=s at s=0. From which, one can write:

(A13) 0≤G(x,s)≤0.5/2∀x,s∈[−0.5,0.5];

In addition, the integral over [0.5,0.5] becomes

$$\int_{-0.5}^{0.5}G\left(x,s\right)ds=\frac{0.5-x}{1}\int_{-0.5}^{x}\left(s+0.5\right)ds+\frac{x+0.5}{1}\int_{x}^{0.5}\left(0.5-s\right)ds\leq \frac{0.5^{2}}{2};$$

and the integral of $\left|\frac{dG(x,s)}{dx}\right|$ over [−0.5,0.5] becomes:

(A14) $$|\int_{-0.5}^{0.5}\frac{dG(x,s)}{dx}ds|\leq \int_{-0.5}^{0.5}|\frac{dG(x,s)}{dx}|ds\leq 0.5.$$

Finally, ∀x,s∈([−0.5,0.5]×[−0.5,0.5]):

(A15) $$\frac{dG(x,s)}{dx}\leq 0.5.$$

## Appendix C. Proof of Theorem 1

To prove that $T\left(u\right)\in C_{0}^{2}\left(\left[-0.5,0.5\right]\right)$, one must prove that

(A16) $${l∥T\left(u\left(x\right)\right)∥}_{C^{2}\left[-0.5,0.5\right]}=\underset{x\in [-0.5,0.5]}{\sup}\left|T\left(u\left(x\right)\right)\right|+\underset{x\in [-0.5,0.5]}{\sup}|\frac{dT(u(x))}{dx}|+\underset{x\in [-0.5,0.5]}{\sup}|\frac{d^{2}T\left(u\left(x\right)\right)}{dx^{2}}|<+\infty .$$

Due to the construction of G(x,s), it follows that T(u(−0.5))=T(u(0.5))=0 verifying that T(u)≥0. Moreover, it is easy to verify that μ(x,u(x))>1 in [−0.5,0.5]. This is due to the fact that E deforms the membrane satisfying the condition that locally it must win the inertion of the deformation, so that μ(x,u(x)) assumes values greater than the unity. Therefore, to overcome the inertia of the membrane, a minimum voltage $\bar{\lambda }>0$ should be applied, so that $\bar{\lambda }<\lambda <\sup\left\{\lambda \right\}$ and sup{λ} being a bounded quantity, it follows that $1/\lambda^{2}<+\infty$. Therefore, also considering the (A13), one can write:

(A17) $$\begin{array}{c} 0\leq \left|T\left(u\left(x\right)\right)\right|\leq sup_{x\in [-0.5,0.5]}\left|T\left(u\left(x\right)\right)\right|\leq \\ \leq \frac{1}{\theta \lambda^{2}}\underset{x\in [-0.5,0.5]}{\sup}|\int_{-0.5}^{x}\frac{\left(s+0.5)(0.5-x\right)}{1}{\left(1+{\left(\frac{du(s)}{ds}\right)}^{2}\right)}^{3}{\left(\left(h-d^{*}\right)-u\left(s\right)\right)}^{2}ds|+\\ +\frac{1}{\theta \lambda^{2}}\underset{x\in [-0.5,0.5]}{\sup}|\int_{x}^{L}\frac{\left(0.5-s)(x+0.5\right)}{1}{\left(1+{\left(\frac{du(s)}{ds}\right)}^{2}\right)}^{3}{\left(\left(h-d^{*}\right)-u\left(s\right)\right)}^{2}ds|\leq \\ \leq 4\left(h-d^{*}\right)\frac{1}{\theta \lambda^{2}}\left(1+H^{6}\right)0.5^{2}<+\infty . \end{array}$$

Moreover:

(A18) $$\begin{array}{c} \underset{x\in [-0.5,0.5]}{\sup}|\frac{dT(u(x))}{dx}|=\underset{x\in [-0.5,0.5]}{\sup}|\int_{-0.5}^{0.5}\frac{G_{(}x,s)}{dx}\frac{{\left(1+{\left(\frac{du(s)}{ds}\right)}^{2}\right)}^{3}}{\theta \mu^{2}}ds|\leq 2\left(h-d^{*}\right)\frac{1}{\theta \lambda^{2}}\left(1+H^{6}\right)<+\infty . \end{array}$$

Thus, being $|\frac{du(x)}{dx}|\leq H$ and $|1/\mu^{2}|<1$, it is easy to obtain:

(A19) $$\begin{array}{c} \underset{x\in ([-0.5,0.5])}{\sup}|\frac{d^{2}T\left(u\left(x\right)\right)}{dx^{2}}|=\underset{x\in ([-0.5,0.5])}{\sup}|\frac{d}{dx}\int_{-0.5}^{0.5}G_{x}\left(x,s\right)\left(\frac{{\left(1+{\left(\frac{du(s)}{ds}\right)}^{2}\right)}^{3}}{\theta \mu^{2}}\right)ds|\leq \\ \leq \frac{1}{2\theta \lambda^{2}}\underset{x\in ([-0.5,0.5])}{\sup}|\left(\frac{{\left(1+{\left(\frac{du(s)}{ds}\right)}^{2}\right)}^{3}}{\theta \mu^{2}}\right)|+\frac{1}{2\theta \lambda^{2}}\underset{x\in ([-0.5,0.5])}{\sup}|\left(\frac{{\left(1+{\left(\frac{du(s)}{ds}\right)}^{2}\right)}^{3}}{\theta \mu^{2}}\right)|\leq \\ \leq \left(\frac{1}{2\theta }+\frac{1}{2\theta }\right)\frac{{\left(1+H^{2}\right)}^{3}}{\lambda^{2}}=\frac{1}{\theta }\frac{{\left(1+H^{2}\right)}^{3}}{\lambda^{2}}<+\infty . \end{array}$$

Moreover, we can deduce that:

(A20) $$\begin{array}{c} ∥T(u\left(∥\right)|{|}_{C^{2}\left(\left[-0.5,0.5\right]\right)}\leq \frac{\left(h-d^{*}\right)}{\theta \lambda^{2}}\left(1+H^{6}\right)+4\left(h-d^{*}\right)\frac{0.5(1+H^{6})}{\theta \lambda^{2}}+\frac{{\left(1+H^{2}\right)}^{3}}{\theta \lambda^{2}}<+\infty . \end{array}$$

To prove that T(u)∈P, one considers that $4\left(h-d^{*}\right)\frac{1}{\theta \lambda^{2}}\left(1+H^{6}\right)0.5^{2}<\left(h-d^{*}\right)$, so that $1+H^{6}<\theta {\bar{\lambda }}^{2}$ from which $H<\sqrt[{6}]{\theta {\bar{\lambda }}^{2}-1}$. Therefore, $1+H^{6}<\frac{H\theta {\bar{\lambda }}^{2}}{2(h-d^{*})}$∧$1+H^{6}<\theta {\bar{\lambda }}^{2}$ is verified. To compare the second members, it is sufficent to suppose, by contradiction, that $\theta {\bar{\lambda }}^{2}<\frac{H\theta \bar{\lambda^{2}}}{2(h-d^{*})}$ obtaining $H>\frac{\left(h-d^{*}\right)}{0.5}=2\left(h-d^{*}\right)$. However, remembering the scaling factors (4) and considering that $H=\frac{z}{x}$ and $H^{\prime }=\frac{z^{\prime }}{x^{\prime }}$, one can write:

(A21) $$H=\frac{z}{x}=\frac{z^{\prime }}{h}\frac{1}{x^{\prime }}=H^{\prime }\frac{1}{h}>2\left(h-d^{*}\right).$$

Moreover, in no-scaling conditions, $\left(h-d^{*}\right)=\frac{{\left(h-d^{*}\right)}^{\prime }}{h}$ and by (A21) one easily obtain $H^{\prime }>\frac{{\left(h-d^{*}\right)}^{\prime }}{L}$. Since *L* represents the semi-length of the wafer without scaling operation, thus, if L→0, it follow that $\frac{{\left(h-d^{*}\right)}^{\prime }}{L}\to +\infty$ and being ${\left(h-d^{*}\right)}^{\prime }$ a positive limited coefficient, then $H^{\prime }=\sup|\frac{du(x)}{dx}|$ would be greater than a quantity tending to +∞ inconsistently with the membership of *u* to $C^{2}\left(\left[-0.5,0.5\right]\right)$. Therefore, it follows that $\theta {\bar{\lambda }}^{2}>\frac{H\theta {\bar{\lambda }}^{2}}{2(h-d^{*})}$ from which:

(A22) $$1+H^{6}<\frac{H\theta {\bar{\lambda }}^{2}}{2(h-d^{*})}$$

which represents the condition guaranteeing that T(u):P→P.

## Appendix D. Proof of Theorem 2

By Theorem 1 and taking into account that the following compact immersions $C_{0}^{2}\left[-0.5,0.5\right]↪C_{0}^{1}\left[-0.5,0.5\right]$ and $P_{1}↪P$ holds, applying the Schauder–Tychonoff fixed-point theorem, u(x)=T(w(x)) admits at least a fixed point u(x)=T(u(x)) in $P_{1}$. In other words, there exists at least a solution for (17).

## Appendix E. Proof of Theorem 3

Let us introduce the following Lemma.

**Lemma** **A1.** *Considering the problem* (25)*, let $u_{1}\left(r\right)$ and $u_{2}\left(r\right)$ be twice continuously differentiable functions in order that $u_{1}\left(r\right)<u_{2}\left(r\right)$, r∈(0,1) and*

(A23) $$\frac{d^{2}u_{1}\left(r\right)}{dt^{2}}+F\left(r,u_{1}\left(r\right),\frac{du_{1}\left(r\right)}{dr}\right)>0\;\;\;\;\frac{d^{2}u_{2}\left(r\right)}{dr^{2}}+F\left(r,u_{2}\left(r\right),\frac{du_{2}\left(r\right)}{dr}\right)<0$$

*for r∈(0,1). Furthermore, let $F\left(r,y,\frac{dy}{dr}\right)$ be a continuous function satisfying the following generalized Lipschitz condition:*

(A24) $$\begin{array}{c} K_{1}\left(r\right)\left(u\left(r\right)-v\left(r\right)\right)+L_{2}\left(r\right)\left(\frac{du(r)}{dr}-\frac{dv(r)}{dr}\right)\leq F\left(r,u\left(r\right),\frac{du(r)}{dr}\right)-F\left(r,v\left(r\right),\frac{dv(r)}{dr}\right)\leq \\ \leq K_{2}\left(r\right)\left(u\left(r\right)-v\left(r\right)\right)+L_{1}\left(r\right)\left(\frac{du(r)}{dr}-\frac{dv(r)}{dr}\right), \end{array}$$

*in U×(−∞,+∞), in which $U=\{\left(r,u\right)\;:\;0<r<1\;\;and\;\;u_{1}\left(r\right)\leq u\left(r\right)\leq u_{2}\left(r\right)\}$ and $K_{i}\left(r\right)$ and $L_{i}\left(r\right)$ (i=1,2) are continuous functions in (0,1]. In the case in which $\frac{du_{1}\left(0\right)}{dr}\geq \frac{du_{2}\left(0\right)}{dr}$, such that $u_{1}\left(1\right)=B=u_{2}\left(1\right)$, thus* (25) *has at least one solution, u(r), satisfying the following chain of inequalities $u_{1}\left(r\right)\leq u\left(r\right)\leq u_{2}\left(r\right)$.*

To prove Theorem 1, it is imperative to exploit the Lemma A1. Particularly, one assumes the following expressions as $u_{1}\left(r\right)$ and $u_{2}\left(r\right)$, respectively:

(A25) $$u_{1}\left(r\right)=0\;\;\forall r\in \left[0,1\right]$$

and

(A26) $$u_{2}\left(r\right)=\bar{u}\left(r\right)=\frac{k\epsilon_{0}V^{2}}{2d^{*2}}\left\{1-r^{2}\right\}.$$

By construction, $u_{1}\left(r\right)<u_{2}\left(r\right)$ and both are twice continuously differentiable functions. At this point, one has to verify (A65). In other words:

(A27) $$\frac{d^{2}u_{1}\left(r\right)}{dt^{2}}+F\left(r,u_{1}\left(r\right),\frac{du_{1}\left(r\right)}{dr}\right)=\frac{d^{2}u_{1}\left(r\right)}{dt^{2}}+\frac{1}{r}\frac{du_{1}\left(r\right)}{dr}+\frac{{\left(1-u_{1}\left(r\right)-d^{*}\right)}^{2}}{\theta \lambda^{2}}>0,$$

(A28) $$\frac{d^{2}u_{2}\left(r\right)}{dt^{2}}+F\left(r,u_{2}\left(r\right),\frac{du_{2}\left(r\right)}{dr}\right)=\frac{d^{2}u_{2}\left(r\right)}{dt^{2}}+\frac{1}{r}\frac{du_{2}\left(r\right)}{dr}+\frac{{\left(1-u_{2}\left(r\right)-d^{*}\right)}^{2}}{\theta \lambda^{2}}<0.$$

(A27) is verifiable observing that, for $u_{1}\left(r\right)=0$ and ∀r∈[0,1], it follows that $\frac{du_{1}\left(r\right)}{dr}=\frac{d^{2}u_{1}\left(r\right)}{dr^{2}}=0$. Then, assuming that

(A29) $$\theta \lambda^{2}>0,$$

(A27) is directly verified. Moreover, to verify (A28), with (A26)), one easily obtain

(A30) $$\frac{du_{2}\left(r\right)}{dr}=-\frac{\epsilon_{0}kV^{2}r}{d^{*2}}$$

and again

(A31) $$\frac{d^{2}u_{2}\left(r\right)}{dr^{2}}=-\frac{\epsilon_{0}kV^{2}}{d^{*2}}.$$

Thus, by both (A30) and (A31), (A27) becomes

(A32) $$\frac{1}{\theta \lambda^{2}}{\left\{1-\frac{\epsilon_{0}kV^{2}}{2d^{*2}}\left\{1-r^{2}\right\}\right\}}^{2}<\frac{2\epsilon_{0}kV^{2}}{d^{*2}}.$$

Moreover, in (A32), ${\left\{1-\frac{\epsilon_{0}kV^{2}}{2d^{*2}}\left\{1-r^{2}\right\}\right\}}^{2}<1$, such that, imposing $\frac{1}{\theta \lambda^{2}}<\frac{2\epsilon_{0}kV^{2}}{d^{*2}}$, one achieves

(A33) $$\theta \lambda^{2}>\frac{d^{*2}}{2V^{2}\epsilon_{0}k}$$

that automatically satisfies (A29). Lemma A1 also requires, to prove that $F\left(r,u\left(r\right),\frac{du(r)}{dr}\right)=\frac{1}{r}\frac{du(r)}{dr}+\frac{{\left(1-u\left(r\right)\right)}^{2}}{\theta \lambda^{2}}$ satisfies the Lipschitz condition (A24). Thus, we can easily write:

(A34) $$\begin{array}{c} F\left(r,u\left(r\right)-v\left(r\right),\frac{du(r)}{dr}-\frac{dv(r)}{dr}\right)=\frac{1}{r}\frac{du(r)}{dr}+\frac{{\left(1-u\left(r\right)\right)}^{2}}{\theta \lambda^{2}}-\frac{1}{r}\frac{dv(r)}{dr}-\frac{{\left(1-v\left(r\right)\right)}^{2}}{\theta \lambda^{2}}\geq \\ \geq \frac{1}{r}\left(\frac{u(r)}{dr}-\frac{dv(r)}{dr}\right)-\frac{2}{\theta \lambda^{2}}\left(u(r)-v(r)\right)=L_{2}\left(r\right)\left(\frac{du(r)}{dr}-\frac{dv(r)}{dr}\right)+K_{1}\left(r\right)\left(u(r)-v(r)\right). \end{array}$$

Moreover,

(A35) $$\begin{array}{c} \frac{1}{r}\left(\frac{u(r)}{dr}-\frac{dv(r)}{dr}\right)-\frac{1}{\theta \lambda^{2}}\left((u(r)-v(r))(2-(u(r)+v(r))\right)\leq \\ \leq L_{1}\left(r\right)\left(\frac{du(r)}{dr}-\frac{dv(r)}{dr}\right)+K_{2}\left(r\right)\left(Z(u(r)-v(r))\right). \end{array}$$

2−(u(r)+v(r))≥0, so there exists a constant *Z* such that 0<Z<2−(u(r)+v(r)) holds. Finally, Lemma A1 also requires that $\frac{du_{1}\left(0\right)}{dr}\geq \frac{du_{2}\left(0\right)}{dr}$. For this aim, being a=0, it follows that $\frac{du_{1}\left(0\right)}{dr}=\frac{du_{1}\left(0\right)}{dr}=0$. Moreover, $\frac{du_{2}\left(0\right)}{dr}=\frac{du_{2}\left(0\right)}{dr}=0$. Furthermore, $u_{1}\left(1\right)=u_{2}\left(1\right)=0$, which completes the proof of the Theorem.

## Appendix F. Proof of Theorem 4

From the equation of model (17), it is easy to infer that, being $\frac{d^{2}u\left(x\right)}{dx^{2}}\leq 0$ in [−0.5,0.5], u(x) is concave over the same interval while its first derivative decreases. Moreover, this equation is writable as follows:

(A36) $$\frac{d^{2}u\left(x\right)}{dx^{2}}{\left[1+{\left(\frac{du(x)}{dx}\right)}^{2}\right]}^{-3}=-\frac{1}{\theta \bar{\lambda^{2}}}{\left[h-d^{*}-u\left(x\right)\right]}^{2}.$$

Multiplying both members of (A36) by $\frac{u(x)}{dx}$, one easily achieves:

(A37) $$\begin{array}{c} \frac{d^{2}u\left(x\right)}{dx^{2}}\frac{du(x)}{dx}{\left[1+{\left(\frac{du(x)}{dx}\right)}^{2}\right]}^{-3}=-\frac{1}{\theta {\bar{\lambda }}^{2}}{\left(h-d^{*}\right)}^{2}\frac{du(x)}{dx}+\frac{1}{\theta {\bar{\lambda }}^{2}}\left(h-d^{*}\right)\frac{d}{dx}{\left[u\left(x\right)\right]}^{2}-\frac{1}{3\;\theta {\bar{\lambda }}^{2}}\frac{d}{dx}{\left[u\left(x\right)\right]}^{3}. \end{array}$$

Since $\frac{d^{2}u\left(x\right)}{dx^{2}}\frac{du(x)}{dx}{\left[1+{\left(\frac{du(x)}{dx}\right)}^{2}\right]}^{-2}=-\frac{1}{4}\frac{d}{dx}{\left[1+{\left(\frac{du(x)}{dx}\right)}^{2}\right]}^{-2}$ by integration from −0.5 to 0.5, (A37), one achieves

(A38) $$-\;\frac{1}{4}\;\frac{1}{{\left[1+{\left(\frac{du(-0.5)}{dx}\right)}^{2}\right]}^{2}}+\;\frac{1}{4}\frac{1}{{\left[1+{\left(\frac{du(0.5)}{dx}\right)}^{2}\right]}^{2}}=0,$$

from which $\left|\frac{du(-0.5)}{dx}|=|\frac{du(0.5)}{dx}\right|$. Furthermore, by integration of (A37) from −0.5 to *t*, and considering that u(−L)=0 and that ∀t∈[−0.5,0.5] one can achieve:

(A39) $$\begin{array}{c} -\frac{0.25}{{\left[1+{\left(\frac{du(t)}{dx}\right)}^{2}\right]}^{2}}+\frac{0.25}{{\left[1+{\left(\frac{du(-0.5)}{dx}\right)}^{2}\right]}^{2}}=-\frac{1}{\theta \bar{\lambda^{2}}}{\left(h-d^{*}\right)}^{2}u\left(t\right)+\frac{1}{\theta {\bar{\lambda }}^{2}}\left(h-d^{*}\right){\left[u\left(t\right)\right]}^{2}-\frac{1}{3\;\theta \bar{\lambda^{2}}}{\left[u\left(t\right)\right]}^{3}, \end{array}$$

so that ∀t∈[−0.5,0.5], $-\frac{1}{\theta {\bar{\lambda }}^{2}}{\left(h-d^{*}\right)}^{2}u\left(t\right)+\frac{1}{\theta {\bar{\lambda }}^{2}}\left(h-d^{*}\right){\left[u\left(t\right)\right]}^{2}-\frac{1}{3\;\theta {\bar{\lambda }}^{2}}{\left[u\left(t\right)\right]}^{3}<0$ and $-\frac{1}{4}\;\frac{1}{{\left[1+{\left(\frac{du(t)}{dx}\right)}^{2}\right]}^{3}}+\frac{1}{4}\frac{1}{{\left[1+{\left(\frac{du(-0.5)}{dx}\right)}^{2}\right]}^{3}}<0$. Thus, ∀t∈[−0.5,0.5], $\left|\frac{du(t)}{dx}|<|\frac{du(-0.5)}{dx}\right|$. To prove that (17) has only one solution, let us suppose, by contradiction, that (17) admits two different solutions, $u_{1},\;u_{2}\in P_{1}$. From the equation associated to (17), integrating from −0.5 to *t*, ∀t∈[−0.5,0.5], one achieves $\frac{du_{1}\left(t\right)}{dx}\leq H-\frac{1}{\theta \bar{\lambda^{2}}}\int_{-0.5}^{t}{\left[1+{\left(\frac{du_{1}\left(x\right)}{dx}\right)}^{2}\right]}^{3}{\left[h-d^{*}-u_{1}\left(x\right)\right]}^{2}dx$ and $\frac{du_{2}\left(t\right)}{dx}\leq H-\frac{1}{\theta \bar{\lambda^{2}}}\int_{-0.5}^{t}{\left[1+{\left(\frac{du_{2}\left(x\right)}{dx}\right)}^{2}\right]}^{3}{\left[h-d^{*}-u_{2}\left(x\right)\right]}^{2}dx$. Subtracting on both members, ∀t∈[−0.5,0.5]

(A40) $$\begin{array}{c} \frac{du_{1}\left(t\right)}{dx}-\frac{du_{2}\left(t\right)}{dx}=\\ =\frac{1}{\theta \bar{\lambda^{2}}}\int_{-0.5}^{t}\left\{{\left[1+{\left(\frac{du_{2}\left(x\right)}{dx}\right)}^{2}\right]}^{3}{\left[h-d^{*}-u_{2}\left(x\right)\right]}^{2}-{\left[1+{\left(\frac{du_{1}\left(x\right)}{dx}\right)}^{2}\right]}^{3}{\left[h-d^{*}-u_{1}\left(x\right)\right]}^{2}\right\}dx. \end{array}$$

To evaluate the term in the integral, let us consider $F\left(w,v\right)={\left[1+w^{2}\right]}^{3}{\left(h-d^{*}-v\right)}^{2}$ and $g\left(t\right)=F\left(tw_{1}+\left(1-t\right)w_{2},tv_{1}+\left(1-t\right)v_{2}\right)=F\left(w_{t},v_{t}\right)$. Observing that $\frac{dg(t)}{dx}=\frac{\partial F(w_{t},v_{t})}{\partial w}\left(w_{1}-w_{2}\right)+\frac{\partial F(w_{t},v_{t})}{\partial v}\left(v_{1}-v_{2}\right)$ and $g\left(1\right)=F\left(w_{1},v_{1}\right),\;g\left(0\right)=F\left(w_{2},v_{2}\right),\;g\left(1\right)-g\left(0\right)=g^{\prime }\left(\xi \right),\;\xi \in \left(0,1\right)$, one can easily write

(A41) $$\begin{array}{c} \frac{\partial F(w_{\xi },v_{\xi })}{\partial w}=6{\left[1+w_{\xi }^{2}\right]}^{2}w_{\xi }{\left(h-d^{*}-v_{\xi }\right)}^{2}=\\ \leq 6\left\{\xi {\left[1+w_{1}^{2}\right]}^{2}+\left(1-\xi \right){\left[1+w_{2}^{2}\right]}^{2}\right\}\left[\xi w_{1}+\left(1-\xi \right)w_{2}\right]{\left(h-d^{*}-v_{\xi }\right)}^{2}. \end{array}$$

Since $w_{1}\leq H$, $w_{2}\leq H$, $v_{\xi }\leq 1$, it results that $\left|\frac{\partial F(w_{\xi },v_{\xi })}{\partial w}\right|\leq 24{\left(1+H^{2}\right)}^{2}H$ and

(A42) $$\begin{array}{c} \left|\frac{\partial F(w_{\xi },v_{\xi })}{\partial v}\right|\leq 2\left|\xi {\left(1+w_{1}^{2}\right)}^{3}+\left(1-\xi \right){\left(1+w_{2}^{2}\right)}^{3}\right|\leq 4{\left(1+H^{2}\right)}^{3}. \end{array}$$

Considering the last inequality, by means of (A40) and exploiting the Poincaré’s inequality, ∀t∈[−0.5,0.5]

(A43) $$\begin{array}{c} |\frac{du_{1}\left(t\right)}{dx}-\frac{du_{2}\left(t\right)}{dx}|\leq \frac{24}{\theta {\bar{\lambda }}^{2}}{\left(1+H^{2}\right)}^{2}H\int_{-0.5}^{t}|\frac{du_{1}\left(x\right)}{dx}-\frac{du_{2}\left(x\right)}{dx}|dx+\\ +\frac{4}{\theta \bar{\lambda^{2}}}{\left(1+H^{2}\right)}^{3}\int_{-0.5}^{t}|\frac{du_{1}\left(x\right)}{dx}-\frac{du_{2}\left(x\right)}{dx}|dx\leq \frac{24}{\theta {\bar{\lambda }}^{2}}{\left(1+H^{2}\right)}^{2}H\int_{-0.5}^{t}|\frac{du_{1}\left(x\right)}{dx}-\frac{du_{2}\left(x\right)}{dx}|dx+\\ +\frac{8L}{\theta {\bar{\lambda }}^{2}}{\left(1+H^{2}\right)}^{3}\int_{-0.5}^{t}|\frac{du_{1}\left(x\right)}{dx}-\frac{du_{2}\left(x\right)}{dx}|\;\;\;dx\leq c\left(H,L,\bar{\lambda },\theta \right)\int_{-0.5}^{t}|\frac{du_{1}\left(x\right)}{dx}-\frac{du_{2}\left(x\right)}{dx}|\;\;\;dx. \end{array}$$

Then $|\frac{du_{1}\left(t\right)}{dx}-\frac{du_{2}\left(t\right)}{dx}|\leq c\left(H,L,\bar{\lambda },\theta \right)\int_{-0.5}^{t}|\frac{du_{1}\left(x\right)}{dx}-\frac{du_{2}\left(x\right)}{dx}|dx$ from which, exploiting the well-known Gronwall’s lemma, one has, ∀t∈[−0.5,0.5], $|\frac{du_{1}\left(t\right)}{dx}-\frac{du_{2}\left(t\right)}{dx}|\leq 0$. Thus, ∀t∈[−L,L], it follows that $\frac{du_{1}\left(t\right)}{dx}-\frac{du_{2}\left(t\right)}{dx}=0$. In other words, $u_{1}-u_{2}=constant$, and considering that $u_{1}\left(-0.5\right)=u_{2}\left(-0.5\right)=u_{1}\left(0.5\right)=u_{2}\left(0.5\right)=0$, one has $u_{1}=u_{2}$. To prove the statement 2, let us consider u(x) as a solution for (17). Setting v(t)=u(−t), ∀t∈[−0.5,0.5], one has that also *v* is a solution of the same problem. In fact, $\frac{dv(t)}{dx}=-\frac{du(t)}{dt}$, $\frac{d^{2}v\left(t\right)}{dx^{2}}=-\frac{d^{2}u\left(-t\right)}{dx^{2}}$ so that, one obtains $\frac{d^{2}u\left(-t\right)}{dx^{2}}=-\frac{{\left[1+{\left(\frac{du(-t)}{dx}\right)}^{2}\right]}^{3}}{\theta \bar{\lambda^{2}}}\left(h-d^{*}-u\left(-t\right)\right)$. Therefore, as already mentioned, $v^{\prime }\left(-0.5\right)=-u^{\prime }\left(0.5\right)=u^{\prime }\left(-0.5\right)\leq H$, so that v(t)=u(t), ∀t∈[−0.5,0.5], so that u(t)=u(−t) over [−0.5,0.5].

To prove the statement 3, it is sufficient to take into account that $u\left(x\right)\in C^{2}$, and the second member of the equation $\in C^{1}$ and than $u\left(x\right)\in C^{3}\left(\left[-0.5,0.5\right]\right)$. By induction, one obtains that $u\in C^{\infty }\left(\left[-05,0.5\right]\right)$.

## Appendix G. Proof of Theorem 5

To prove Theorem 5, we premise the following Lemmas. In particular, considering, by contradiction, two different solutions of $u_{1}\left(x\right)£$, $u_{2}\left(x\right)$, one obtains the following important results.

**Lemma** **A2.**
*The following inequality holds.*

(A44) $$|{\left(1+{\left(\frac{du_{2}\left(x\right)}{dx}\right)}^{2}\right)}^{3}-{\left(1+{\left(\frac{du_{1}\left(x\right)}{dx}\right)}^{2}\right)}^{3}|\leq 24H^{5}|\frac{du_{2}\left(x\right)}{dx}-\frac{du_{1}\left(x\right)}{dx}|.$$

**Proof.** Being H>1, from $\left|{\left(1+{\left(\frac{du_{2}\left(x\right)}{dx}\right)}^{2}\right)}^{3}-{\left(1+{\left(\frac{du_{1}\left(x\right)}{dx}\right)}^{2}\right)}^{3}\right|$ one can write:

(A45) $$\begin{array}{c} |{\left(1+{\left(\frac{du_{2}\left(x\right)}{dx}\right)}^{2}\right)}^{3}-{\left(1+{\left(\frac{du_{1}\left(x\right)}{dx}\right)}^{2}\right)}^{3}|=|\left[\left(\frac{du_{2}\left(x\right)}{dx}-\frac{du_{1}\left(x\right)}{dx}\right)\left(\frac{du_{2}\left(x\right)}{dx}+\frac{du_{1}\left(x\right)}{dx}\right)\right]\times \\ \times {\left[{\left(1+\frac{du_{1}\left(x\right)}{dx}\right)}^{2}\right)}^{2}+\left(1+{\left(\frac{du_{2}\left(x\right)}{dx}\right)}^{2}\right)(1+{\left(\frac{du_{1}\left(x\right)}{dx}\right)}^{2})+{\left(1+{\left(\frac{du_{1}\left(x\right)}{dx}\right)}^{2}\right)}^{2}]|\leq \\ \leq |\frac{du_{2}\left(x\right)}{dx}-\frac{du_{1}\left(x\right)}{dx}|2H\left|3{\left(1+H^{2}\right)}^{2}\right|\leq 24H^{5}|\frac{du_{2}\left(x\right)}{dx}-\frac{du_{1}\left(x\right)}{dx}|. \end{array}$$

 □

**Lemma** **A3.**
*Considering that $h-d^{*}<1$, and since $0<u<h-d^{*}$, $\forall \;u_{1}\left(x\right),u_{2}\left(x\right)\in P$, it easily follows:*

(A46) $$\begin{array}{c} |{\left(1+{\left(\frac{du_{2}\left(x\right)}{dx}\right)}^{2}\right)}^{3}{\left(h-d^{*}-u_{2}\left(x\right)\right)}^{2}-{\left(1+{\left(\frac{du_{1}\left(x\right)}{dx}\right)}^{2}\right)}^{3}{\left(h-d^{*}-u_{1}\left(x\right)\right)}^{2}|\leq \\ \leq 216H^{5}|\frac{du_{2}\left(x\right)}{dx}-\frac{du_{1}\left(x\right)}{dx}|+24\left(1+H^{6}\right)\left|u_{2}\left(x\right)-u_{1}\left(x\right)\right|. \end{array}$$

**Proof.** Taking into account

(A47) $$\left|{\left(1+{\left(\frac{du_{2}\left(x\right)}{dx}\right)}^{2}\right)}^{3}{\left(h-d^{*}-u_{2}\left(x\right)\right)}^{2}-{\left(1+{\left(\frac{du_{1}\left(x\right)}{dx}\right)}^{2}\right)}^{3}{\left(h-d^{*}-u_{1}\left(x\right)\right)}^{2}\right|$$

one can write:

(A48) $$\begin{array}{c} |{\left(1+{\left(\frac{du_{2}\left(x\right)}{dx}\right)}^{2}\right)}^{3}{\left(h-d^{*}-u_{2}\left(x\right)\right)}^{2}-{\left(1+{\left(\frac{du_{1}\left(x\right)}{dx}\right)}^{2}\right)}^{3}{\left(h-d^{*}-u_{1}\left(x\right)\right)}^{2}|=\\ =|{\left(1+{\left(\frac{du_{2}\left(x\right)}{dx}\right)}^{2}\right)}^{3}+d^{*}{\left(1+{\left(\frac{du_{2}\left(x\right)}{dx}\right)}^{2}\right)}^{3}+u_{2}^{2}{\left(1+{\left(\frac{du_{2}\left(x\right)}{dx}\right)}^{2}\right)}^{3}-2d^{*}{\left(1+{\left(\frac{du_{2}\left(x\right)}{dx}\right)}^{2}\right)}^{3}-\\ -2u_{2}\left(x\right){\left(1+{\left(\frac{du_{2}\left(x\right)}{dx}\right)}^{2}\right)}^{3}+2u_{2}\left(x\right)d^{*}{\left(1+{\left(\frac{du_{2}\left(x\right)}{dx}\right)}^{2}\right)}^{3}-\\ -{\left(1+{\left(\frac{du_{1}\left(x\right)}{dx}\right)}^{2}\right)}^{3}-d^{*}{\left(1+{\left(\frac{du_{1}\left(x\right)}{dx}\right)}^{2}\right)}^{3}-{\left(u_{1}\left(x\right)\right)}^{2}{\left(1+{\left(\frac{du_{1}\left(x\right)}{dx}\right)}^{2}\right)}^{3}+2d^{*}{\left(1+{\left(\frac{du_{1}\left(x\right)}{dx}\right)}^{2}\right)}^{3}+\\ +2u_{1}\left(x\right){\left(1+{\left(\frac{du_{1}\left(x\right)}{dx}\right)}^{2}\right)}^{3}-2u_{1}\left(x\right)d^{*}{\left(1+{\left(\frac{du_{1}\left(x\right)}{dx}\right)}^{2}\right)}^{3}|\leq \\ \leq 216H^{5}|\frac{du_{2}\left(x\right)}{dx}-\frac{du_{1}\left(x\right)}{dx}|+24\left(1+H^{6}\right)\left|u_{2}\left(x\right)-u_{1}\left(x\right)\right|. \end{array}$$

 □

Exploiting both Lemmas A2 and A3, one is ready to prove Theorem 5.

By contradiction, assuming $u_{1}\left(x\right),u_{2}\left(x\right)$∈P as two different solutions for (17), one can write $u_{1}\left(x\right)=T\left(u_{1}\left(x\right)\right)$ and $u_{2}\left(x\right)=T\left(u_{2}\left(x\right)\right)$.Then, it follows that:

(A49) $$\begin{array}{c} u_{1}\left(x\right)=\int_{-0.5}^{0.5}G\left(x,s\right)\frac{{\left(1+{\left(\frac{du_{1}\left(s\right)}{ds}\right)}^{2}\right)}^{3}}{\theta \mu^{2}\left(s,u_{1}\left(s\right),\lambda \right)}ds=\int_{-0.5}^{0.5}G\left(x,s\right)(\left(1+{\left(u_{1}^{\prime }{\left(s\right)}^{2}\right)}^{3}\right)\frac{{\left(h-d^{*}-u_{1}\left(s\right)\right)}^{2}}{\theta \lambda^{2}}ds \end{array}$$

(A50) $$\begin{array}{c} u_{2}\left(x\right)=\int_{-0.5}^{0.5}G\left(x,s\right)\frac{{\left(1+{\left(\frac{du_{2}\left(s\right)}{ds}\right)}^{2}\right)}^{3}}{\theta \mu^{2}\left(s,u_{2}\left(s\right),\lambda \right)}ds=\int_{-0.5}^{0.5}G\left(x,s\right)(\left(1+{\left(u_{2}^{\prime }{\left(s\right)}^{2}\right)}^{3}\right)\frac{{\left(h-d^{*}-u_{2}\left(s\right)\right)}^{2}}{\theta \lambda^{2}}ds \end{array}$$

(A51) $$\begin{array}{c} \frac{du_{1}\left(x\right)}{dx}=\int_{-0.5}^{0.5}\frac{1}{\theta \lambda^{2}}\frac{dG(x,s)}{dx}\left({\left(1+{\left(\frac{du_{1}\left(s\right)}{ds}\right)}^{2}\right)}^{3}\right){\left(h-d^{*}-u_{1}\left(s\right)\right)}^{2}ds \end{array}$$

(A52) $$\begin{array}{c} \frac{du_{2}\left(x\right)}{dx}=\int_{0.5}^{0.5}\frac{1}{\theta \lambda^{2}}\frac{dG(x,s)}{dx}\left({\left(1+{\left(\frac{du_{2}\left(s\right)}{ds}\right)}^{2}\right)}^{3}\right){\left(h-d^{*}-u_{2}\left(s\right)\right)}^{2}ds \end{array}$$

Therefore, one achieves:

(A53) $$\begin{array}{c} ∥u_{1}-u_{2}{∥}_{C^{1}\left(\left[-0.5,0.5\right]\right)}=\underset{x\in [-0.5,0.5]}{\sup}\left|u_{1}\left(x\right)-u_{2}\left(x\right)\right|+sup_{x\in [-0.5,0.5]}|\frac{du_{1}\left(x\right)}{dx}-\frac{du_{2}\left(x\right)}{dx}|. \end{array}$$

Therefore, it follows that:

(A54) $$\begin{array}{c} ∥T\left(u_{1}\left(x\right)\right)-T(u_{2}\left(x\right))∥=\frac{1}{\theta \lambda^{2}}\underset{x\in [0.5,0.5]}{\sup}|\int_{0.5}^{0.5}G\left(x,s\right)({\left(1+{\left(\frac{du_{1}\left(s\right)}{ds}\right)}^{2}\right)}^{3}){\left(h-d^{*}-u_{1}\left(s\right)\right)}^{2}ds-\\ -\int_{-0.5}^{0.5}G\left(x,s\right)\left({\left(1+{\left(\frac{du_{2}\left(s\right)}{ds}\right)}^{2}\right)}^{3}\right){\left(h-d^{*}-u_{2}\left(s\right)\right)}^{2}ds|+\\ +\frac{1}{\theta \lambda^{2}}\underset{x\in [-0.5,0.5]}{\sup}|\int_{-0.5}^{0.5}\frac{dG(x,s)}{dx}\left({\left(1+{\left(\frac{du_{1}\left(s\right)}{ds}\right)}^{2}\right)}^{3}\right){\left(h-d^{*}-u_{1}\left(s\right)\right)}^{2}ds-\\ -\int_{-0.5}^{0.5}\frac{dG(x,s)}{dx}\left({\left(1+{\left(\frac{du_{2}\left(s\right)}{ds}\right)}^{2}\right)}^{3}\right){\left(h-d^{*}-u_{2}\left(s\right)\right)}^{2}ds|\leq \\ \leq \frac{0.75}{\theta \lambda^{2}}\underset{x\in [-0.5,0.5]}{\sup}|\int_{-0.5}^{0.5}\left[(-{\left(1+{\left(\frac{du_{1}\left(s\right)}{ds}\right)}^{2}\right)}^{3}\right){\left(h-d^{*}-u_{1}\left(s\right)\right)}^{2}+\\ +{\left(1+{\left(\frac{du_{2}\left(s\right)}{ds}\right)}^{2}\right)}^{3})\left(h-d^{*}-u_{2}\left(s\right)\right)]ds| \end{array}$$

Considering (A48), it follows:

(A55) $$\begin{array}{c} ∥T\left(u_{1}\left(x\right)\right)-T\left(u_{2}\left(x\right)\right){∥}_{C^{1}\left(\left[0.5,0.5\right]\right)}\leq \\ \leq \frac{0.75}{\theta \lambda^{2}}\underset{x\in [-0.5,0.5]}{\sup}|\int_{-0.5}^{0.5}(216H^{5}|\frac{du_{2}\left(x\right)}{dx}-\frac{du_{1}\left(x\right)}{dx}|+24\left(1+H^{6}\right)|u_{2}\left(x\right)-u_{1}\left(x\right)\left|\right)ds|=\\ =\frac{0.75}{\theta \lambda^{2}}\left(216H^{5}\right)\underset{s\in [-0.5,0.5]}{\sup}|\frac{u_{2}\left(s\right)}{ds}-\frac{du_{1}\left(s\right)}{ds}|+\frac{0.75}{\theta \lambda^{2}}\left(24\left(1+H^{6}\right)\right)\underset{s\in [-0.5,0.5]}{\sup}\left|u_{2}\left(s\right)-u_{1}\left(s\right)\right|. \end{array}$$

However, being $u_{1}\left(x\right)=T\left(u_{1}\left(x\right)\right)$ and $u_{2}\left(x\right)=T\left(u_{2}\left(x\right)\right)$, by means of (A53) and (A55), one would obtain a contradiction if:

(A56) $$\frac{1}{\theta \lambda^{2}}\left(\frac{0.5}{2}+\frac{1}{2}\right)216H^{5}<1\;\;\;\wedge \;\;\;\frac{1}{\theta \lambda^{2}}\left(\frac{0.5}{2}+\frac{1}{2}\right)24\left(1+H^{6}\right)<1$$

that is:

(A57) $$216H^{5}<\frac{\theta \lambda^{2}}{0.75}\;\;\;\wedge \;\;\;24\left(1+H^{6}\right)<\frac{\theta \lambda^{2}}{0.75}.$$

Taking into account the first inequality of (A57), one can also write $H^{6}=H^{5}H<\frac{\theta \lambda^{2}}{162}H$ so that $1+H^{6}<1+\frac{\theta \lambda^{2}H}{162}$ so (A57) becomes:

(A58) $$1+H^{6}<1+\frac{\theta \lambda^{2}H}{162}\;\;\;\wedge \;\;\;1+H^{6}<\frac{\theta \lambda^{2}}{18}.$$

Furthermore, one can observe that:

(A59) $$\frac{\theta \lambda^{2}}{18}<1+\frac{\theta \lambda^{2}H}{162}.$$

In fact, starting from (A59), it follows that $9\theta \lambda^{2}<216\times 0.75+\theta \lambda^{2}H$ from which $H>9\left(1-\frac{24}{\theta \lambda^{2}}0.75\right)$. In fact, if by contradiction we would write $\frac{\theta \lambda^{2}}{18}>1+\frac{\theta \lambda^{2}H}{162}$ from which $H<9-\frac{163}{\theta \lambda^{2}}<0$. In other words, *H* would assume negative value (impossible condition). Thus, (A56) is equivalent to:

(A60) $$1+H^{6}<\frac{\theta \lambda^{2}}{18}.$$

## Appendix H. Proof of Theorem 6

We premise the following Lemma.

**Lemma** **A4.**
*Let us firstly suppose that the conditions of Lemma A1 are satisfied. Moreover, $u_{1}\left(r\right)$, $u_{2}\left(r\right)$ satisfy the given boundary conditions. If $\frac{d^{2}u\left(r\right)}{dr^{2}}+K_{2}\left(r\right)u\left(r\right)+L_{1}\left(r\right)\frac{du(r)}{dr}=0$ has a nontrivial solution satisfying zero boundary conditions on any sub-interval of [0,1], then the problem has only one solution u(r) with $u_{1}\left(r\right)\leq u\left(r\right)\leq u_{2}\left(r\right)$*

As presented in (A35), it follows that $L_{1}\left(r\right)\frac{du(r)}{dr}+K_{2}\left(r\right)Zu\left(r\right)=\frac{1}{r}\left(\frac{du(r)}{dr}\right)-\frac{Z}{\theta \lambda^{2}}u\left(r\right)$. Thus, by Lemma A4, we consider:

(A61) $$\frac{d^{2}u\left(r\right)}{dr^{2}}+\frac{1}{r}\left(\frac{du(r)}{dr}\right)-\frac{Z}{\theta \lambda^{2}}u\left(r\right)=0,$$

which represents a particular case of $r^{2}\frac{d^{2}u\left(r\right)}{dr^{2}}+\left(2\bar{k}+1\right)r\frac{du(r)}{dr}+\left(\alpha^{2}r^{2s}+\beta^{2}\right)u\left(r\right)=0$ (Bessel equation) with 2s≠0 and $\bar{k},\alpha ,s,\beta \in C$. In fact, one can write $\frac{d^{2}u\left(r\right)}{dr^{2}}+\frac{2\bar{k}+1}{r}\frac{du(r)}{dr}+\left(\frac{\alpha^{2}r^{2s}}{r^{2}}+\frac{\beta^{2}}{r^{2}}\right)u\left(r\right)=0$ that becomes $\frac{d^{2}u\left(r\right)}{dr^{2}}+\frac{1}{r}\frac{du(r)}{dr}+\alpha^{2}r^{2s-2}u\left(r\right)=0$. If 2s−2=0, then r=1, obtaining $\frac{d^{2}u\left(r\right)}{dr^{2}}+\frac{1}{r}\frac{du(r)}{dr}+\alpha^{2}u\left(r\right)=0$. Setting $\alpha^{2}=-\frac{Z}{\theta \lambda^{2}}\in C$, (A61) is achieved. According to Bessel theory [35,36], the general solution for (A61) can be expressed as a linear combination of two linearly independent Bessel functions, $J_{0}\left(\sqrt{\frac{Z}{\theta \lambda^{2}}}r\right)$ and $Y_{0}\left(\sqrt{\frac{Z}{\theta \lambda^{2}}}r\right)$, of the first and second kind of zeroth order, respectively. In other words $u\left(r\right)=c_{1}J_{0}\left(\sqrt{\frac{Z}{\theta \lambda^{2}}}r\right)+c_{2}Y_{0}\left(\sqrt{\frac{Z}{\theta \lambda^{2}}}r\right)$, with $c_{1}$ and $c_{2}$ constant [35,36] and $J_{0}\left(\sqrt{\frac{Z}{\theta \lambda^{2}}}r\right)=1+\sum_{m=1}^{\infty }\frac{{\left(-1\right)}^{m}{\left(\sqrt{\frac{Z}{\theta \lambda^{2}}}\right)}^{2m}}{2^{2m}{\left(m!\right)}^{2}}$ and $Y_{0}\left(\sqrt{\frac{Z}{\theta \lambda^{2}}}r\right)=\frac{2}{\pi }\left[\left(\gamma +\ln\left(0.5\left(\sqrt{\frac{Z}{\theta \lambda^{2}}}r\right)\right)\right)J_{0}\left(\sqrt{\frac{Z}{\theta \lambda^{2}}}r\right)+\sum_{m=1}^{\infty }\frac{{\left(-1\right)}^{m+1}H_{m}}{2^{2m}{\left(m!\right)}^{2}}{\left(\sqrt{\frac{Z}{\theta \lambda^{2}}}r\right)}^{2m}\right]$, where γ=0.5772 is the Euler–Mascheroni constant and $H_{m}=1+2^{-1}+3^{-1}+\times \times \times +m^{-1}$. Therefore, the general solution for (A61) becomes:

(A62) $$\begin{array}{c} u\left(r\right)=c_{1}\left[1+\sum_{m=1}^{\infty }\frac{{\left(-1\right)}^{m}{\left(\sqrt{\frac{Z}{\theta \lambda^{2}}}r\right)}^{2m}}{2^{2m}{\left(m!\right)}^{2}}\right]+\\ +\frac{2c_{2}}{\pi }\left[\left(\gamma +\ln\left(0.5\sqrt{\frac{Z}{\theta \lambda^{2}}}r\right)\right)\left[1+\sum_{m=1}^{\infty }\frac{{\left(-1\right)}^{m}{\left(\sqrt{\frac{Z}{\theta \lambda^{2}}}r\right)}^{2m}}{2^{2m}{\left(m!\right)}^{2}}\right]+\sum_{m=1}^{\infty }\frac{{\left(-1\right)}^{m+1}H_{m}}{2^{2m}{\left(m!\right)}^{2}}{\left(r\sqrt{\frac{Z}{\theta \lambda^{2}}}r\right)}^{2m}\right]. \end{array}$$

As r→0, then $J_{0}\to 1$. Thus, $\ln\left(0.5\sqrt{\frac{Z}{\theta \lambda^{2}}}r\right)$ in $Y_{0}$ exhibits a logarithmic singularity as r=0. It is worth nothing that a linear combination with $c_{1}\neq 0$ and $c_{2}=0$, the general integral becomes $u\left(r\right)=c_{1}\left[1+\sum_{m=1}^{\infty }\frac{{\left(-1\right)}^{m}{\left(\sqrt{\frac{Z}{\theta \lambda^{2}}}r\right)}^{2m}}{2^{2m}{\left(m!\right)}^{2}}\right]$. So a nontrivial solution (≠u(r)=0) for (A61) has been achieved. Exploiting Lemma A4, the uniqueness of the solution for (24) is not ensured.

## Appendix I. Proof of Theorem 7

As seen, (28) guarantees that (17) admits at least one solution. Moreover, from Theorem 4, it follows that the solution is also unique. Therefore, to ensure both existence and uniqueness, it is imperative to solve the system:

(A63) $$1+H^{6}<\frac{H\theta {\bar{\lambda }}^{2}}{2(h-d^{*})}\;\;\;\wedge \;\;\;1+H^{6}<\frac{\theta \lambda^{2}}{18}.$$

If, absurdly, we suppose that $\frac{1}{18}>\frac{1}{2(h-d^{*})}H{\bar{\lambda }}^{2}$ we would obtain

(A64) $$H<\frac{h-d^{*}}{9{\bar{\lambda }}^{2}}.$$

Finally, considering the scaling factors, (A64) becomes $H=\frac{z}{x}=\frac{z}{hx^{\prime }}<\frac{h-d^{*}}{9{\bar{\lambda }}^{2}}$. Hence, if we denote the dimensionless value of *H* by $H^{\prime }$, it follows that $H^{\prime }<\frac{h(h-d^{*})}{9{\bar{\lambda }}^{2}}$. Therefore, $H^{\prime }$ should be lower than a notably small amount that is incompatible with the definition of $H^{\prime }$ [19]. Thus, (A63) is equivalent to (36).

## Appendix J. Proof of Proposition 3

### Appendix J.1. Linearization of the Non-Linear First-Order Differential Equations System around the Equilibrium Configuration

Firstly, the model is written in the general form (39) in which

(A65) $$\bar{f}\left(u_{1}\left(x\right),u_{2}\left(x\right)\right)=u_{2}\left(x\right);\;\;\;\bar{g}\left(u_{1}\left(x\right),u_{2}\left(x\right)\right)=-\frac{1}{\theta \lambda^{2}}{\left(1+{\left(u_{2}\left(x\right)\right)}^{2}\right)}^{3}{\left(1-u_{1}\left(x\right)-d^{*}\right)}^{2}.$$

(39) can be written in matrix notation. That is

(A66) $$u\left(x\right)=\left(\begin{array}{c} u_{1}\left(x\right)\\ u_{2}\left(x\right) \end{array}\right),\;\;\;\dot{u}\left(x\right)=\left(\begin{array}{c} \frac{du_{1}\left(x\right)}{dx}\\ \frac{du_{2}\left(x\right)}{dx} \end{array}\right)\;\;\;f\left(x,u\left(x\right)\right)=\left(\begin{array}{c} \bar{f}\left(u_{1}\left(x\right),u_{2}\left(x\right)\right)\\ \bar{g}\left(u_{1}\left(x\right),u_{2}\left(x\right)\right) \end{array}\right),$$

(39) becomes:

(A67) $$\dot{u}\left(x\right)=f\left(x,u\left(x\right)\right).$$

#### Appendix J.1.1. A Suitable Change of Variables

To linearize the system, we exploit the following change of variable:

(A68) $$u_{1}\left(x\right)=u_{1}^{0}+\epsilon \xi \left(x\right);\;\;\;\;u_{2}\left(x\right)=u_{2}^{0}+\epsilon \eta \left(x\right)$$

with a small enough ϵ.

#### Appendix J.1.2. On the Use of the Taylor Series up to the First-Order

From (39), considering (A68) and $u_{1}^{0}$ and $u_{2}^{0}$ do not depend on *x*, one can write:

(A69) $$\frac{du_{1}\left(x\right)}{dx}=\epsilon \frac{d\xi (x)}{dx}=\bar{f}\left(u_{1}\left(x\right),u_{2}\left(x\right)\right)\;\;\;\wedge \;\;\;\frac{du_{2}\left(x\right)}{dx}=\epsilon \frac{d\eta (x)}{dx}=\bar{g}\left(u_{1}\left(x\right),u_{2}\left(x\right)\right).$$

Thus, developing in Taylor series $\bar{f}\left(u_{1}\left(x\right),u_{2}\left(x\right)\right)$, $\bar{g}\left(u_{1}\left(x\right),u_{2}\left(x\right)\right)$ (neglecting the terms of order higher), we achieve:

(A70) $$\left\{\begin{array}{c} \epsilon \frac{d\xi (x)}{dx}=\bar{f}\left(u_{1}^{0}+\epsilon \xi \left(x\right),u_{2}^{0}+\epsilon \eta \left(x\right)\right)\approx \bar{f}\left(u_{1}^{0},u_{2}^{0}\right)+\epsilon \frac{\partial \bar{f}\left(u_{1}^{0},u_{2}^{0}\right)}{\partial u_{1}}\xi \left(x\right)+\epsilon \frac{\partial \bar{f}\left(u_{1}^{0},u_{2}^{0}\right)}{\partial u_{2}}\eta \left(x\right)+o\left(\tau \right)\\ \epsilon \frac{d\eta (x)}{dx}=\bar{g}\left(u_{1}^{0}+\epsilon \xi \left(x\right),u_{2}^{0}+\epsilon \eta \left(x\right)\right)\approx \bar{g}\left(u_{1}^{0},u_{2}^{0}\right)+\epsilon \frac{\partial \bar{g}\left(u_{1}^{0},u_{2}^{0}\right)}{\partial u_{1}}\xi \left(x\right)+\epsilon \frac{\partial \bar{g}\left(u_{1}^{0},u_{2}^{0}\right)}{\partial u_{2}}\eta \left(x\right)+o\left(\tau \right) \end{array}\right.$$

in which $\tau =\epsilon \sqrt{\xi^{2}+\eta^{2}}$. Considering $\bar{f}\left(u_{1}^{0},u_{2}^{0}\right)=\bar{g}\left(u_{1}^{0},u_{2}^{0}\right)=0$, and neglecting the infinitesimal terms of order higher than τ, we obtain:

(A71) $$\frac{d\xi (x)}{dx}=\frac{\partial \bar{f}\left(u_{1}^{0},u_{2}^{0}\right)}{\partial u_{1}}\xi \left(x\right)+\frac{\partial \bar{f}\left(u_{1}^{0},u_{2}^{0}\right)}{\partial u_{2}}\eta \left(x\right)\;\;\;\wedge \;\;\;\frac{d\eta (x)}{dx}=\frac{\partial \bar{g}\left(u_{1}^{0},u_{2}^{0}\right)}{\partial u_{1}}\xi \left(x\right)+\frac{\partial \bar{g}\left(u_{1}^{0},u_{2}^{0}\right)}{\partial u_{2}}\eta \left(x\right).$$

**Remark** **A2.** *Even* (A71)*, as for* (39)*, admits a matrix representation. In fact, by placing:*

(A72) $$z=\left(\begin{array}{c} \xi (x)\\ \eta (x) \end{array}\right),\;\;\;\dot{z}=\left(\begin{array}{c} \frac{d\xi (x)}{dx}\\ \frac{d\eta (x)}{dx} \end{array}\right),\;\;\;A=\left(\begin{array}{cc} \frac{\partial \bar{f}\left(u_{1}^{0},u_{2}^{0}\right)}{\partial u_{1}} & \frac{\partial \bar{f}\left(u_{1}^{0},u_{2}^{0}\right)}{\partial u_{2}}\\ \frac{\partial \bar{g}\left(u_{1}^{0},u_{2}^{0}\right)}{\partial u_{1}} & \frac{\partial \bar{g}\left(u_{1}^{0},u_{2}^{0}\right)}{\partial u_{2}} \end{array}\right)$$

(A71) *becomes:*

(A73) $$\dot{z}=Az.$$

*Finally,* (A73) *is the linearized form of the non-linear system around* (A67)*.*

Here, considering (A65), *A* can be detailed as $\frac{\partial \bar{f}\left(u_{1}^{0},u_{2}^{0}\right)}{\partial u_{1}}=0;$∧$\frac{\partial \bar{f}\left(u_{1}^{0},u_{2}^{0}\right)}{\partial u_{2}}=1$∧$\frac{\partial \bar{g}\left(u_{1}^{0},u_{2}^{0}\right)}{\partial u_{1}}=\frac{2}{\theta \lambda^{2}}\left(h-d^{*}-1+d^{*}\right)=0$∧$\frac{\partial \bar{g}\left(u_{1}^{0},u_{2}^{0}\right)}{\partial u_{2}}=-\frac{6u_{2}}{\theta \lambda^{2}}{\left(h-d^{*}-1+d^{*}\right)}^{2}=0$. Therefore, (A71) (i.e., (A73)) becomes:

(A74) $$\frac{d\xi (x)}{dx}=\eta \;\;\;\;\wedge \;\;\;\;\frac{d\eta (x)}{dx}=0.$$

(A74), solved, provides $\xi \left(x\right)=k_{1}+kx$ and η(x)=k with $k_{1}$ and *k* constant.

**Remark** **A3.** (A70) *makes sense because $u_{1}$ and $u_{2}$, as proved in [16], are analytical functions allowing the linearization by $\frac{\partial \bar{f}\left(u_{1}^{0},u_{2}^{0}\right)}{\partial u_{1}}$, $\frac{\partial \bar{f}\left(u_{1}^{0},u_{2}^{0}\right)}{\partial u_{2}}$, $\frac{\partial \bar{g}\left(u_{1}^{0},u_{2}^{0}\right)}{\partial u_{1}}$ and $\frac{\partial \bar{g}\left(u_{1}^{0},u_{2}^{0}\right)}{\partial u_{2}}$.*

### Appendix J.2. Stability of the Equilibrium Configuration

#### Appendix J.2.1. Eigenvalues of the Coefficient Matrix and Algebraic Multiplicity

A=[01;00] and its eigenvalues become $\zeta_{1}=\zeta_{2}=0$. Thus, *A* has a single non-simple eigenvalue (its algebraic multiplicity m=2).

#### Appendix J.2.2. Point of Equilibrium: Evaluation of Its stability

For this purpose, we exploit the use the following result.

**Lemma** **A5.** *In a system as* (A73)*, if there is at least one eigenvalue with a positive real part or a not simple eigenvalue with zero real part, then the system is unstable [26].*

**Proposition** **A1.** (A74) *is not stable.*

**Proof.** Here, we achieve $\zeta_{1}=\zeta_{2}=\zeta =0$. Therefore, the only eigenvalue and m=2. Then, by Lemma A5, (A74) is not stable. □

The following result holds.

**Lemma** **A6.**
*If an equilibrium point of $\dot{z}=Az$ is unstable, then $\left(u_{1}^{0},u_{2}^{0}\right)$ of $\dot{u}\left(x\right)=f\left(x\right)$ is also unstable [26].*

**Proposition** **A2.**
*The model, of which the only equilibrium position is $\left(u_{1}^{0},u_{2}^{0}\right)=\left(h-d^{*},0\right)$. Then, this solution is unstable.*

**Proof.** It follows from Lemma A6. □

**Remark** **A4.**
*The membrane, when V is applied, deforms reaching a distance, in u(0), equal to $h-d^{*}$. However, this equilibrium is an unstable configuration.*

## Appendix K. Proof of Proposition 42

Considering that $\bar{\lambda^{2}}<\lambda^{2}$, it follows that $\lambda^{2}>\frac{2\left(h-d^{*}\right)\left(1+H^{6}\right)}{H\theta }$ when put into (36), we obtain:

(A75) $$V>\frac{\sqrt{2Td^{3}\left(h-d^{*}\right)\left(1+H^{6}\right)}}{\sqrt{\epsilon_{0}LH\theta }}=\sqrt{\frac{4Td^{3}\left(h-d^{*}\right)}{\epsilon_{0}\theta }}\sqrt{\frac{1+H^{6}}{H}}.$$

$\sqrt{\frac{4Td^{3}\left(h-d^{*}\right)}{\epsilon_{0}\theta }}\sqrt{\frac{1+H^{6}}{H}}$ in (A75) is less than the *V* necessary to achieve the inertia of the membrane. Thus

(A76) $${\left(V_{min}\right)}_{inertia}>\sqrt{\frac{4Td^{3}\left(h-d^{*}\right)}{\epsilon_{0}\theta }}\sqrt{\frac{1+H^{6}}{H}}.$$

## Appendix L. Proof of Proposition 6

Supposing absurdly that

(A77) $$\sqrt{\frac{2Td^{3}\left(h-d^{*}\right)}{0.5\epsilon_{0}\theta }}\sqrt{\frac{1+H^{6}}{H}}>\sqrt{\frac{2\left(h-d^{*}\right)d^{*}}{k\epsilon_{0}}}.$$

we achieve:

(A78) $$\frac{2d^{3}T}{0.5\sqrt{0.5\epsilon_{0}}}\sqrt{\frac{1+H^{6}}{H}}>\frac{\sqrt{d^{*}}}{\sqrt{k}}.$$

Observing that $u_{0}\leq d-d^{*}$ and $\frac{1}{\sqrt{u_{0}}}\geq \frac{1}{\sqrt{d-d^{*}}}$, it follows:

(A79) $$\frac{1}{\sqrt{k}}\geq \frac{\sqrt{p_{el}}}{\sqrt{d-d^{*}}}.$$

Thus, (A78), considering (A79) and taking into account that $d^{*}=\frac{d}{10}$, becomes:

(A80) $$\frac{\sqrt{p_{el}}}{T}\leq \frac{6d^{3}}{0.5\sqrt{0.5\epsilon_{0}}}\sqrt{\frac{1+H^{6}}{H}}.$$

Being H≈99[16], it follows that $\sqrt{\frac{1+H^{6}}{H}}\approx 97\times 10^{4}$. As $\epsilon_{0}=8.85\times 10^{-12}$ and $d\approx 10^{-9}$, (A80) becomes $\frac{\sqrt{p_{el}}}{T}\leq 0.95\times 10^{-6}$. Thus, the *T* would be a very large value as if the membrane were extremely rigid (condition physically not compatible with the usual membranes exploited in MEMS devices). Then, (A77) is false, so that (45) yields.

## Appendix M. Proof of Proposition 8

From (43) it follows:

(A81) $${\left(V_{max}\right)}_{permissible}<\sqrt{\frac{2(h-d^{*})}{\epsilon_{0}}}\sqrt{\frac{d^{*}}{k}}=\sqrt{\frac{2(h-d^{*})}{\epsilon_{0}}}\sqrt{\frac{h}{10k}}$$

so that $\sqrt{\frac{2(h-d^{*})}{\epsilon_{0}}}>{\left(V_{max}\right)}_{permissible}\sqrt{\frac{10k}{d}}$. Moreover, from (42), it makes sense to write:

(A82) $$\begin{array}{c} {\left(V_{min}\right)}_{inertia}>{\left(V_{max}\right)}_{permissible}\frac{d\sqrt{10k}}{\sqrt{0.5\theta }}\sqrt{\frac{1+H^{6}}{H}}\sqrt{T}. \end{array}$$

However, $p=k_{1}p_{el}$, with $k_{1}=4k$ and $k_{2}=2\sqrt{k}$, thus (A82) becomes [20]:

(A83) $${\left(V_{min}\right)}_{inertia}>{\left(V_{max}\right)}_{permissible}\frac{k_{2}}{2}\frac{d\sqrt{10}}{\sqrt{0.5\theta }}\sqrt{\frac{1+H^{6}}{H}}\sqrt{T}.$$

Furthermore, (A83) can be written as:

(A84) $$\begin{array}{c} {\left(V_{min}\right)}_{inertia}>{\left(V_{max}\right)}_{permissible}\frac{k_{2}d^{2}\sqrt{10}\sqrt{d}}{0.5\sqrt{2}\sqrt{0.5\epsilon_{0}}}\sqrt{\frac{1+H^{6}}{H}}T. \end{array}$$

## Appendix N. Proof of Proposition 9

Being $d-u\left(x\right)\geq d-\frac{k\epsilon_{0}V^{2}}{2d^{*}}\left(1-{\left(\frac{x}{L}\right)}^{2}\right)>\frac{2dd^{*}-k\epsilon_{0}V^{2}}{2d^{*}}$ it follows that $\frac{1}{d-u(x)}<\frac{2d^{*}}{2dd^{*}-k\epsilon_{0}V^{2}}$. Thus, $C=\epsilon_{0}\int_{-0.5}^{+0.5}\frac{dx}{d-u(x)}<\epsilon_{0}\int_{-0.5}^{+0.5}\frac{2d^{*}}{2dd^{*}-k\epsilon_{0}V^{2}}dx=\frac{2d^{*}}{2dd^{*}-k\epsilon_{0}V^{2}}$. Then, $W_{final}=\frac{1}{2}CV^{2}<\frac{1}{2}\frac{2d^{*}V^{2}}{2dd^{*}-k\epsilon_{0}V^{2}}$ so that $\Delta W=W_{final}-W_{initial}<\underset{f(V)}{\underset{︸}{\frac{1}{2}\frac{2d^{*}V^{2}}{2dd^{*}-k\epsilon_{0}V^{2}}-\frac{\epsilon_{0}LV^{2}}{d}}}.$ f(V), for usual values of *d*, $d^{*}$, *k* and $\epsilon_{0}$, and considering (46), is a continuous function. In fact, if in f(V), $2dd^{*}-k\epsilon_{0}V^{2}=0$, we would avchieve $V=\sqrt{\frac{2dd^{*}}{k\epsilon_{0}}}\approx 0.1503V$. However, this value is lower that $\inf{\left(V_{min}\right)}_{inertia}$, thus V≈0.1503V is not within the range of possible values for *V*. To calculate the stationary points for f(V), we set $\frac{df(V)}{dV}=\frac{2d^{*}k\epsilon_{0}V^{2}}{{\left(2dd^{*}-k\epsilon_{0}V^{2}\right)}^{2}}+\frac{2d^{*}}{2dd^{*}-k\epsilon_{0}V^{2}}-\frac{\epsilon_{L}}{d}=0$ from which, if $V^{2}=t$, we achieve $t=\frac{2dd^{*}\left(\epsilon_{0}\pm \sqrt{\epsilon_{0}}\right)}{k\epsilon_{0}^{2}}$ in which $2dd^{*}>0$, $k\epsilon_{0}^{2}>0$ and $\epsilon_{0}-\sqrt{\epsilon_{0}}<0$ obtaining $\frac{2dd^{*}\left(\epsilon_{0}-\sqrt{\epsilon_{0}}\right)}{k\epsilon_{0}^{2}}<0$. Therefore, V∈C, thus $t=\frac{2dd^{*}\left(\epsilon_{0}-\sqrt{\epsilon_{0}}\right)}{k\epsilon_{0}^{2}}$ must be discarded. Thus $V=\sqrt{\frac{2dd^{*}\left(\epsilon_{0}+\sqrt{\epsilon_{0}}\right)}{k\epsilon_{0}^{2}}}$. The negative root must be discarded, since this value of *V* would deform the membrane symmetrically with respect to the lower plate (condition physically impossible to obtain).

## Appendix O. Proof of Proposition 10

If absurdly $\frac{\epsilon_{0}}{2\theta^{2}\lambda^{2}}{\left(1+H^{2}\right)}^{3}{\left(h-d^{*}\right)}^{2}-\epsilon_{0}L\frac{V^{2}}{h}\leq \epsilon_{0}V^{2}\left\{\frac{Ld^{*}}{h(h-d^{*})}\right\}$, after some calculations, we obtain $\frac{h^{3}T{\left(1+H^{2}\right)}^{3}{\left(h-d^{*}\right)}^{2}}{2\theta^{2}0.5\epsilon_{0}^{3}}<\frac{V^{4}}{h-d^{*}}$ that, in dimensionless conditions, becomes $V>\sqrt[{4}]{\frac{3.10\times 10^{3}T}{\theta^{2}}}10^{5}$. That is, *V* should be greater than an extremely high amount. Then, it makes sense to write $\epsilon_{0}V^{2}\left\{\frac{Ld^{*}}{h(h-d^{*})}\right\}<\frac{\epsilon_{0}}{2\theta^{2}\lambda^{2}}{\left(1+H^{2}\right)}^{3}{\left(h-d^{*}\right)}^{2}-\epsilon_{0}L\frac{V^{2}}{h}$. Proposition 10 helps us to achieve an interesting increase for ΔW only depending on θ and on how to fix the membrane to the lower plate. In fact, we can write $\Delta W\leq \epsilon_{0}V^{2}\left\{\frac{0.5d^{*}}{h(h-d^{*})}\right\}<\frac{h^{3}T}{0.5\theta^{2}V^{2}}{\left(1+H^{2}\right)}^{3}{\left(h-d^{*}\right)}^{2}-\frac{0.5\epsilon_{0}V^{2}}{h}$ that, in dimensionless conditions, becomes $\Delta W<\underset{g(V)}{\underset{︸}{12\frac{T}{\theta^{2}V^{2}}-V^{2}}}$.
